# Prediction pipeline for discovery of regulatory motifs associated with *Brugia malayi* molting

**DOI:** 10.1371/journal.pntd.0008275

**Published:** 2020-06-23

**Authors:** Alexandra Grote, Yichao Li, Canhui Liu, Denis Voronin, Adam Geber, Sara Lustigman, Thomas R. Unnasch, Lonnie Welch, Elodie Ghedin

**Affiliations:** 1 Department of Biology, Center for Genomics and Systems Biology, New York University, New York, New York, United States of America; 2 School of Computer Science and Electrical Engineering, Ohio University, Athens, Ohio, United States of America; 3 Center for Global Infectious Disease Research, University of South Florida, Tampa, FL, Florida, United States of America; 4 Laboratory of Molecular Parasitology, Lindsley F. Kimball Research Institute, New York Blood Center, New York, New York, United States of America; 5 Department of Epidemiology, School of Global Public Health, New York University, New York, New York, United States of America; University of Pennsylvania, UNITED STATES

## Abstract

Filarial nematodes can cause debilitating diseases in humans. They have complicated life cycles involving an insect vector and mammalian hosts, and they go through a number of developmental molts. While whole genome sequences of parasitic worms are now available, very little is known about transcription factor (TF) binding sites and their cognate transcription factors that play a role in regulating development. To address this gap, we developed a novel motif prediction pipeline, Emotif Alpha, that integrates ten different motif discovery algorithms, multiple statistical tests, and a comparative analysis of conserved elements between the filarial worms *Brugia malayi* and O*nchocerca volvulus*, and the free-living nematode *Caenorhabditis elegans*. We identified stage-specific TF binding motifs in *B*. *malayi*, with a particular focus on those potentially involved in the L3-L4 molt, a stage important for the establishment of infection in the mammalian host. Using an *in vitro* molting system, we tested and validated three of these motifs demonstrating the accuracy of the motif prediction pipeline.

## Author summary

Diseases caused by parasitic worms such as the filariae are among the leading causes of morbidity in the developing world. Very little is known about how development is regulated in these vector-transmitted parasites. We have developed a computational method to identify motifs that correspond to transcription factor binding sites in the genome of the parasitic worm, *Brugia malayi*, one of the causative agents of lymphatic filariasis. Using this approach, we were able to predict stage-specific transcription factor binding sites involved in a stage of the molting process important for the establishment of the infection. We validated the role of these motifs using an *in vitro* molting system.

## Introduction

*Brugia malayi* is a mosquito-borne filarial nematode and one of the causative agents of lymphatic filariasis, commonly known as elephantiasis. Currently, 856 million people in 52 countries require preventative chemotherapy to stop the spread of the disease [1]. Transmission occurs when the mosquito vector introduces infective third-stage larvae (L3) during their blood meal. The larvae then migrate to the lymphatic vessels where they molt twice and develop into adults. Over their lifespan adult females produce millions of microfilariae (immature larvae) that circulate in the blood, allowing for continued transmission. Chronic lymphatic filariasis can cause permanent and disfiguring damage, characterized by lymphoedema (tissue swelling) and elephantiasis (tissue thickening) of the lower limbs, and hydrocele (scrotal swelling).

Over the past decade, a few parasitic nematode genomes have been sequenced, including *B*. *malayi* [2], *Loa loa* [3] and *Onchocerca volvulus* [4]. Transcriptomic experiments have helped quantify differentially expressed genes and their biological implications [5,6,7,8,9]. However, little is known about how these genes are regulated through cis-regulatory motifs. Motifs that have been characterized in *B*. *malayi* and that are available in the CIS-BP database [10] are purely bioinformatic predictions based on transcription factor binding site (TFBS) homology. *De novo* DNA motif discovery is an effective bioinformatic method for studying transcriptional gene regulation [11] and a number of motif discovery methods and tools currently exist. These include expectation-maximization methods, such as MEME [12] and Improbizer [13]; Gibbs sampling methods, such as BioProspector [14] and MotifSampler [15]; k-mer enumeration methods such as Weeder [16], DME [17], and DECOD [18]; ensemble methods such as W-ChIPMotifs [19], and GimmeMotifs [20]; and deep learning methods such as DanQ [21] and DeepFinder [22]. Based on the input types, motif discovery approaches can also be classified as either generative or discriminative. Generative motif discovery models use pre-defined background models (e.g., the Hidden Markov Model), while discriminative motif discovery models need to explicitly specify a set of background sequences. In this study, we developed Emotif Alpha that integrates a number of the current methods based on the aforementioned models, and filters the motifs using a Z-test, random forest feature importance, and sequence homology.

Gene promoter regions play a crucial role in gene regulation yet remain largely uncharacterized in *B*. *malayi*. Among the very few promoters that have been previously described and validated in *B*. *malayi* is that of Heat Shock Protein 70 (HSP70) [23]. A previous study showed that while the regulatory domains of the HSP70 promoter were similar to other eukaryotes, the core promoter domains appeared to be distinct [24]. Furthermore, nothing is known about motifs regulating developmentally expressed genes in *B*. *malayi*. There is thus a need for systematic identification, annotation, and experimental validation of *B*. *malayi* promoter motifs associated with gene regulation to better characterize filaria gene expression patterns. To better understand how promoter elements regulate stage-specific gene expression, we did differential gene expression analysis of the L3 to L4 molt, the first developmental step important for the establishment of infection in the mammalian host, and motif discovery using the Emotif Alpha pipeline. Several promoter motifs appeared to be associated with the regulation of the L3 to L4 molt. Our results provide an initial overview of the putative regulatory mechanisms in the filariae that could be targeted using novel intervention strategies for control.

## Results

### Stage-specific expression of serpins, peptidases, cysteine protease inhibitors, and structural constituents of the cuticle during the L3 to L4 molt

**Fig 1:**
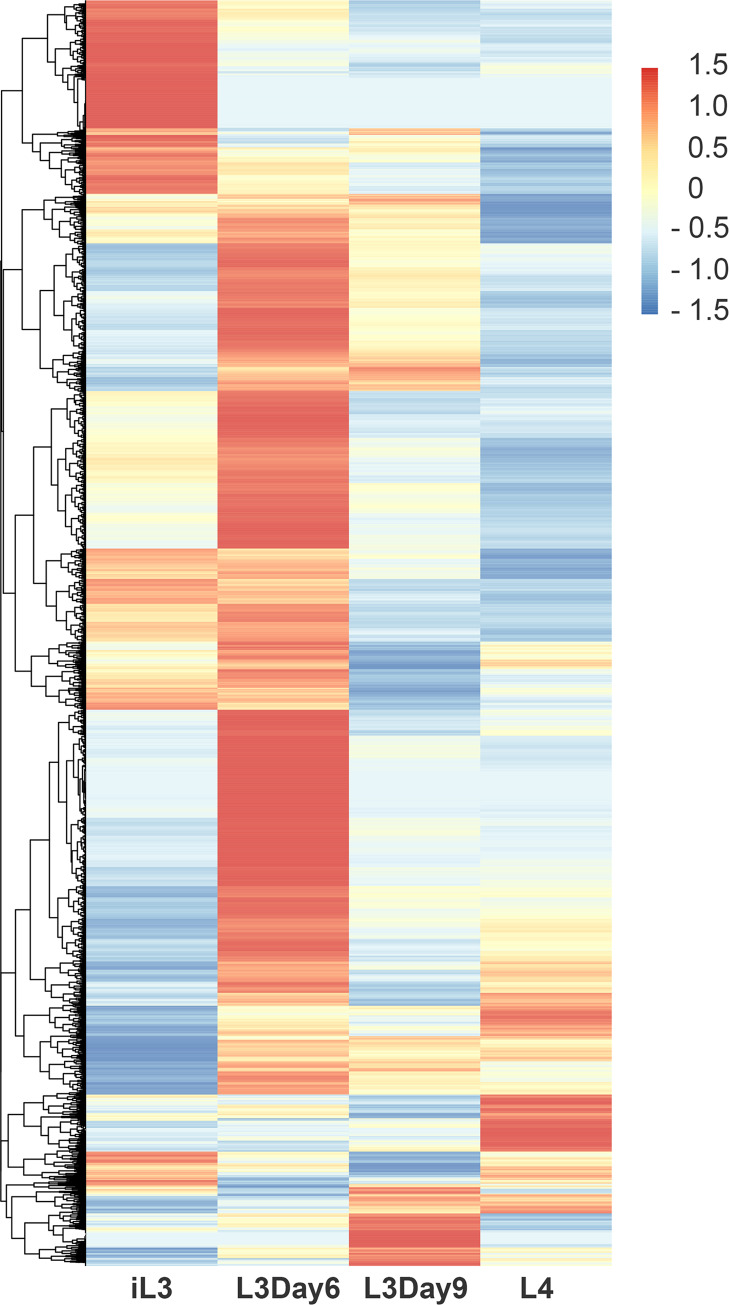
Expression of *Brugia malayi* genes during the L3 to L4 molt. Expression is in FPKMs and is Z-scale normalized by row prior to clustering. High expression is indicated by red and low expression by blue. Time-points included infective L3 larvae (iL3), L3 larvae at Day 6 of molting (L3D6), L3 larvae at Day 9 of molting (L3D9), and L4 larvae. Biological replicates have been combined.

Since the L3 to L4 molt is of particular interest because it corresponds to the life cycle stage when infective larvae establish infection, we focused in this study on identifying genes that are differentially expressed during this unique process. We used RNA-seq to profile transcription at different time points during the molt, collecting samples from the infective L3 (from mosquitoes), L3 Day 6, and L3 Day 9 worms recovered from infected gerbils (NCBI PRJNA557263). We combined this transcriptome data with previously published L4 data [7] that corresponds to Day 14 post infection of gerbils (**Table 1**). In total, 2.36 billion reads were generated, with 1.38 billion reads mapping to the *B*. *malayi* genome. Each biological replicate received an average of 272 million reads, with an average of 173 million reads that were successfully mapped (**Table 1**). Of the 11,841 *B*. *malayi* gene models, 87.6% were expressed in at least one stage of the L3 to L4 molt (**Fig 1**). The molting expression data shows unique stage-specific profiles for each stage of the molt with significant differential expression between days. Principle component analysis based on gene expression shows close clustering of biological replicates (**S1 Fig**).

**Table 1:** RNA-Seq summary.

Library^a^	Total reads (millions)^b^	Stage Total (millions)	Total Mapped Reads (millions)	Stage Total Mapped Reads (millions)	% *B*. *malayi* Genes Expressed
**L3a**	294	763	266	639	84
**L3b**	223		204		
**L3c**	246		169		
**L3D6a**	508	971	131	477	82.3
**L3D6b**	243		152		
**L3D6c**	220		194		
**L3D9a**	300	440	170	266	81.2
**L3D9b**	140		96		
**L4a**	104	195	47	96	72.4
**L4b**	90		49		

^a^Lower case a, b, and c refer to separate biological replicates.

^**b**^Total reads sequenced and mapped in each biological replicate at each developmental stage, L3 to L4.

We determined differentially expressed genes during the iL3 to L4 molt using both DESeq [25] and EdgeR [26] to perform pairwise comparisons between the four samples. To get a high confidence list of differentially expressed genes, we used the consensus of the two algorithms (FDR ≤0.01, log fold change ≥ 2.5) (**S1 Table**). All differentially expressed genes had a minimum coverage of 7 read counts in at least one stage. For the purposes of this study, we focused on the genes that were up-regulated at each stage of molting, as compared to the other stages, and did a gene annotation enrichment analysis for each stage comparison (**S2 Table**). We found that up-regulated genes in iL3 larvae—as compared to L3D6, L3D9, and L4—were enriched for annotations involving cysteine-type peptidase activity as well as serpin domains and serpin family proteins. Cysteine-type peptidases are essential for molting in *B*. *malayi* [27,28], and serpins are serine protease inhibitors that have previously been shown to be involved in immunomodulation and host immune evasion during infection [29]. We identified five different cysteine-type peptidases and two cysteine-type endopeptidase inhibitors that were upregulated in iL3 larvae. By day 6 of molting, structural constituents of the cuticle, including collagen (the main component of the cuticle) were enriched in the up-regulated gene sets. We also see the up-regulation of several metalloproteases. At day 9, genes involved in signaling were enriched among the up-regulated genes, as were several different metalloproteases. At day 14 (L4 larvae), we again see an enrichment of structural constituents of the cuticle. As for those enriched in L3 day 6 larvae, they are all mostly orthologs of *C*. *elegans* col (COLlagen) genes, which are themselves orthologs of human MARCO genes (macrophage receptor with collagenous structure). The structural constituents enriched at day 14 are, however, a completely unique set of collagen genes as compared to the genes observed at day 6. These stage-specific enrichments reflect the order of peptidases and structural constituents necessary for the building of a new L4 cuticle, the separation of the old L3 cuticle from the developing L4 cuticle, and the shedding of the old L3 cuticle.

### Identification of 12 motifs associated with transcription factor binding that are enriched in the L3 to L4 molt

**Fig 2:**
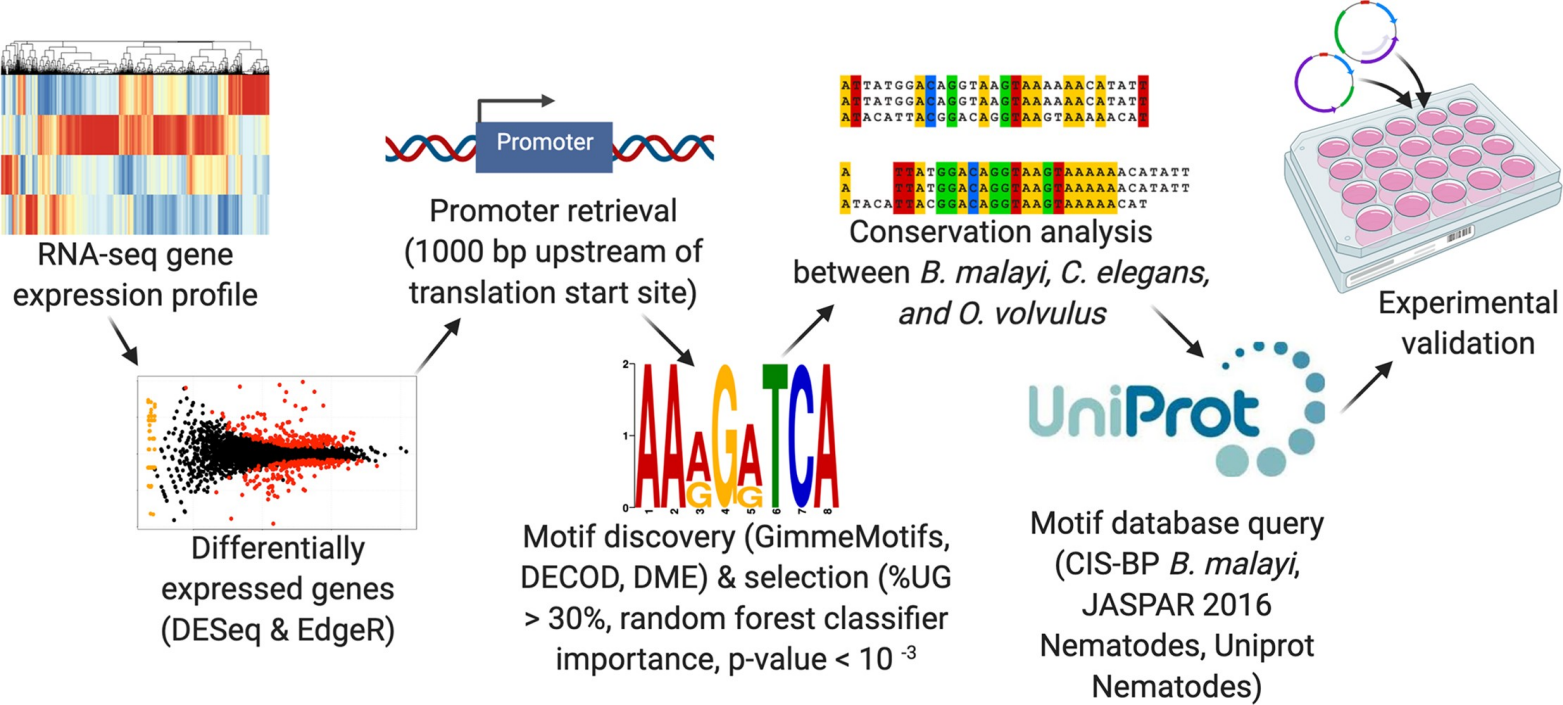
Workflow for promoter motif identification. The six main steps for motif discovery were: (1) generation of an RNA-seq profile; (2) determination of up-regulated genes for every pairwise comparison using DESeq2 (FDR<0.01) and EdgeR (adjusted P-value<0.01); (3) promoter retrieval: 1000 bp upstream of the translation start site; (4) ensemble motif discovery using GimmeMotifs (Homer, AMD, BioProspector, MDmodule, MEME, Weeder, GADEM, and Improbizer), DECOD, DME, and selection of enriched motifs: %UG > 30% and random forest classifier feature importance and over-representation p-value < 10^−3^; (5) TFBS conservation analysis between *B*. *malayi*, *C*. *elegans*, and *O*. *volvulus*; and (6) motif database query: CIS-BP *B*. *malayi*, JASPAR 2016 Nematodes, Uniprot Nematodes. Finally, a subset of those identified motifs were experimentally validated.

To better understand the regulatory program of *B*. *malayi* during the L3 to L4 molt, we analyzed statistically over-represented DNA motifs in regions upstream of genes that were upregulated during molting. To do so, we developed a motif identification pipeline called Emotif Alpha (**Fig 2**). First, we used the transcriptome data from the different stages of the L3 to L4 molt to generate using pair-wise comparisons lists of genes up-regulated at each stage of the molt. We then did a motif discovery analysis on each gene set using a combination of three motif discovery tools: GimmeMotifs, DME, and DECOD. GimmeMotifs is an ensemble of generative motif discovery tools—including Homer [30], AMD [31], BioProspecter, MDmodule [32], MEME, Weeder, GADEM [33], and Improbizer—while DME and DECOD are discriminative motif discovery tools. We did a discriminative motif discovery analysis by randomly selecting background promoter region sets from all *B*. *malayi* genes, excluding the differentially expressed genes. These background sets are three times larger than the foreground sets. We selected motif lengths between 6- and 15-mer. In total, we identified 20,025 motifs.

To select statistically significant motifs, we first assessed the motifs by a random forest classifier using scikit-learn [34]. The random forest algorithm uses bootstrap sampling and constructs a decision tree for each sub-sample. To evaluate the motifs, we used both Gini impurity [35] and information gain [36] criteria, and retained the union of the resulting top 40 motifs. We further filtered the motifs by foreground coverage (i.e. UG%), removing motifs occurring in less than 30% of the genes up-regulated at that stage of molting. We then used a Z-test to compare the frequency of a motif in the up-regulated genes with the expected frequency in the background promoters. Using a significance level (p-value) cutoff of 10^−3^, we selected 395 motifs (**S3 Table**).

We retrieved a collection of 163 known nematode transcription factor binding sites (TFBSs) from the MEME suite (http://meme-suite.org/), searching the motif databases JASPAR CORE 2016 nematodes [37], CIS-BP *Brugia malayi* [14], and Uniprobe worm [38]. We matched the remaining motifs to known TFBSs with TOMTOM [39]. If two motifs were matched to the same binding site and they were discovered from the same gene list, we considered them to be redundant and kept the one with the lowest over-representation p-value. This step narrowed our list down to 27 motifs that had matches to known binding sites.

**Fig 3:**
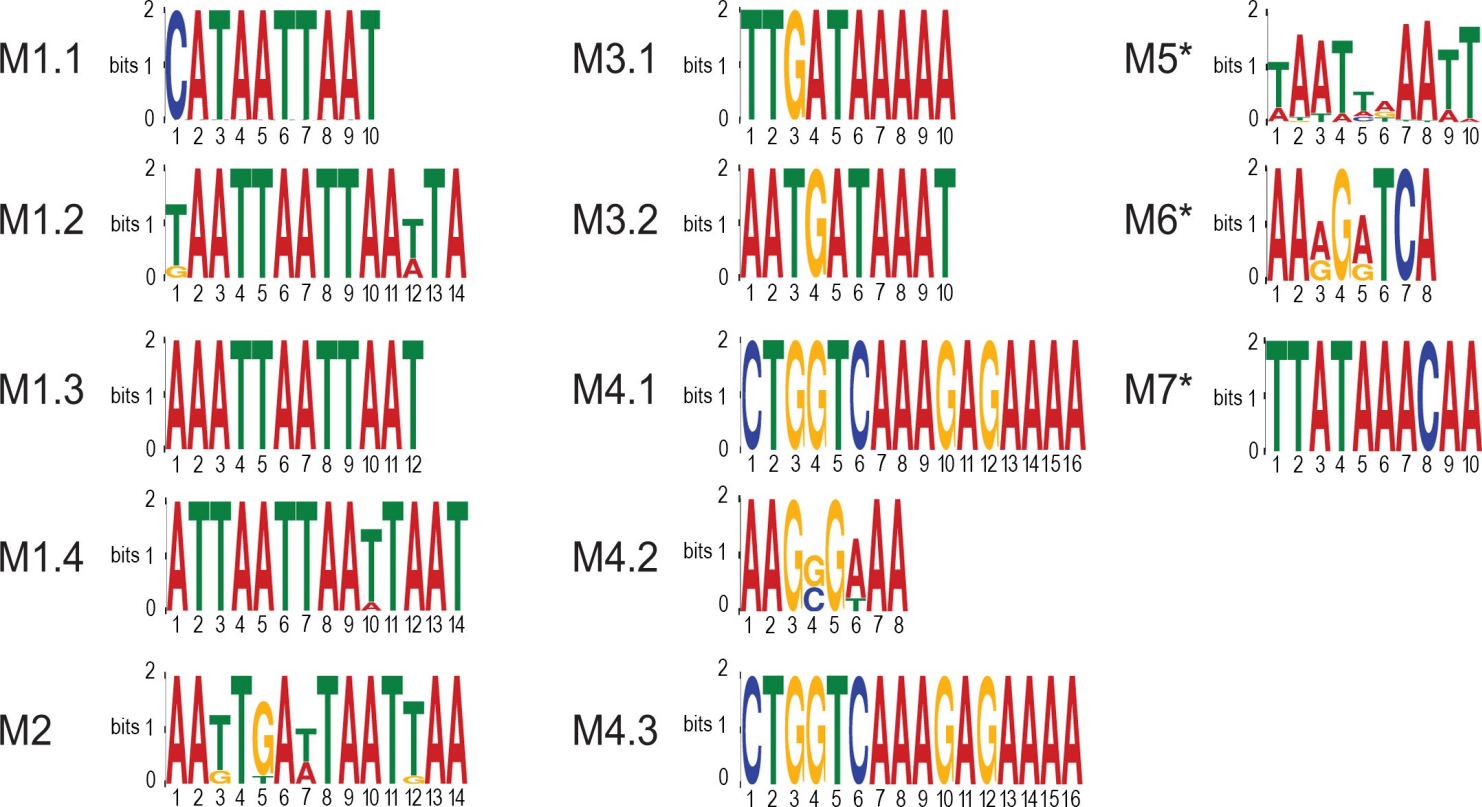
Logos of enriched promoter motifs over the L3 to L4 molt. Motifs found to be enriched (p-value < 10^−3^) in the upstream elements of up-regulated genes between different stages of molting and to be conserved in either *C*. *elegans* or *O*. *volvulus*. The * denotes the three motifs that have been validated experimentally. Note that M7 was included in the experimental validation because it passed 4 out of 5 filters, including the foreground coverage filter, the random forest filter, the known motif filter, and the conservation filter. However, it was not included in the 12 reported motifs due to its non-significant p-value.

We next performed a conservation analysis amongst nematodes using an adaptation of a published method [40]. We retrieved orthologous gene information among *B*. *malayi*, *C*. *elegans*, and *O*. *volvulus* from Wormbase ParaSite Biomart [41]. We extracted up to 1kb upstream from the translation start sites for *B*. *malayi* genes, assuming these regions would contain the promoter. We performed multiple sequence alignments using CLUSTALW2 [42] and defined a motif as conserved if it occurred at the same position in the orthologous promoter region alignment of either *C*. *elegans* or *O*. *volvulus*. This step resulted in 12 remaining motifs (**Fig 3, Table 2**) that were (1) enriched (p-value < 10^−3^) and (2) conserved in either *C*. *elegans* or *O*. *volvulus*. The frequency of motif occurrence in the putative promoter regions of up-regulated genes ranges from 33% to 94%. The fold enrichment, representing the ratio between motif frequencies in the up-regulated gene promoters and background promoters, ranges from 1.28 to 2.19.

**Table 2:** List of enriched promoter motifs over the L3 to L4 molt.

Name^a^	%UG^b^	Ratio^c^	P-value	Matched known motif	transcription factor (TF)	Function	Gene pair of conserved sites
M1.1 <L4,L3>	64	1.63	1.4*10^−4^	MA0928.1 (*C*. *elegans*)	zfh-2	Involved in hermaphrodite genitalia development, locomotion, nematode larval development and receptor-mediated endocytosis	[Bm4560, OVOC6906], [Bm856, OVOC3639]
M1.2 <L3D9,L3>	84.1	1.31	7.1*10^−5^				[Bm856, OVOC3639], [Bm17348, OVOC8896], [Bm17988, OVOC3292]
M1.3 <L3D6,L3>	94.4	1.28	7.6*10^−6^				[Bm2559, C34C6.3], [Bm2821, OVOC10446], [Bm17348, OVOC8896], [Bm3341, OVOC10396], [Bm4257, OVOC2553]
M1.4 <L3D9,L3D6>	84.7	1.55	9.6*10^−8^				[Bm7179, OVOC394], [Bm2270, OVOC2391]
M2 <L3D6,L4>	93.3	1.31	2.9*10^−5^	MA0927.1 (*C*. *elegans)*	vab-7	Required for DB motorneuron identity and posterior DB axonal polarity	[Bm4904, OVOC2123]
M3.1 <L3D9,L3D6>	80.6	1.33	3.0*10^−4^	MA0542.1 (*C*. *elegans*)	elt-3	Controlling hypodermal cell differentiation	[Bm2802, OVOC9504]
M3.2 <L3D9,L3>	81.8	1.32	8.3*10^−5^				[Bm10655, C28A5.3, OVOC9600]
M4.1 <L4,L3D6>	34.8	2.19	4.9*10^−4^	MA0537.1 (*C*. *elegans*)	blimp-1	Loss of blmp-1 activity via deletion mutation has been reported to result in small, dumpy animals with abnormal fat content	[Bm2802, OVOC9504], [Bm6190, OVOC827]
M4.2 <L4,L3>	56	1.71	1.6*10^−4^				[Bm7019, OVOC7405], [Bm6190, OVOC827]
M4.3 <L3D9,L3D6>	38.9	2.01	1.0*10^−5^				[Bm2802, OVOC9504], [Bm7179, OVOC394]
M5* <L3,L3D6>	93.3	1.23	2.9*10^−4^	M5348_1.02 (CIS-BP *Brugia* inferred)	Bm8528	Retinal homeobox protein Rx3	[Bm1938, OVOC2080], [Bm8228, C27D6.4], [Bm1559, OVOC7210], [Bm4184, OVOC3386]
M6* <L3,L3D6>	33.3	2.03	3.0*10^−5^	M5221_1.02 (CIS-BP *Brugia* inferred)	Bm4429	Involved in regulation of transcription, DNA-templated and steroid hormone mediated signaling pathway	[Bm1938, OVOC2080], [Bm6642, Y48B6A.12]
M7* <L3,L3D9>	54.5	1.19	6.1*10^−2^	M0739_1.02 (CIS-BP inferred)	Bm3608	Involved in cell growth, proliferation, differentiation, and longevity.	[Bm4184, OVOC3386]

^a^The text listed in <> brackets correspond to comparisons between stages of molting where upstream elements of upregulated genes were found to be enriched with specific motifs.

^b^Frequency of a motif in up-regulated gene promoters.

^c^Relative frequency of a motif in up-regulated gene promoters vs. background promoters. *Denotes the three motifs validated experimentally.

The 12 selected motifs matched known binding sites for 6 transcription factors in *C*. *elegans* (**Table 2**), all of which are involved in development, aging, and/ or movement. Motifs M1.1, M1.2, M1.3 and M1.4 matched a zinc-finger protein, zfh-2, which is involved in hermaphrodite genitalia development, locomotion, nematode larval development and receptor-mediated endocytosis. Motif M2 matched vab-7, which is associated with DB motor neuron identity and posterior DB axonal polarity. Motifs M3.1 and M3.2 matched elt-3, which is related to aging [43]. Motifs M4.1, M4.2, and M4.3 matched blmp-1. M5 matched a homeobox protein, Bm8528, and M6 matched a nuclear receptor, Bm4429. The 12 motifs are conserved in either *C*. *elegans* or *O*. *volvulus* (**Table 2**, last column; **S4 Table**). Moreover, the occurrence of M3.2 in the Bm10655 promoter region is conserved in orthologs in both *C*. *elegans* (promoter of C28A5.3) and *O*. *volvulus* (promoter of OVOC9600). We find that a number of the *B*. *malayi* orthologs to the *C*. *elegans* transcription factors that we predict could bind the predicted TFBS are differentially expressed as well (**S5 Table**).

The motif analysis reveals how some of the differential expression of different proteases may be orchestrated during the L3 to L4 molt. Motif M1.4 is found in the promoter region of Bm2270, a predicted metalloprotease significantly up-regulated in L3D9 worms (**S4 Table)**. Bm2270 is an ortholog of nas-37 in *C*. *elegans* and has been shown to be involved in collagen and cuticulin-based cuticle development and ecdysis. Motif M5 is found in the promoter region of Bm1938 and is predicted to encode a serpin (**S4 Table)**. Bm1938 is one of the serpins that was found to be significantly up-regulated in the iL3 larvae.

### L3 stage-specific transcription factor binding motifs can be validated *in vitro*

**Fig 4:**
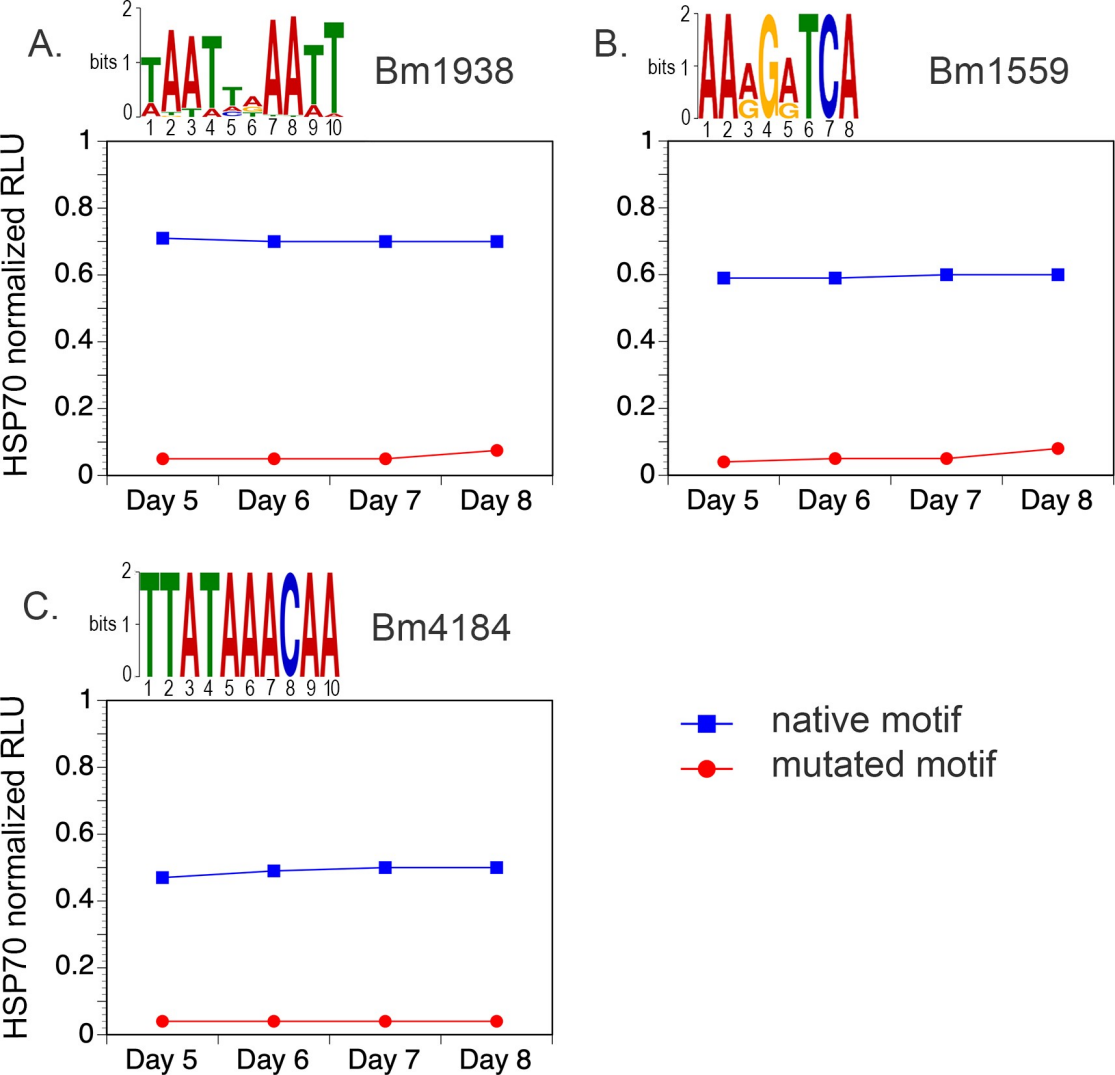
Promoter motif validation. A) Promoter motif validation in L3 worms that were molting *in vitro* using the native promoter of Bm1559 and a mutated motif M5 version of the same promoter. B) Promoter motif validation using the native promoter of Bm1938 and a mutated motif M6 version of the promoter. C) Promoter motif validation using the native promoter of Bm4184 with a mutated motif M7 version of the promoter. A total of four wells, each containing 100 L3, were transfected with each construct, as previously described [44]. Parasites in each well were harvested on days 5–8 and firefly luciferase activity determined as previously described [23]. In each panel luciferase activity obtained from the constructs is normalized against parasites transfected with a construct containing the Bm HSP70 promoter driving the expression of the firefly luciferase reporter.

Three of the motifs (M5, M6, and M7) were chosen for validation based on their enrichment in the promoters of genes up-regulated in the mid to late stages of the L3 to L4 molt. Three separate genes, each containing one chosen motif, were tested. The 1 kb upstream region of each gene was amplified from *B*. *malayi* genomic DNA and cloned upstream of the firefly luciferase reporter gene in the expression vector pGL3 Basic [23]. *B*. *malayi* L3 were then transfected with the constructs in a co-culture system, as previously described [44]. The parasites were induced to molt *in vitro* and then assayed for luciferase activity. The number of relative light units (RLUs) observed were normalized to those obtained from parasites transfected in parallel with a construct consisting of the *B*. *malayi* HSP70 promoter driving the expression of the firefly luciferase reporter [44]. The experiment was performed with both the native promoter and a mutant promoter where the nucleotides of the motif had been randomly shuffled. All of the native promoters produced significant amounts of reporter luciferase activity in the molting parasites (ranging from 40–70% of the activity produced by the HSP70 construct transfected positive controls; **Fig 4**). However, when the putative motifs were mutated, the activity in all the promoters tested decreased by 80–90% (**Fig 4**).

## Discussion

Parasitic nematodes such as *B*. *malayi* maintain a complicated lifecycle involving both an insect and a mammalian host, and undergo a number of molts. The L3 to L4 molt that occurs immediately upon infection of the mammalian host is of particular interest as it marks the establishment of infection and thus represents an attractive point for drug intervention. Little is known, however, about how *B*. *malayi* regulates the transitions between these stages. Prior to our study, nothing was known about promoter motifs that regulate developmentally expressed genes in *B*. *malayi*. Because stage transitions rely on precise transcriptional control through the interaction of transcription factors and their binding sites, we set out to characterize the potential transcription factor binding motifs of these parasites and to identify motifs that contribute to stage-specific expression of genes involved in early worm development in the mammalian host.

We found a number of enriched motifs and were able to define both conserved motifs across molting as well as stage-specific motifs. While some of the motifs we identified are conserved in other nematodes, such as *C*. *elegans*, a number of motifs represent novel binding sites potentially reflecting the differences in development and the parasitic lifestyle. It is known that molting is regulated by an ecdysone-like response system [45,46,47,48]. Two of the identified motifs and their cognate TFs appear to be related to the ecdysone response. For example, zfh-2, the transcription factor predicted to bind four of our identified motifs, is a common cofactor implicated in ecdysone signaling in *D*. *melanogaster* [49]. Blimp-1, the transcription factor predicted to bind three of our identified motifs, is an ecdysone-inducible repressor that is essential for the prepupal development in *Drosophila* [50]. Validation results suggest that our pipeline is able to identify biologically-relevant motifs involved in molting. This analysis provided biological insight into the development of the parasite as well as the identification of novel drug targets.

Future studies should expand this analysis across the lifecycle of the nematode at different stages of development, in its different hosts (i.e. human vs. mosquito). While the validation results from our *in vitro* system are promising, we must assume there are biological differences to molting *in vivo*. Transcriptomic data from other life stages already exist and can be used to predict motifs; however, validation at other stages *in vivo* will prove more difficult. This is due, in large part, to the fact that the human filariae are obligate parasites without a free-living stage. This has limited the ability to genetically manipulate these stages in ways necessary to validate predictions made by transcriptomic analyses. Until recently, studies of promoter structure and function were limited by the fact that the only way to transfect *B*. *malayi* involved biolistic transfection of isolated embryos [23] which were developmentally incompetent. While this system has been used to identify the conserved motifs necessary for promoter function in constitutive promoters [24,51,52,53] and cis acting regulatory regions in some regulated promoters [54,55], these studies were limited to genes expressed in embryos. However, with recent innovations in filarial transgenics, it is now possible to efficiently produce stably transfected developmentally competent infective larvae, which can in turn be used to produce transgenic parasites in which transgenes are stably integrated into the parasite genome [44]. This study represents the first application of this new technology to the study of promoter function in a lifecycle stage other than isolated developmentally incompetent embryos. The ability to produce stable transgenic parasite lines will permit *in vivo* functional testing of promoter motifs for stage-specific expressed genes in all lifecycle stages of the parasite.

## Materials and Methods

### Transcriptomic study design

All parasites were obtained from FR3 (Filariasis Research Reagent Resource Center; BEI Resources, Manassas, VA, USA) where they were isolated from infected gerbils (*Meriones unguiculatus*) or mosquitoes (*Aedes aegypti*). Worms were flash-frozen and shipped to the New York Blood Center for processing. For transcriptomic sequencing, infective third-stage larvae (iL3) were recovered from mosquitoes and mammalian stage larvae were recovered from gerbils at 6 and 9 days post infection (dpi). At 6 dpi, larvae are typically undergoing the molt from L3 to L4, while by 9 dpi some worms may be finished molting [56] while others may not finish until 10 dpi [57]. Data was combined with previously published stages 14 dpi (L4) [7].

### RNA isolation, library preparation and sequencing

Total RNA was prepared from *B*. *malayi* worms as previously described [7]. RNA was prepared from 3 biological replicates of infective L3 (iL3; 2000 larvae each), 3 replicates of 6 dpi larvae (1500 each) and 2 replicates of 9 dpi larvae (1300 each). *B*. *malayi* worms were homogenized in Trizol (ThermoFisher) using a hand-held pestle in 1.5mL tubes containing the worms. Total RNA was extracted by organic extraction using Trizol and the PureLink RNA mini kit (ThermoFisher) and after being treated with DNaseI (New England Biolabs). Ribosomal RNA (rRNA) depletion was performed using Terminator (Epicentre), a 5’-phosphate-dependent exonuclease that degrades transcripts with a 5’ monophosphate. Libraries were prepared using the NEBNext Ultra II RNA Library Prep Kit for Illumina (New England Biolabs) according to manufacturer instructions. Library quality was assessed using a D1000 ScreenTape Assay (Agilent) prior to sequencing. Library concentrations were assessed using the qPCR library quantification protocol (KAPA biosystems). Libraries were sequenced on the Illumina NextSeq500 platform with 150bp paired-end reads. To minimize the confounding effects of lane-to-lane variation, libraries were multiplexed and sequenced with technical replicates on multiple lanes. Each biological replicate received an average of 135 million mapped reads (PRJNA557263).

### Sequencing alignment and expression analysis

Read quality was assessed using FastQC (Babraham Bioinformatics). Sequence reads from each sample were analyzed with the Tuxedo suite of tools [58,59,60]. Reads were mapped with Tophat2’s Bowtie2-very-sensitive algorithm to the annotated *B*. *malayi* genome assembly (WormBase.org). The resulting BAM files were then used with HtSeq to obtain raw read counts. Differential gene expression analysis was performed using both DESeq and EdgeR, and the overlapping genes with FDR < 0.01 were retained. Up-regulated genes were characterized for each pairwise comparison between L3, L3 Day 6 (L3D6), L3 day 9 (L3D9), and L4 worms. For example, <L3,L4> refers to the up-regulated genes in L3 compared to L4. Two pairs of comparisons, <L3D9,L4> and <L4,L3D9>, were dropped due to the limited number of up-regulated genes (< = 3) passing our stringent filters, possibly due to the L3D9 being actually younger L4. The up-regulated gene lists were filtered using log2 fold change (logFC) with the following thresholds: |logFC| = 7 for <L3D6,L3>, <L3D6,L3D9>, <L3D6,L4>; |logFC| = 4 for <L3D9,L3>, <L3,L3D6>, <L3,L3D9>, <L3,L4>, <L4,L3>; |logFC| = 2.5 for <L3D9,L3D6>, <L4,L3D6>. The reason for varying the threshold was that the number of up-regulated genes in each list varied significantly. For motif discovery tools to search efficiently, the number of sequences were limited to less than one hundred. In total, 10 up-regulated gene lists were used for motif discovery (**S1 Table)**. Functional annotation enrichment analysis of the upregulated gene lists was done using DAVID 6.8 [61].

Potential promoter sequences were retrieved from WormBase ParaSite Biomart [41] web interface, capturing the 1000bp upstream of the translation start site for each gene.

### The Emotif Alpha pipeline for regulatory motif identification

The Emotif Alpha pipeline (freely available at: https://github.com/YichaoOU/Emotif_Alpha) was developed to automate motif discovery analysis for the 10 up-regulated gene lists. This pipeline was written in python and was applied to perform all aforementioned motif analyses. The motif discovery step used multiple tools and was run in parallel at the Ohio SuperComputer Center. Motif length search was from 6 to 14. Motif scanning was done using FIMO [62] with a default p-value threshold of 10^−4^. We implemented 5 different motif filters: (1) Foreground coverage (i.e., UG%) was defined as the proportion of up-regulated gene promoters containing the given motif. We set a minimal foreground coverage at 30%; (2) Motifs were then filtered by a random forest classifier. The union of the top 40 motifs that resulted from either Gini impurity or information gain criterion was retained; (3) Motif enrichment p-value was calculated using Z-test and the cutoff was 10^−3^; (4) Known motif filter was performed using TOMTOM and a collection of 163 known nematode TFBSs. The motif similarity p-value threshold was 10^−4^; (5) Conservation analysis was performed using a method described in [40]. Only conserved motifs were kept.

### *In vitro* validation of promoter transcription motifs

The putative TF motifs M5, M6, and M7 were chosen for validation based on their enrichment in the promoters of genes upregulated in the mid to late stages of the L3 to L4 molt. Three different genes, each containing one of the chosen motifs, were used for the validation assay. As previously described [23], we amplified the 1 kbp region upstream of each gene from *B*. *malayi* genomic DNA and cloned it upstream of the firefly luciferase reporter gene in the expression vector pGL3 Basic. We then transfected *B*. *malayi* L3 larvae with the constructs in a co-culture system, as previously described [44]. The parasites were induced to molt by the addition of ascorbic acid on day 5, and parasites were assayed for luciferase activity on days 5–8, as by day 10 the molting was complete. We normalized the number of RLUs observed to those obtained from parasites transfected in parallel with a construct consisting of the *B*. *malayi* HSP70 promoter driving the expression of the firefly luciferase reporter [23] to control for accumulation of the firefly luciferase over time during the duration of the experiment. We did the experiment with both the native promoter and a mutant promoter where the nucleotides of the motif had been randomly shuffled (**S6 Table**). Only one replicate was carried out for each motif validation.

## Supporting information

S1 Fig Principle component analysis of biological replicates based on stage-specific expression. (PDF)

S1 Table Stage comparisons of upregulated genes. (XLSX)

S2 Table Functional annotation enrichment of gene lists upregulated during molting. (XLSX)

S3 Table List of statistically significant *de novo* motifs identified. (PDF)

S4 Table Wormbase annotations for the gene pairs of conserved sites from Fig 3. (XLSX)

S5 Table Expression of *B*. *malayi* transcription factors predicted to interact with the identified motifs in Fig 3. (XLSX)

S6 Table Primers used for validation mutagenesis. (XLSX)
