# Supplementary figures and images for: Prediction pipeline for discovery of regulatory motifs associated with Brugia malayi molting
Source: PLoS Negl Trop Dis. 2020 Jun 23;14(6):e0008275. doi: 10.1371/journal.pntd.0008275 (PMC7337397; doi:10.1371/journal.pntd.0008275)

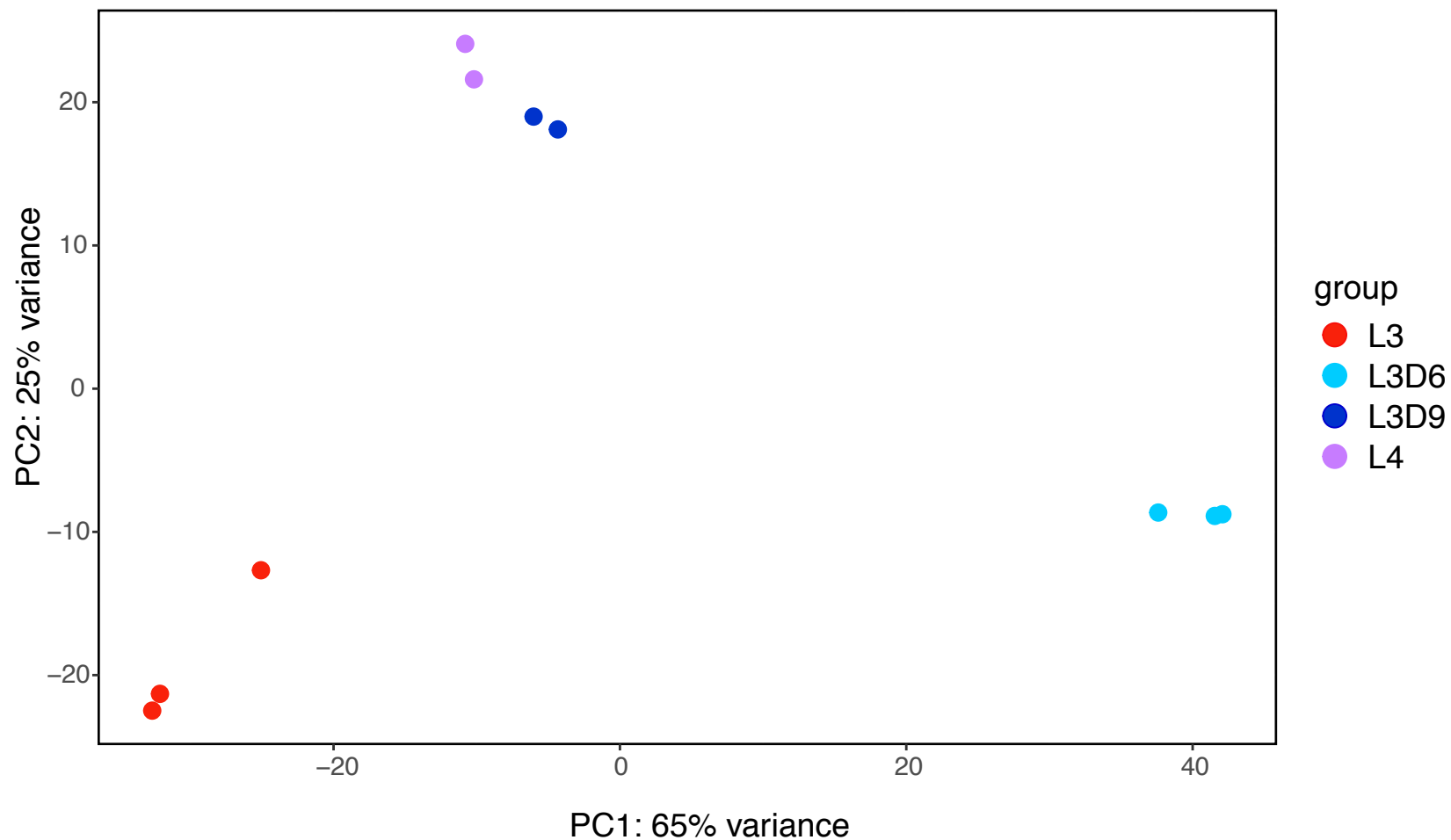

Supplement: S1 Fig — (PDF) [file pntd.0008275.s001.pdf]
