# Supplementary material for: Prediction pipeline for discovery of regulatory motifs associated with Brugia malayi molting
Source: PLoS Negl Trop Dis. 2020 Jun 23;14(6):e0008275. doi: 10.1371/journal.pntd.0008275 (PMC7337397; doi:10.1371/journal.pntd.0008275)
Supplement: S3 Table — (PDF) [file pntd.0008275.s004.pdf]

Table S3. List of statistically significant *de novo* motifs identified

| Motif logo | Motif Name | Motif p_value | Discovery stage |
|---|---|---|---|
| 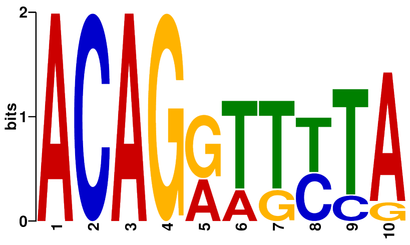 | DME_ACAGRWKYYR | 5.83E-26 | L3D6_L3D9 |
| 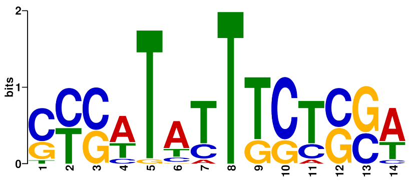 | gimme_119_MEME_6_w14 | 3.12E-18 | L3D6_L3D9 |
| 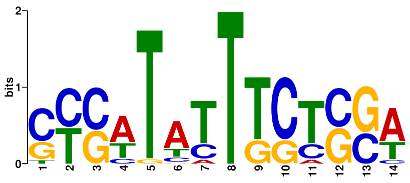 | gimme_149_MEME_6_w14 | 8.63E-18 | L3D6_L3D9 |
| 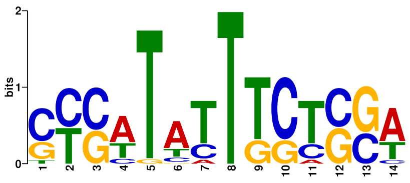 | gimme_159_MEME_6_w14 | 1.58E-16 | L3D6_L3D9 |
| 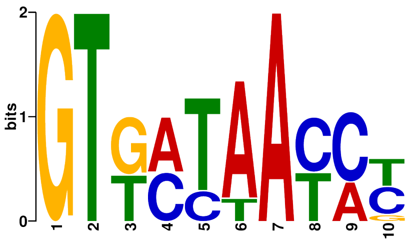 | DME_GTKMYWAYMB | 7.16E-16 | L3_L3D6 |
| 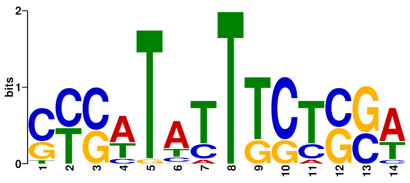 | gimme_139_MEME_6_w14 | 2.47E-15 | L3D6_L3D9 |
| 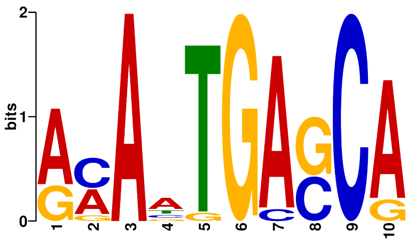 | gimme_137_MEME_8_w10 | 5.56E-14 | L3D6_L3 |
| 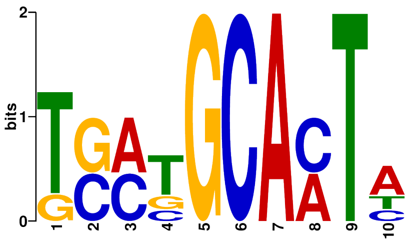 | DME_KSMBGCAMTH | 6.98E-14 | L3_L3D6 |
| 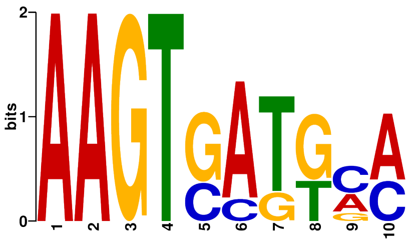 | DME_AAGTGMKKVM | 8.94E-14 | L3_L3D6 |
| 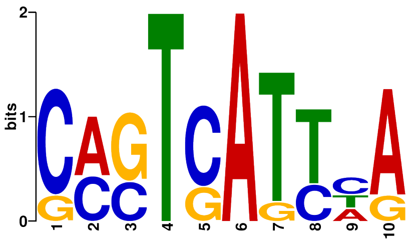 | DME_SMSTSATYHR | 1.16E-12 | L3_L3D9 |

| Motif logo | Motif Name | Motif p_value | Discovery stage |
|---|---|---|---|
| 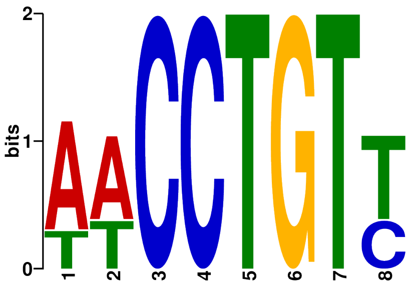 | DME_WWCCTGTY | 1.00E-11 | L3D6_L3D9 |
| 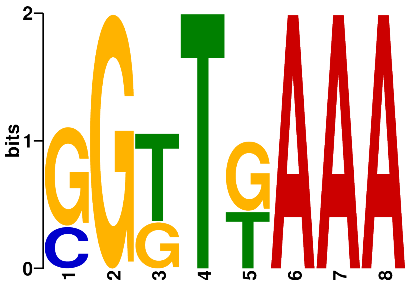 | DME_SGKTKAAA | 1.87E-11 | L4_L3 |
| 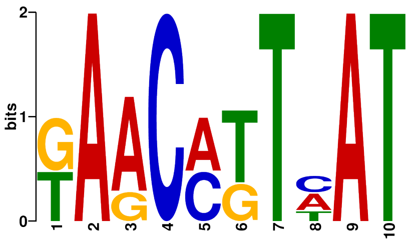 | DME_KARCMKTHAT | 2.54E-11 | L3_L3D9 |
| 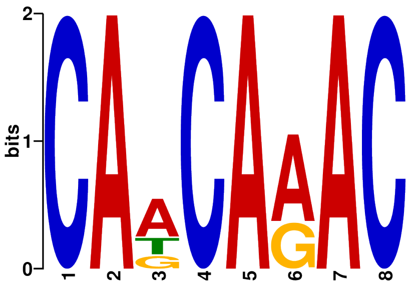 | DME_CADCARAC | 4.97E-11 | L3_L4 |
| 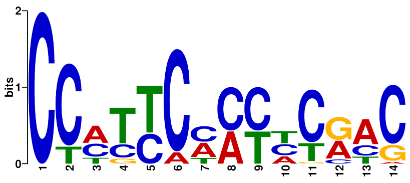 | gimme_153_MEME_4_w14 | 6.44E-11 | L3D6_L3 |
| 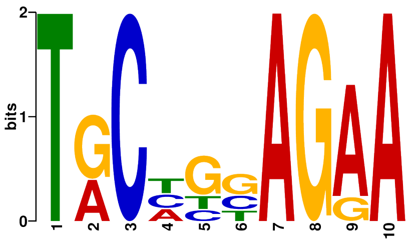 | DME_TRCHBBAGRA | 1.12E-10 | L3_L3D6 |
| 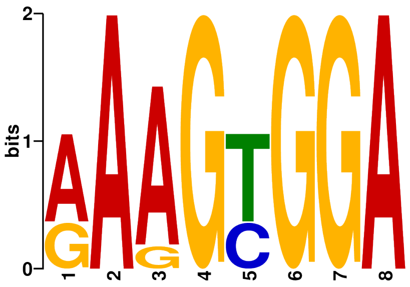 | DME_RARGYGGA | 1.48E-10 | L3D6_L4 |
| 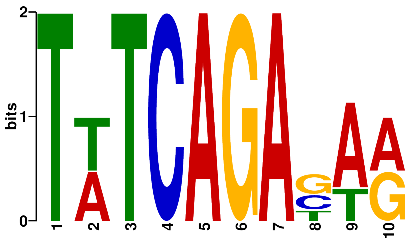 | DME_TWTCAGABWR | 1.66E-10 | L3D6_L4 |

| Motif logo | Motif Name | Motif p_value | Discovery stage |
|---|---|---|---|
| 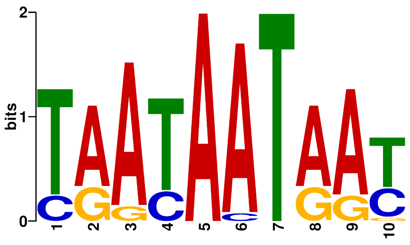 | DECOD_Motif5_10 | 2.80E-10 | L3D6_L3 |
| 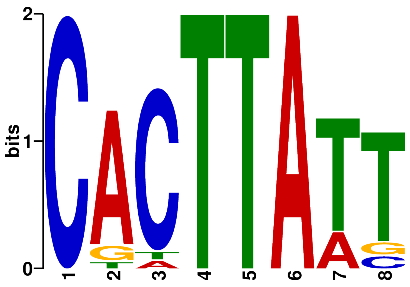 | DECOD_Motif6_8 | 4.14E-10 | L3_L4 |
| 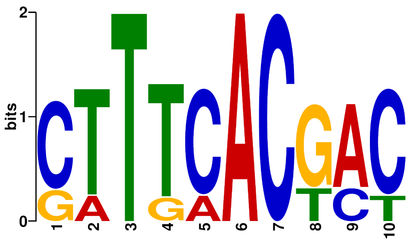 | DME_SWTKMACKMY | 6.07E-10 | L3_L3D6 |
| 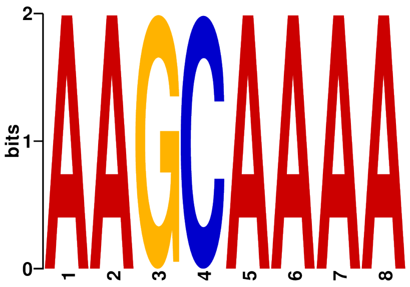 | DME_AAGCAAAA | 6.90E-10 | L3D9_L3D6 |
| 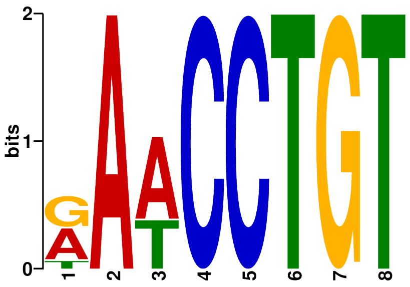 | DME_DAWCCTGT | 6.97E-10 | L3D6_L3D9 |
| 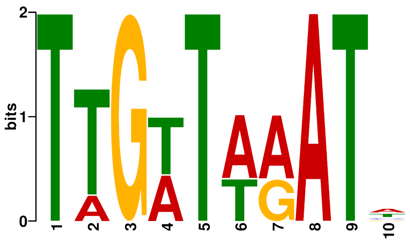 | gimme_26_BioProspector_w10_5 | 8.97E-10 | L3D9_L3 |
| 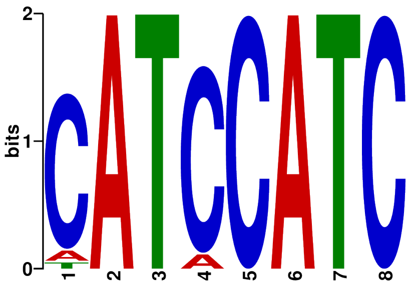 | DME_HATMCATC | 1.04E-09 | L3D6_L4 |
| 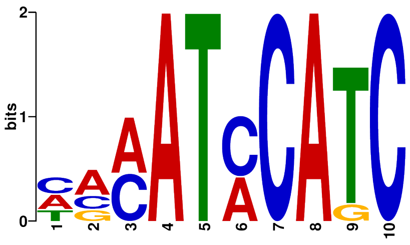 | DME_HVMATMCAKC | 1.19E-09 | L3D6_L4 |

| Motif logo | Motif Name | Motif p_value | Discovery stage |
|---|---|---|---|
| 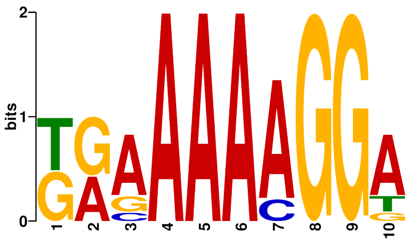 | DME_KRVAAAMGGD | 1.55E-09 | L3D6_L3 |
| 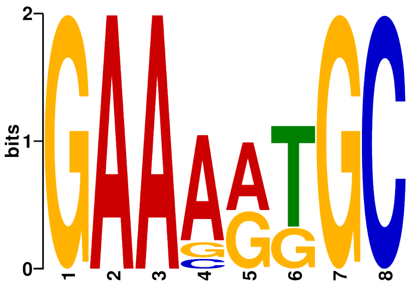 | DME_GAAVRKGC | 1.60E-09 | L3D9_L3D6 |
| 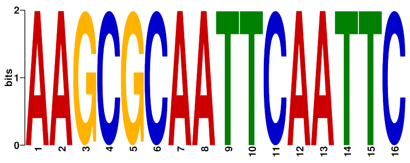 | DME_AAGCGCAATTCAATTC | 1.68E-09 | L3D9_L3 |
| 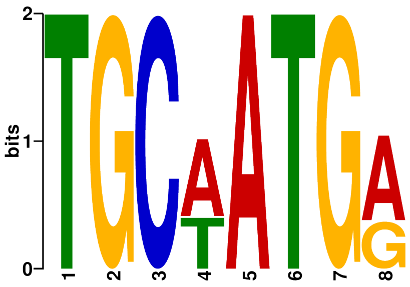 | DME_TGCWATGR | 1.86E-09 | L3D9_L3D6 |
| 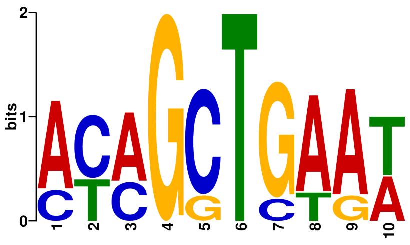 | DME_MYMGCTSWRW | 2.78E-09 | L3_L3D6 |
| 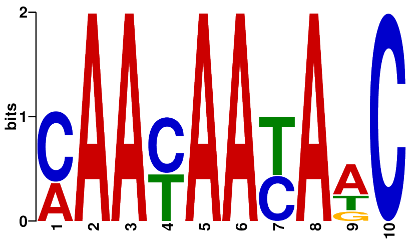 | DME_MAAYAAYADC | 2.96E-09 | L3D6_L4 |
| 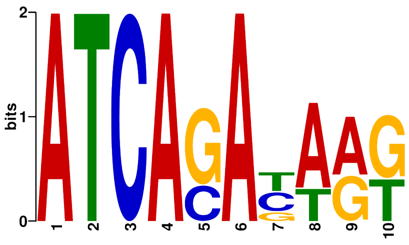 | DME_ATCASABWRK | 3.65E-09 | L3D6_L4 |
| 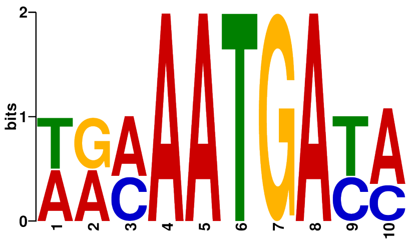 | DME_WRMAATGAYM | 1.40E-08 | L3D9_L3D6 |
| 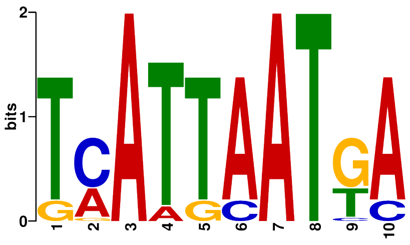 | DECOD_Motif4_10.2 | 1.43E-08 | L4_L3 |

| Motif logo | Motif Name | Motif p_value | Discovery stage |
|---|---|---|---|
| 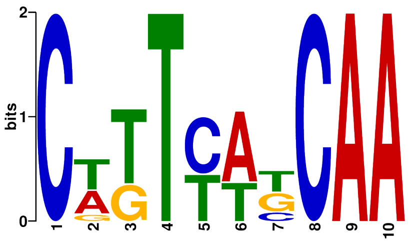 | DME_CDKTYWBCAA | 2.30E-08 | L3D9_L3D6 |
| 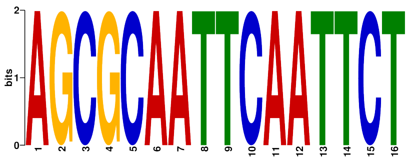 | DME_AGCGCAATTCAATTCT | 3.02E-08 | L3D9_L3 |
| 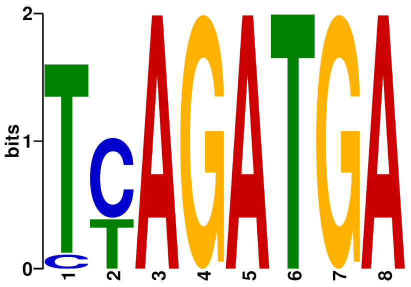 | DME_YYAGATGA | 3.19E-08 | L4_L3 |
| 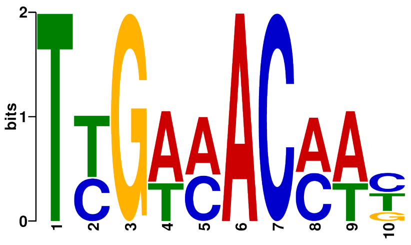 | DME_TYGWMACMWB | 3.74E-08 | L3_L3D6 |
| 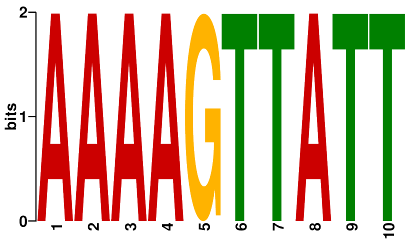 | DME_AAAAGTTATT | 3.74E-08 | L3D6_L3 |
| 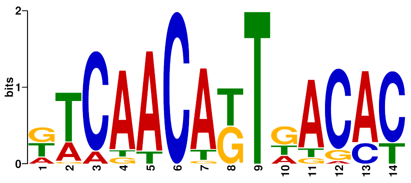 | gimme_94_MDmodule_Motif.14.9 | 3.85E-08 | L3_L3D9 |
| 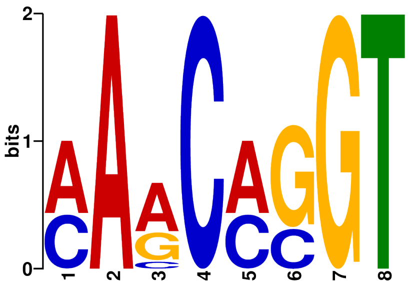 | DME_MAVCMSGT | 4.00E-08 | L4_L3D6 |
| 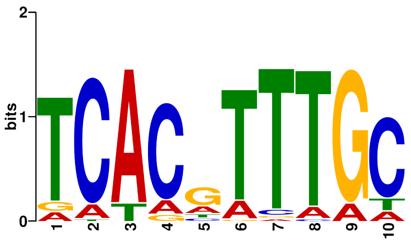 | gimme_65_MDmodule_Motif.10.3 | 4.66E-08 | L3_L4 |
| 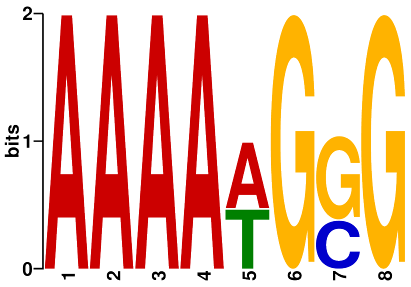 | DME_AAAAWGSG | 4.79E-08 | L3D6_L3D9 |

| Motif logo | Motif Name | Motif p_value | Discovery stage |
|---|---|---|---|
| 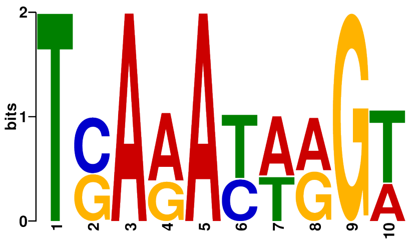 | DME_TSARAYWRGW | 5.04E-08 | L3D6_L4 |
| 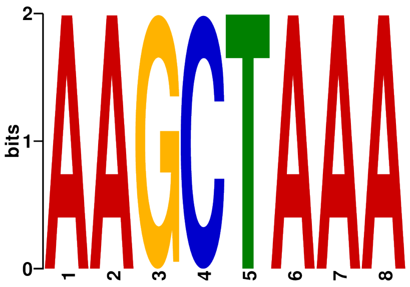 | DME_AAGCTAAA | 5.70E-08 | L3D9_L3 |
| 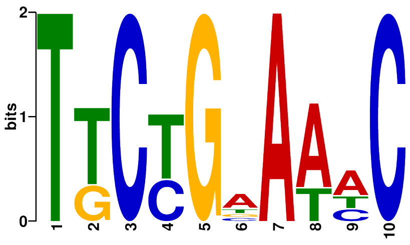 | DME_TKCYGVAWHC | 5.71E-08 | L3_L3D6 |
| 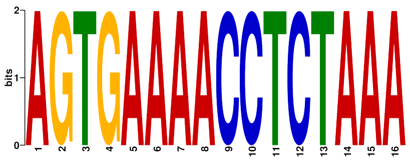 | DME_AGTGAAAACCTCTAAA | 5.81E-08 | L3D9_L3D6 |
| 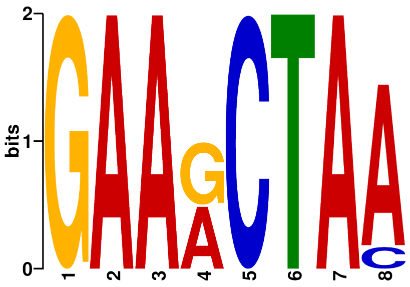 | DME_GAARCTAM | 6.62E-08 | L3_L3D6 |
| 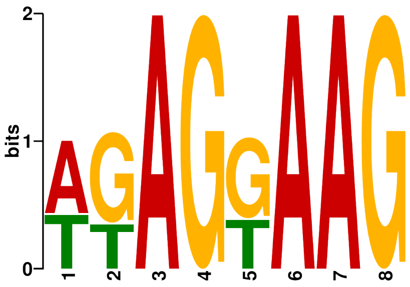 | DME_WKAGKAAG | 7.67E-08 | L4_L3D6 |
| 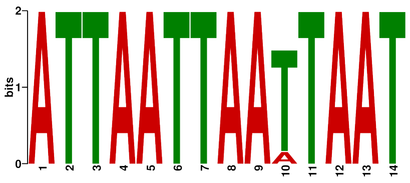 | DME_ATTAATTAAWTAAT | 9.56E-08 | L3D9_L3D6 |
| 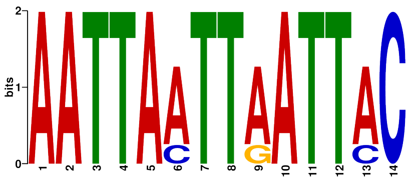 | DME_AATTAMTTRATTMC | 1.13E-07 | L4_L3 |
| 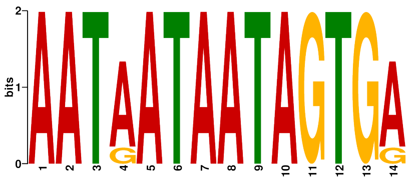 | DME_AATRATAATAGTGR | 1.20E-07 | L3D6_L4 |

| Motif logo | Motif Name | Motif p_value | Discovery stage |
|---|---|---|---|
| 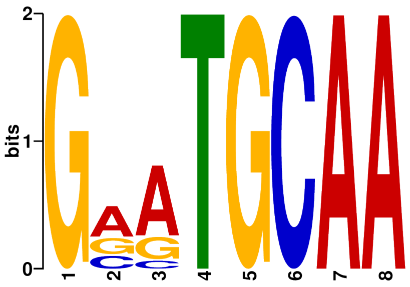 | DME_GVVTGCAA | 1.33E-07 | L4_L3 |
| 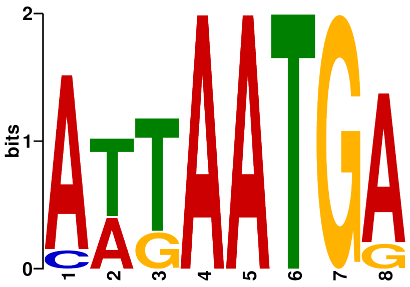 | DECOD_Motif3_8 | 1.41E-07 | L4_L3 |
| 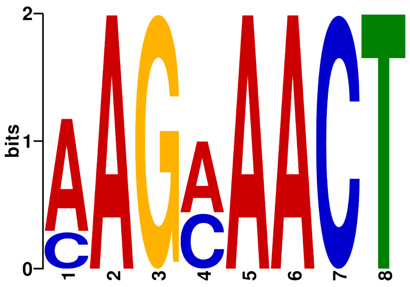 | DME_MAGMAACT | 1.67E-07 | L4_L3D6 |
| 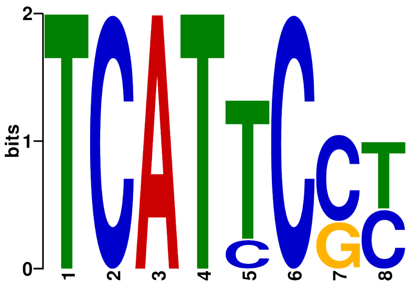 | DME_TCATTCSY | 1.69E-07 | L4_L3D6 |
| 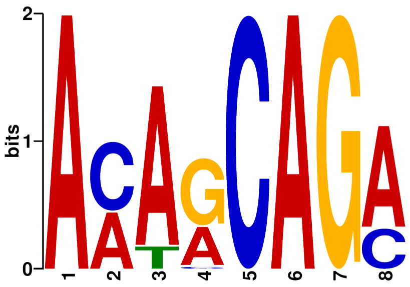 | DME_AMARCAGM | 1.81E-07 | L4_L3D6 |
| 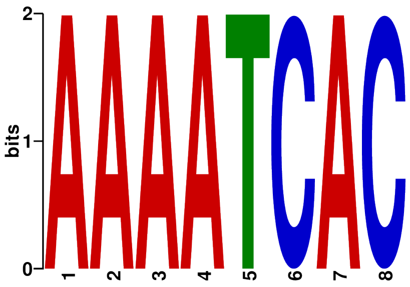 | DME_AAAATCAC | 1.88E-07 | L3_L3D6 |
| 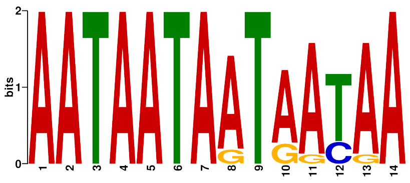 | DME_AATAATARTRRYRA | 1.96E-07 | L3D6_L3 |
| 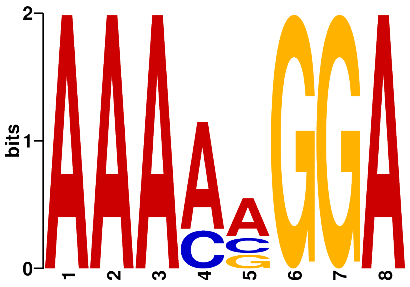 | DME_AAAMVGGA | 1.99E-07 | L3D6_L3 |

| Motif logo | Motif Name | Motif p_value | Discovery stage |
|---|---|---|---|
| 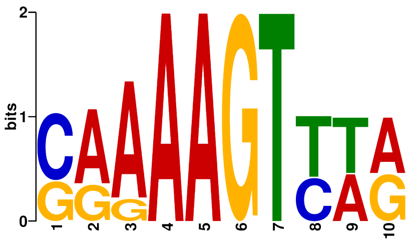 | DME_SRRAAGTYWR | 2.03E-07 | L3_L3D9 |
| 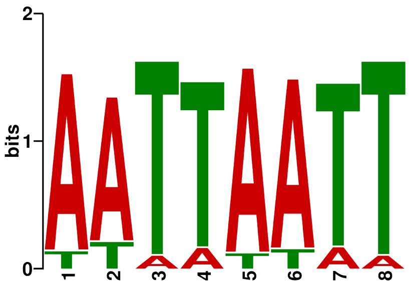 | gimme_169_Weeder_5 | 2.08E-07 | L4_L3 |
| 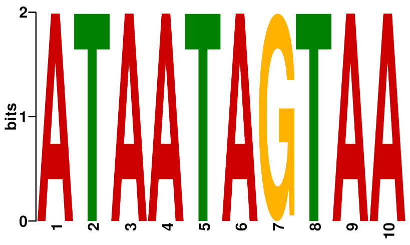 | DME_ATAATAGTAA | 2.15E-07 | L3D9_L3D6 |
| 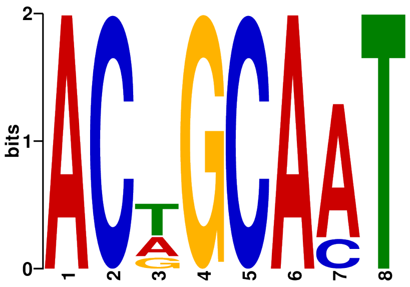 | DME_ACDGCAMT | 2.63E-07 | L4_L3 |
| 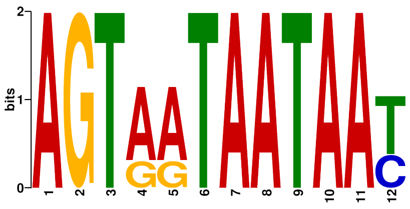 | DME_AGTRRTAATAAY | 2.67E-07 | L3D6_L4 |
| 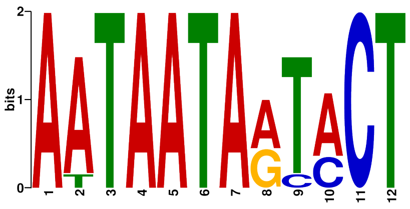 | DME_AWTAATARYMCT | 2.95E-07 | L3D6_L4 |
| 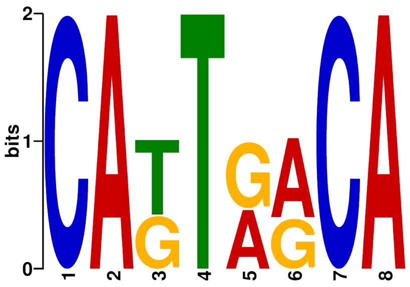 | DME_CAKTRRCA | 2.97E-07 | L3_L3D9 |
| 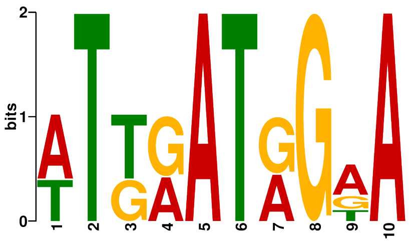 | DME_WTKRATRGDA | 3.48E-07 | L3D6_L3D9 |

| Motif logo | Motif Name | Motif p_value | Discovery stage |
|---|---|---|---|
| 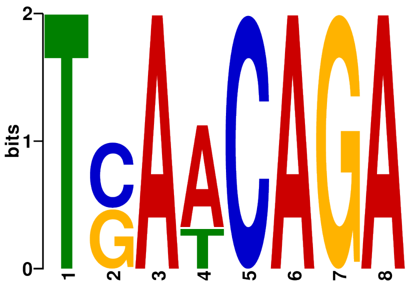 | DME_TSAWCAGA | 3.65E-07 | L3_L3D9 |
| 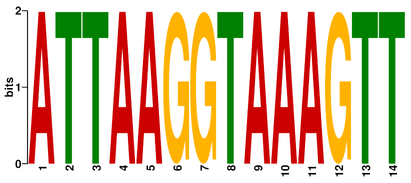 | DME_ATTAAGGTAAAGTT | 4.29E-07 | L4_L3 |
| 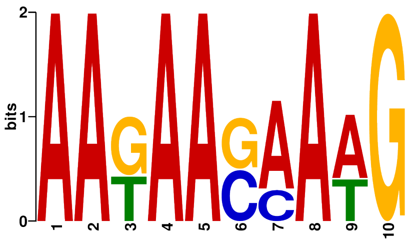 | DME_AAKAASMAWG | 4.43E-07 | L3D6_L3 |
| 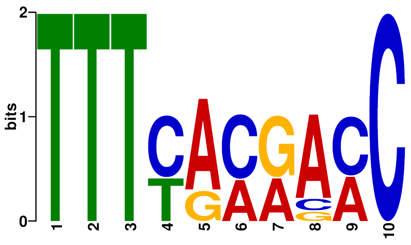 | DME_TTTYRMRVMC | 4.55E-07 | L3_L3D6 |
| 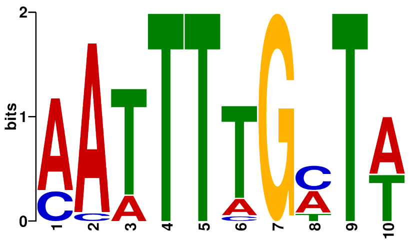 | DECOD_Motif4_10.1 | 4.94E-07 | L3D9_L3 |
| 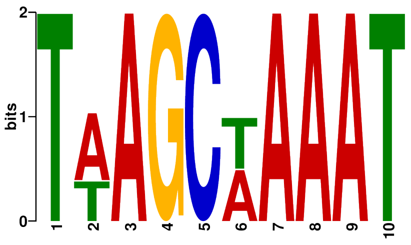 | DME_TWAGCWAAAT | 5.21E-07 | L3D9_L3 |
| 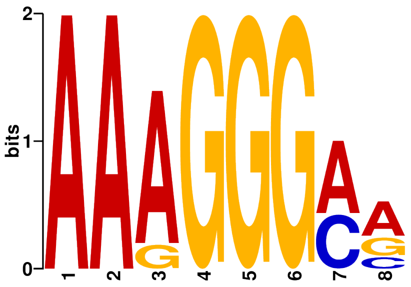 | DME_AARGGGMV | 5.40E-07 | L4_L3D6 |
| 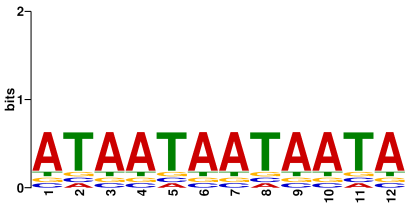 | gimme_44_Homer_12_1 | 7.61E-07 | L3D6_L3 |
| 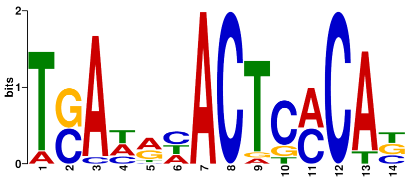 | gimme_160_MEME_8_w14 | 7.69E-07 | L3_L4 |

| Motif logo | Motif Name | Motif p_value | Discovery stage |
|---|---|---|---|
| 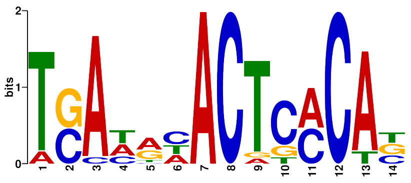 | gimme_120_MEME_8_w14 | 7.87E-07 | L3_L4 |
| 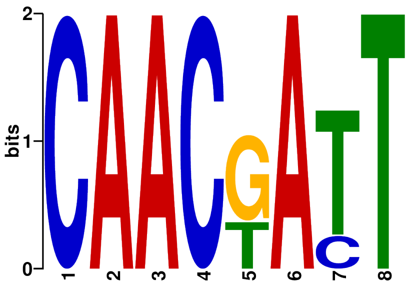 | DME_CAACKAYT | 8.51E-07 | L3D6_L3 |
| 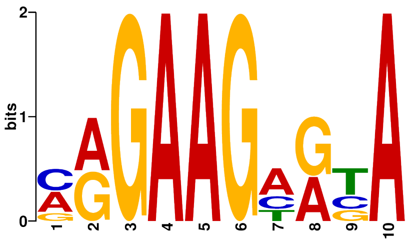 | DME_VRGAAGHRBA | 8.65E-07 | L3_L3D6 |
| 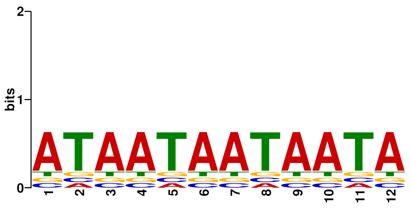 | gimme_32_Homer_12_1 | 8.70E-07 | L3D6_L3 |
| 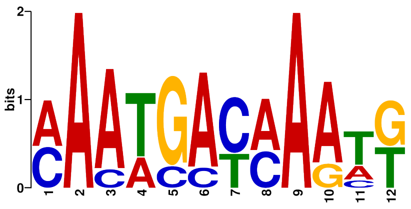 | DME_CAMWSMYMARHK | 8.93E-07 | L3D9_L3D6 |
| 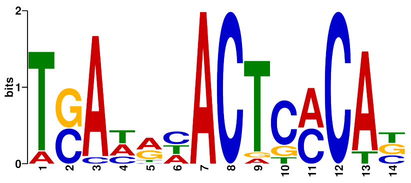 | gimme_140_MEME_8_w14 | 9.07E-07 | L3_L4 |
| 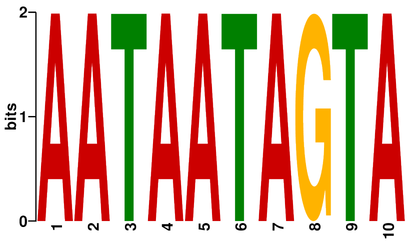 | DME_AATAATAGTA | 9.64E-07 | L3D6_L4 |
| 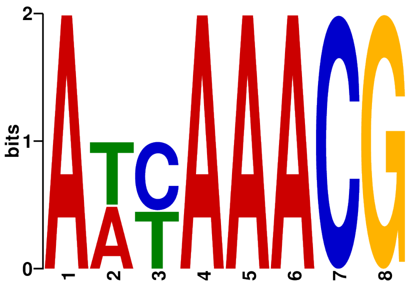 | DME_AWYAAACG | 9.87E-07 | L3_L4 |
| 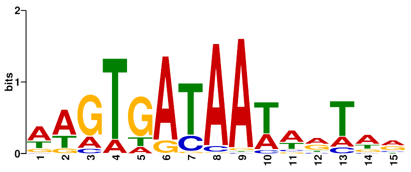 | gimme_103_Improbizer_AAGTGATAATAATAA | 9.92E-07 | L3D6_L3 |

| Motif logo | Motif Name | Motif p_value | Discovery stage |
|---|---|---|---|
| 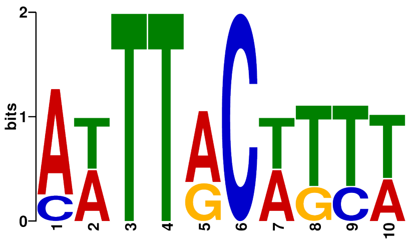 | DECOD_Motif10_10.1 | 1.03E-06 | L3D9_L3D6 |
| 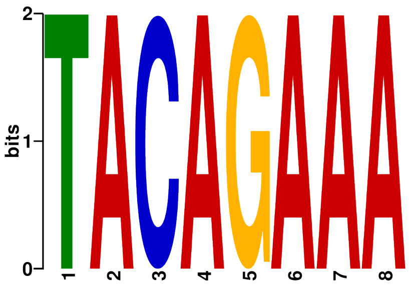 | DME_TACAGAAA | 1.06E-06 | L4_L3D6 |
| 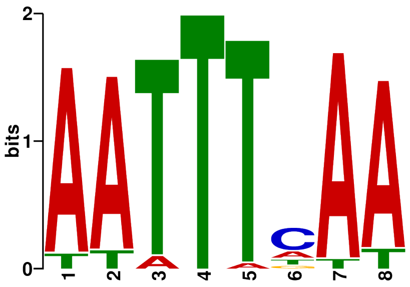 | gimme_171_Weeder_9 | 1.06E-06 | L3D9_L3D6 |
| 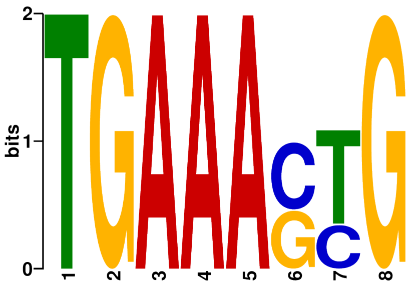 | DME_TGAAASYG | 1.09E-06 | L3D9_L3D6 |
| 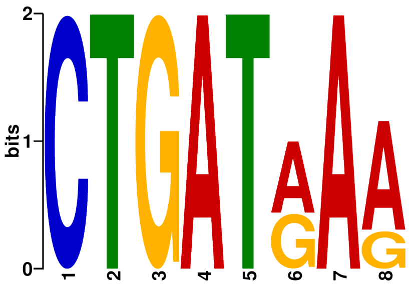 | DME_CTGATRAR | 1.10E-06 | L3D6_L3D9 |
| 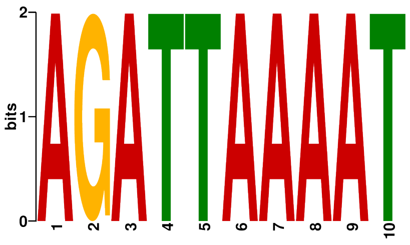 | DME_AGATTAAAAT | 1.12E-06 | L3D6_L3 |
| 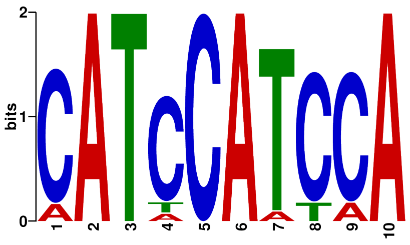 | DME_MATHCAWYMA | 1.16E-06 | L3D6_L3D9 |
| 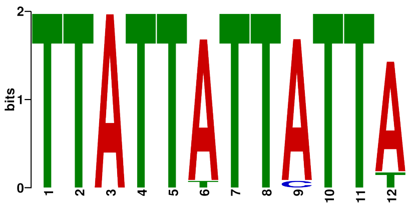 | gimme_20_BioProspector_w12_2 | 1.18E-06 | L3_L3D9 |

| Motif logo | Motif Name | Motif p_value | Discovery stage |
|---|---|---|---|
| 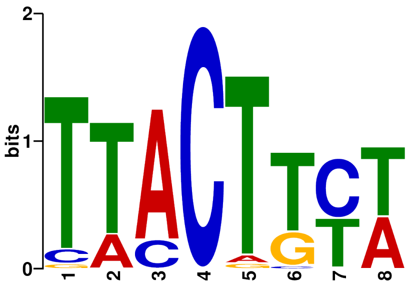 | gimme_60_Improbizer_TTACTTCT | 1.19E-06 | L3D9_L3D6 |
| 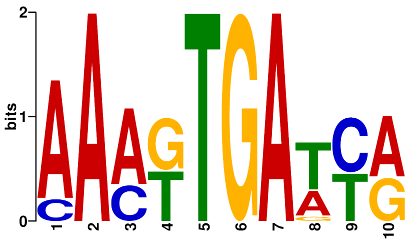 | DME_MAMKTGADYR | 1.25E-06 | L3_L3D6 |
| 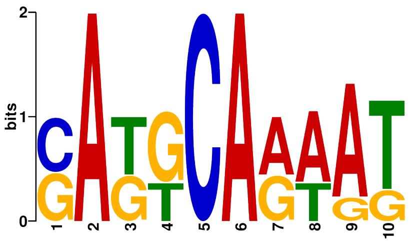 | DME_SAKKCARWRK | 1.28E-06 | L3_L3D6 |
| 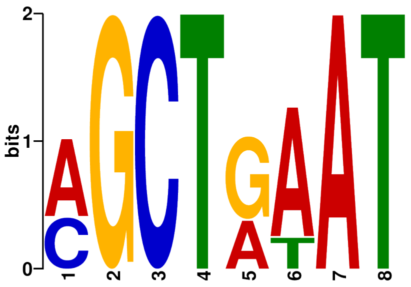 | DME_MGCTRWAT | 1.36E-06 | L3_L3D6 |
| 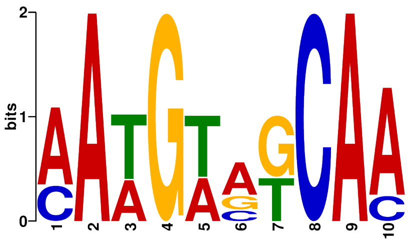 | DME_MAWGWVKCAM | 1.43E-06 | L3D6_L4 |
| 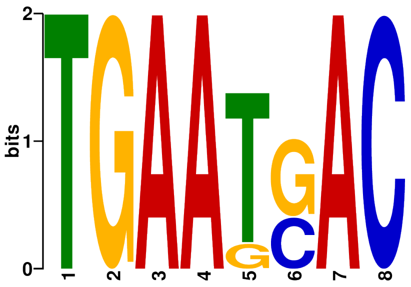 | DME_TGAAKSAC | 1.44E-06 | L3_L3D6 |
| 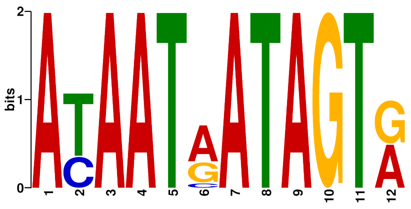 | DME_AYAATVATAGTR | 1.46E-06 | L3D6_L4 |
| 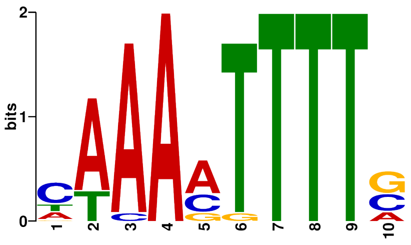 | DECOD_Motif10_10.2 | 1.60E-06 | L3D9_L3 |

| Motif logo | Motif Name | Motif p_value | Discovery stage |
|---|---|---|---|
| 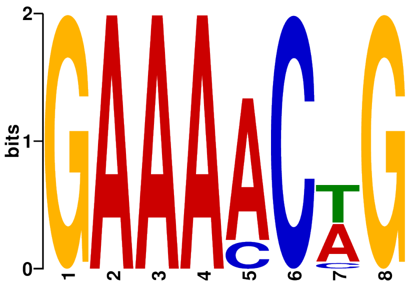 | DME_GAAAMCHG | 1.63E-06 | L3_L3D6 |
| 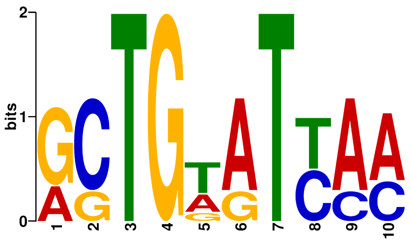 | DME_GSTGDRTYMM | 1.70E-06 | L3_L3D6 |
| 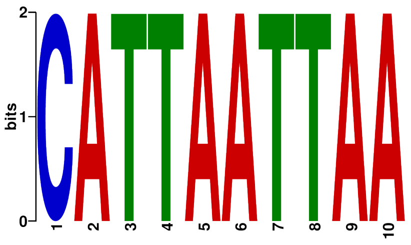 | DME_CATTAATTAA | 1.73E-06 | L4_L3 |
| 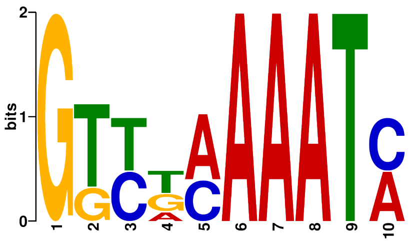 | DME_GKYDMAAATM | 1.80E-06 | L3D6_L3D9 |
| 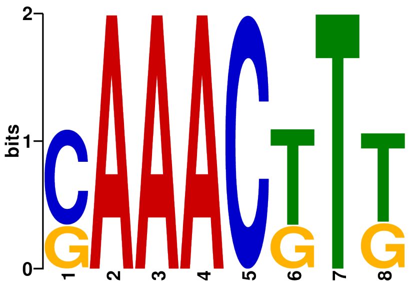 | DME_SAAACKTK | 1.84E-06 | L3_L4 |
| 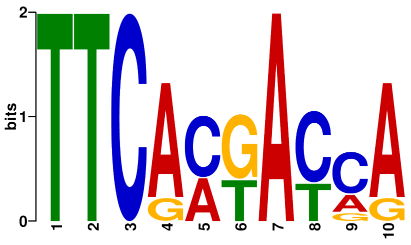 | DME_TTCRMKAYVR | 1.92E-06 | L3_L3D6 |
| 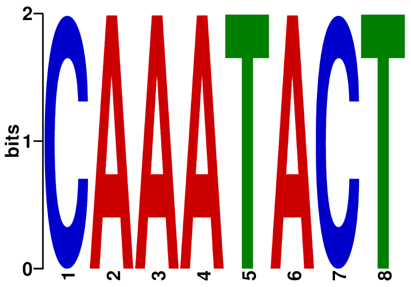 | DME_CAAATACT | 1.92E-06 | L3_L4 |
| 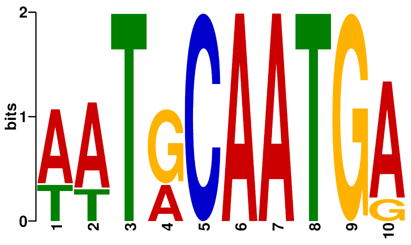 | DME_WWTRCAATGR | 1.99E-06 | L3D9_L3D6 |

| Motif logo | Motif Name | Motif p_value | Discovery stage |
|---|---|---|---|
| 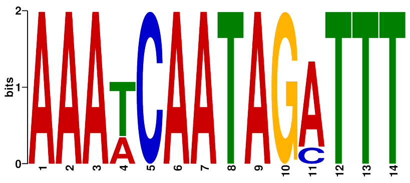 | DME_AAAWCAATAGMTTT | 2.00E-06 | L3D6_L3 |
| 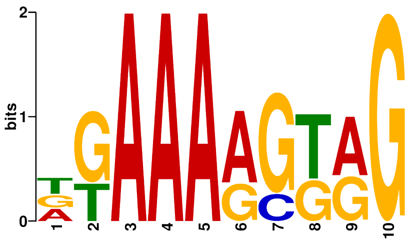 | DME_DKAAARSKRG | 2.18E-06 | L4_L3 |
| 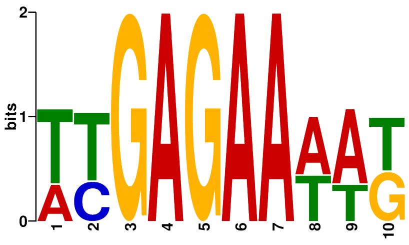 | DME_WYGAGAAWWK | 2.26E-06 | L3_L4 |
| 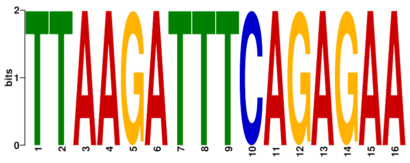 | DME_TTAAGATTTCAGAGAA | 2.36E-06 | L3D6_L3D9 |
| 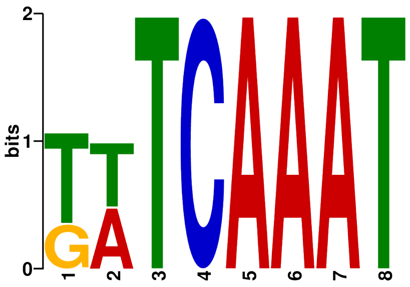 | gimme_12_BioProspector_w8_1 | 2.43E-06 | L3D6_L3D9 |
| 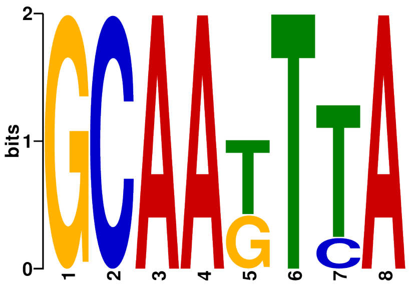 | DME_GCAAKTYA | 2.44E-06 | L3D9_L3D6 |
| 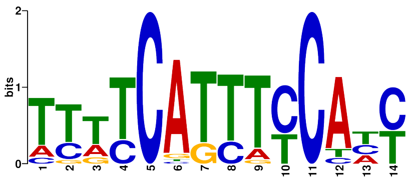 | gimme_159_MEME_10_w14 | 2.53E-06 | L3D6_L3 |
| 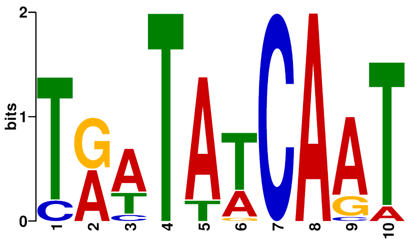 | DECOD_Motif8_10.1 | 2.85E-06 | L3D6_L3D9 |
| 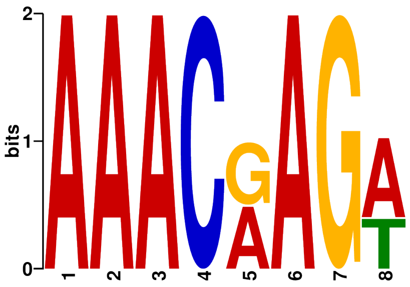 | DME_AAACRAGW | 3.05E-06 | L3_L4 |

| Motif logo | Motif Name | Motif p_value | Discovery stage |
|---|---|---|---|
| 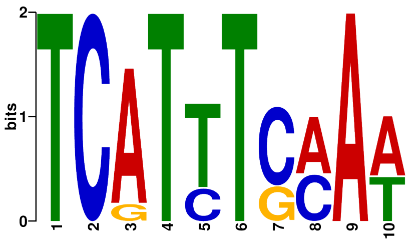 | DME_TCRTYTSMAW | 3.06E-06 | L3D6_L3 |
| 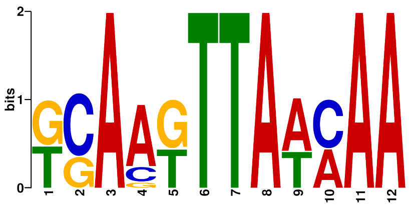 | DME_KSAVKTTAWMAA | 3.23E-06 | L3D9_L3D6 |
| 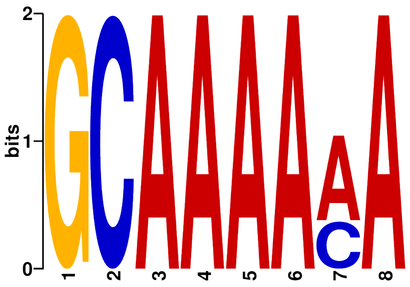 | DME_GCAAAAMA | 3.52E-06 | L3_L4 |
| 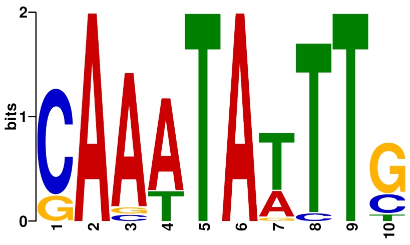 | DECOD_Motif8_10.2 | 3.63E-06 | L3D9_L3 |
| 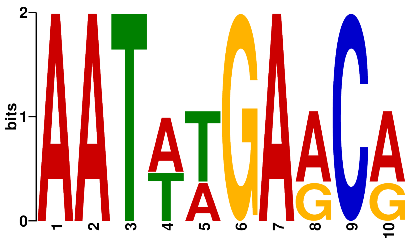 | DME_AATWWGARCR | 3.69E-06 | L3_L3D9 |
| 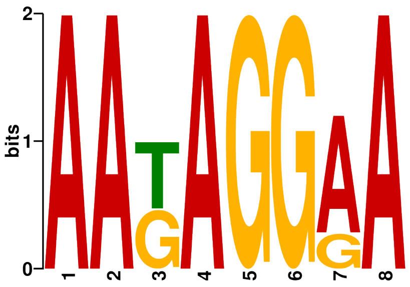 | DME_AAKAGGRA | 4.03E-06 | L4_L3D6 |
| 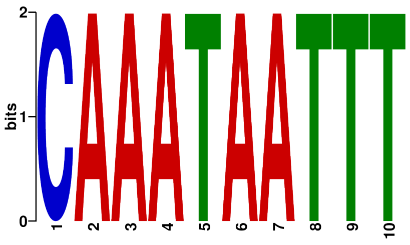 | DME_CAAATAATTT | 4.27E-06 | L3D9_L3D6 |
| 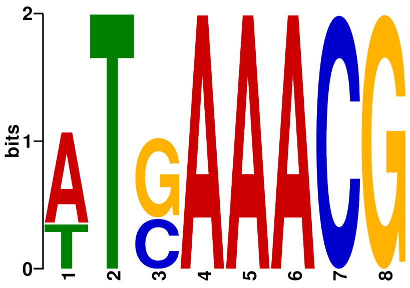 | DME_WTSAAACG | 4.40E-06 | L3_L4 |

| Motif logo | Motif Name | Motif p_value | Discovery stage |
|---|---|---|---|
| 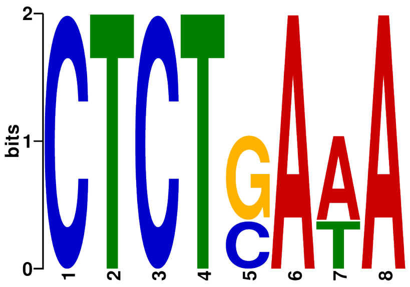 | DME_CTCTSAWA | 4.43E-06 | L3D6_L4 |
| 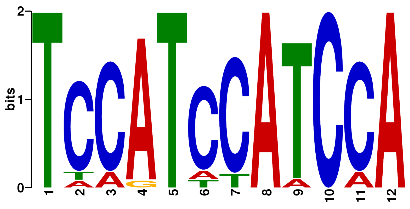 | DME_THMRTHYAWCMA | 4.63E-06 | L3D6_L3 |
| 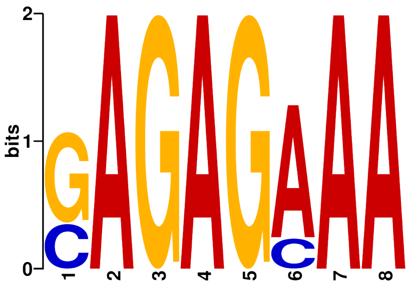 | DME_SAGAGMAA | 4.75E-06 | L3D6_L3D9 |
| 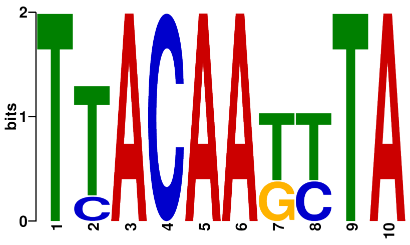 | DME_TYACAAKYTA | 4.77E-06 | L3D6_L3D9 |
| 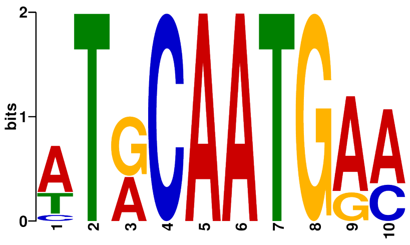 | DME_HTRCAATGRM | 4.83E-06 | L3D9_L3D6 |
| 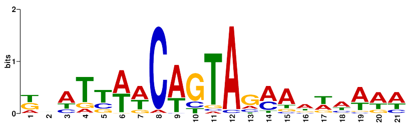 | gimme_53_Improbizer_TTATTAACAGTAGAAATAAAA | 5.52E-06 | L3_L4 |
| 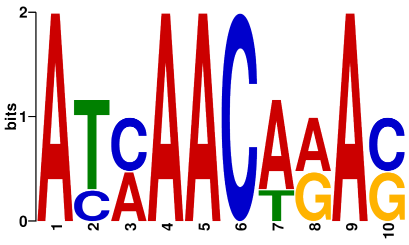 | DME_AYMAACWRAS | 5.95E-06 | L3_L3D9 |
| 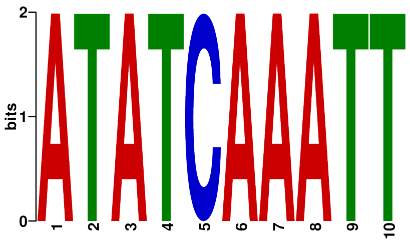 | DME_ATATCAAATT | 6.47E-06 | L3D9_L3 |
| 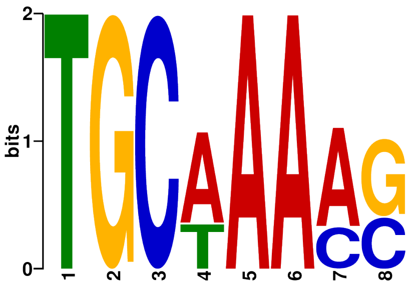 | DME_TGCWAAMS | 6.51E-06 | L3_L3D9 |

| Motif logo | Motif Name | Motif p_value | Discovery stage |
|---|---|---|---|
| 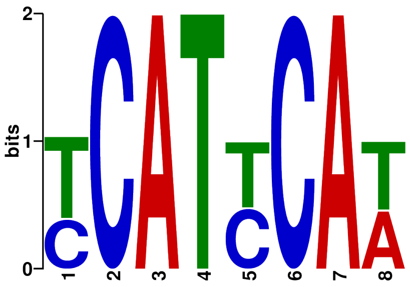 | DME_YCATYCAW | 6.68E-06 | L3_L3D9 |
| 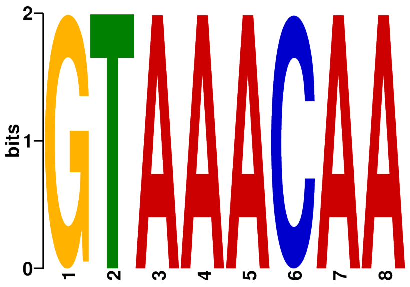 | DME_GTAAACAA | 6.71E-06 | L3_L3D6 |
| 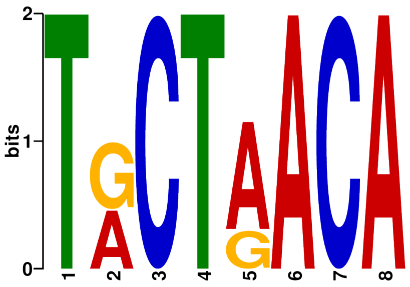 | DME_TRCTRACA | 6.76E-06 | L4_L3D6 |
| 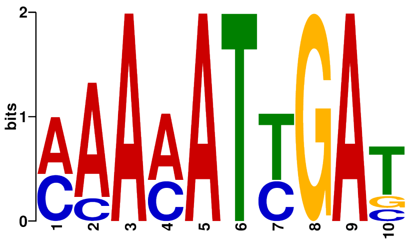 | DME_MMAMATYGAB | 6.77E-06 | L3_L4 |
| 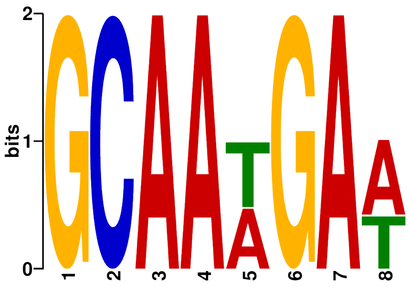 | DME_GCAAWGAW | 7.30E-06 | L3D9_L3D6 |
| 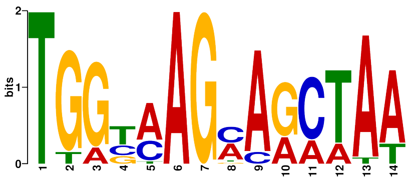 | gimme_163_MEME_8_w14 | 7.32E-06 | L3_L3D6 |
| 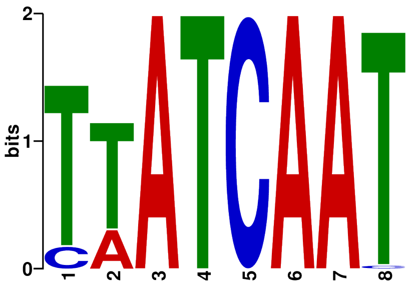 | gimme_20_BioProspector_w8_4 | 7.45E-06 | L3D9_L3 |
| 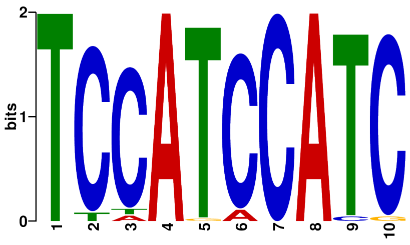 | DME_TYHAKMCAYS | 7.57E-06 | L3D6_L4 |

| Motif logo | Motif Name | Motif p_value | Discovery stage |
|---|---|---|---|
| 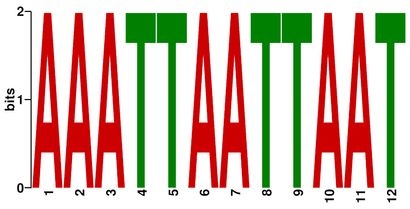 | DME_AAATTAATTAAT | 7.58E-06 | L3D6_L3 |
| 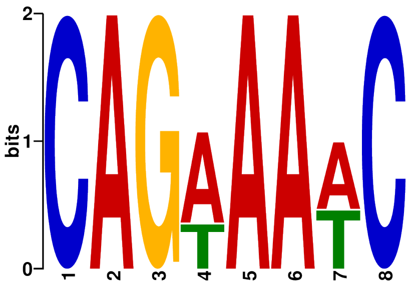 | DME_CAGWAAWC | 7.59E-06 | L4_L3D6 |
| 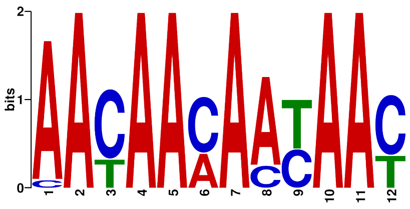 | DME_MAYAAMAMYAAY | 7.67E-06 | L3D6_L4 |
| 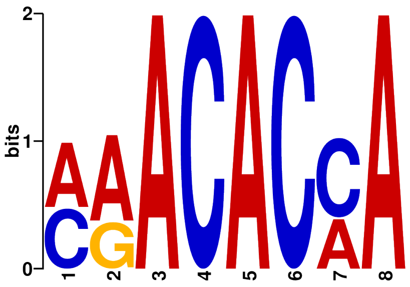 | DME_MAACACMA | 7.72E-06 | L3_L3D6 |
| 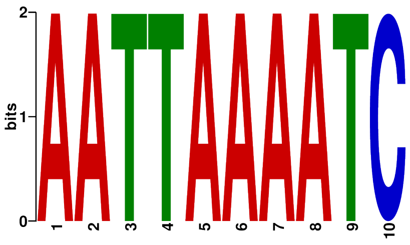 | DME_AATTAAAATC | 7.84E-06 | L3_L3D6 |
| 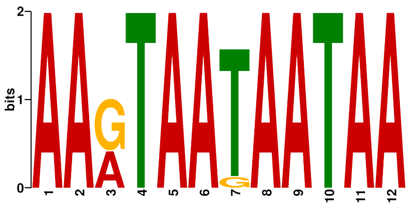 | DME_AARTAAKAATAA | 8.16E-06 | L3D6_L3 |
| 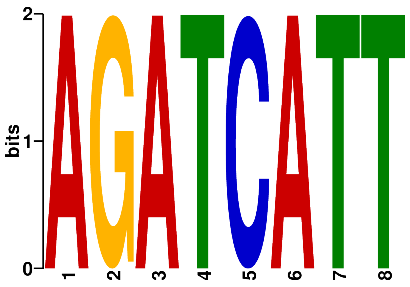 | DME_AGATCATT | 8.45E-06 | L3_L4 |
| 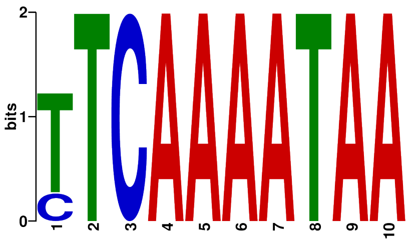 | DME_YTCAAAATAA | 8.68E-06 | L3_L4 |

| Motif logo | Motif Name | Motif p_value | Discovery stage |
|---|---|---|---|
| 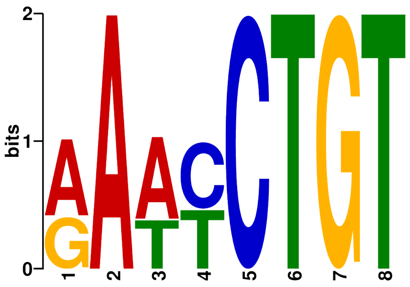 | DME_RAWYCTGT | 8.77E-06 | L3D6_L4 |
| 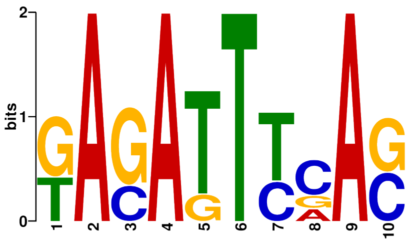 | DME_KASAKTYVAS | 8.87E-06 | L3_L4 |
| 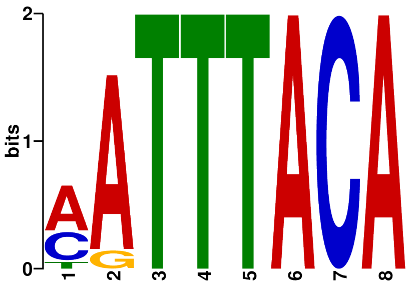 | DECOD_Motif7_8 | 9.07E-06 | L4_L3 |
| 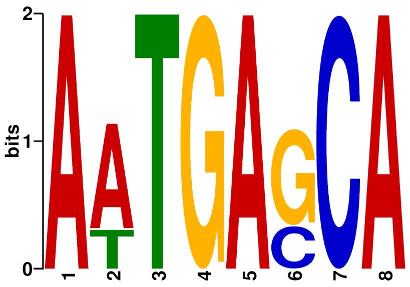 | DME_AWTGASCA | 9.33E-06 | L3D6_L3 |
| 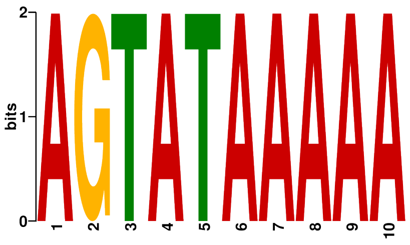 | DME_AGTATAAAAA | 9.45E-06 | L3D6_L4 |
| 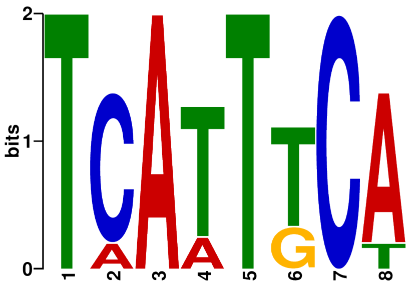 | DECOD_Motif1_8 | 9.46E-06 | L3D9_L3D6 |
| 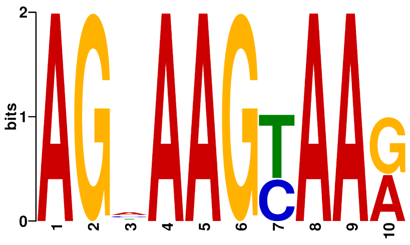 | DME_AGNAAGYAAR | 9.47E-06 | L3D9_L3D6 |
| 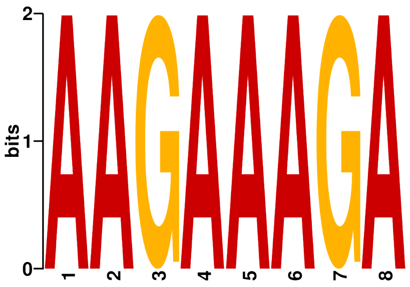 | DME_AAGAAAGA | 9.51E-06 | L4_L3 |

| Motif logo | Motif Name | Motif p_value | Discovery stage |
|---|---|---|---|
| 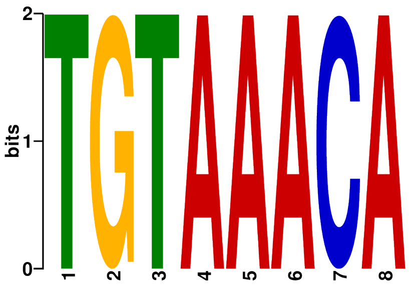 | DME_TGTAAACA | 9.91E-06 | L3_L3D6 |
| 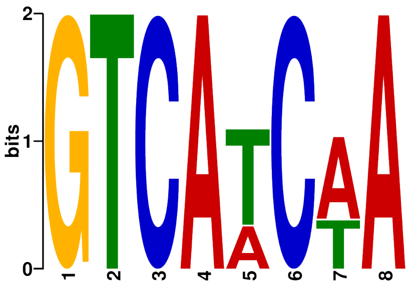 | DME_GTCAWCWA | 1.01E-05 | L4_L3D6 |
| 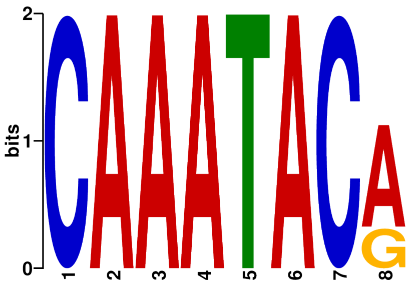 | DME_CAAATACR | 1.04E-05 | L3_L4 |
| 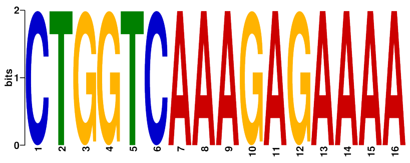 | DME_CTGGTCAAAGAGAAAA.1 | 1.04E-05 | L3D9_L3D6 |
| 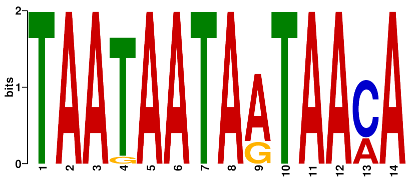 | DME_TAAKAMTAVTAAMA | 1.06E-05 | L3D6_L4 |
| 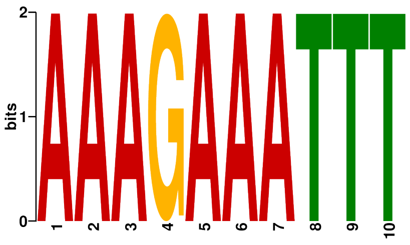 | DME_AAAGAAATTT | 1.07E-05 | L3D6_L4 |
| 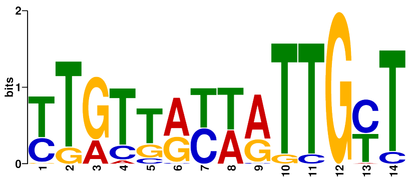 | gimme_135_MEME_10_w14 | 1.09E-05 | L3D6_L4 |
| 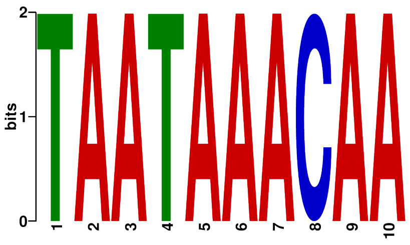 | DME_TAATAAACAA | 1.10E-05 | L3D6_L4 |
| 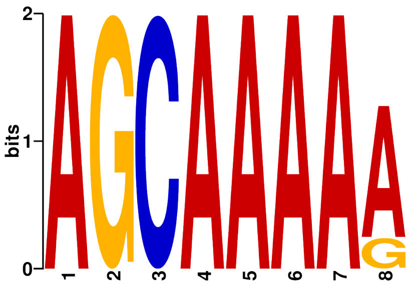 | DME_AGCAAAAR | 1.14E-05 | L3_L4 |

| Motif logo | Motif Name | Motif p_value | Discovery stage |
|---|---|---|---|
| 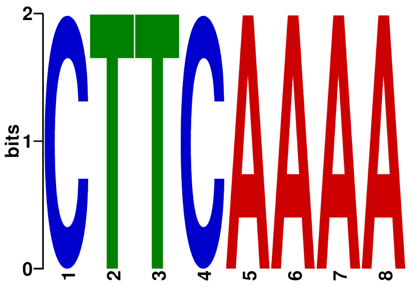 | DME_CTTCAAAA | 1.15E-05 | L3D6_L3 |
| 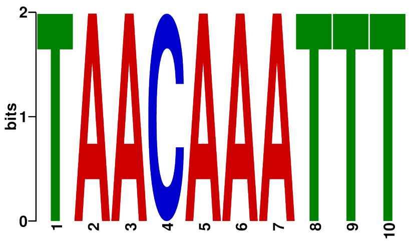 | DME_TAACAAATTT | 1.17E-05 | L3D6_L3 |
| 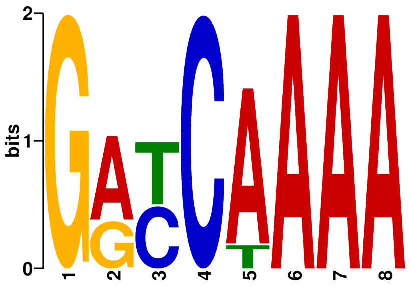 | DME_GRYCWAAA | 1.26E-05 | L3_L4 |
| 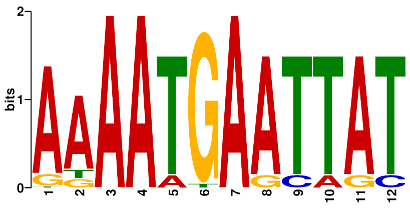 | gimme_39_Homer_12_1 | 1.28E-05 | L3_L3D9 |
| 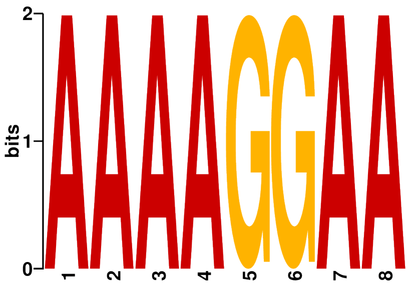 | DME_AAAAGGAA | 1.30E-05 | L3D6_L3 |
| 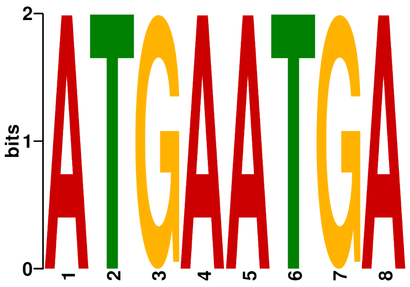 | DME_ATGAATGA | 1.32E-05 | L3D6_L4 |
| 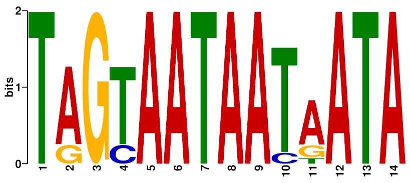 | DME_TRGYAATAAYDATA | 1.43E-05 | L3D6_L3 |
| 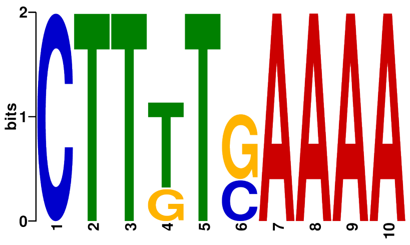 | DME_CTTKTSAAAA | 1.43E-05 | L3D9_L3 |

| Motif logo | Motif Name | Motif p_value | Discovery stage |
|---|---|---|---|
| 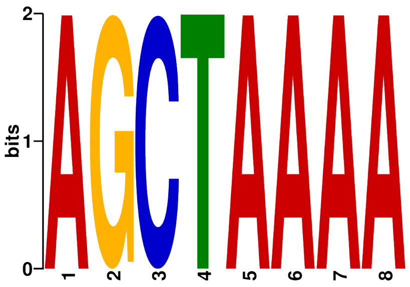 | DME_AGCTAAAA | 1.47E-05 | L3D6_L3D9 |
| 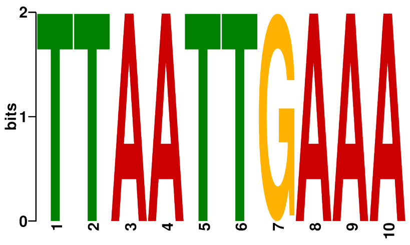 | DME_TTAATTGAAA | 1.51E-05 | L3D9_L3D6 |
| 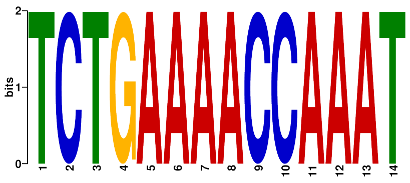 | DME_TCTGAAAACCAAAT | 1.63E-05 | L3_L3D9 |
| 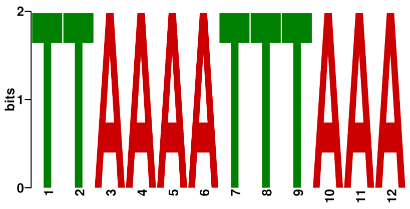 | DME_TTAAAATTTAAA | 1.72E-05 | L3D6_L4 |
| 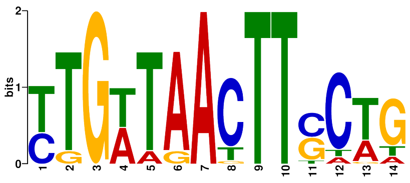 | gimme_72_MDmodule_Motif.14.10 | 1.76E-05 | L3D9_L3D6 |
| 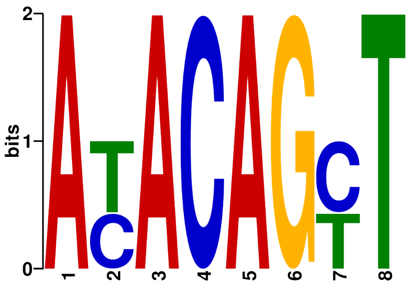 | DME_AYACAGYT | 1.79E-05 | L3_L4 |
| 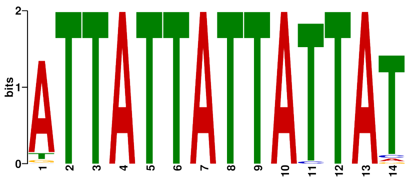 | gimme_96_MDmodule_Motif.14.1 | 1.82E-05 | L3_L3D9 |
| 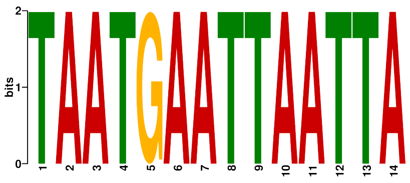 | DME_TAATGAATTAATTA | 1.83E-05 | L3D9_L3D6 |
| 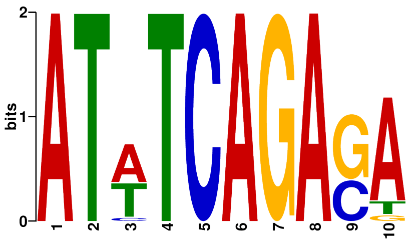 | DME_ATHTCAGASD | 1.86E-05 | L3D6_L4 |

| Motif logo | Motif Name | Motif p_value | Discovery stage |
|---|---|---|---|
| 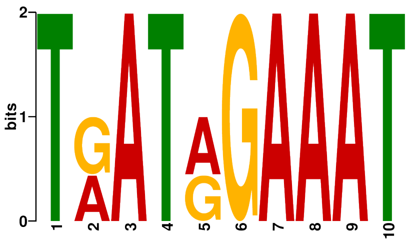 | DME_TRATRGAAAT | 1.89E-05 | L3D6_L4 |
| 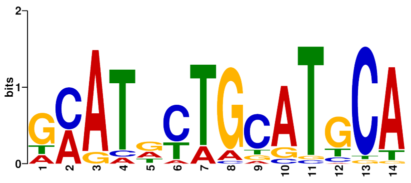 | gimme_114_MDmodule_Motif.14.10 | 1.94E-05 | L4_L3 |
| 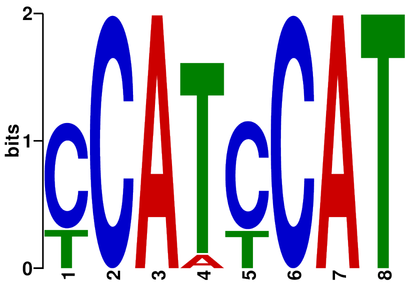 | DME_YCAWYCAT | 2.02E-05 | L3D6_L4 |
| 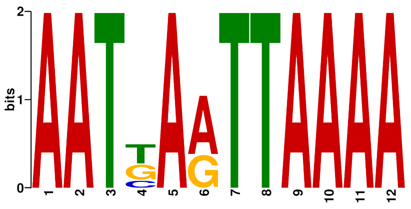 | DME_AATBARTTAAAA | 2.13E-05 | L3D9_L3 |
| 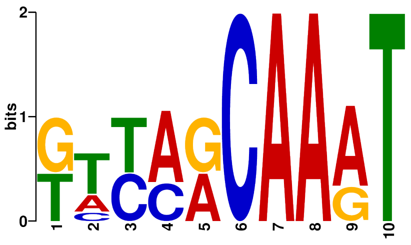 | DME_KHYMRCAART | 2.23E-05 | L3D9_L3D6 |
| 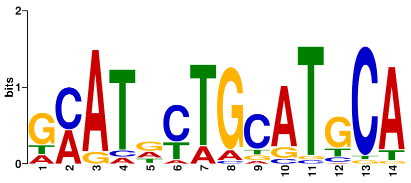 | gimme_84_MDmodule_Motif.14.10 | 2.27E-05 | L4_L3 |
| 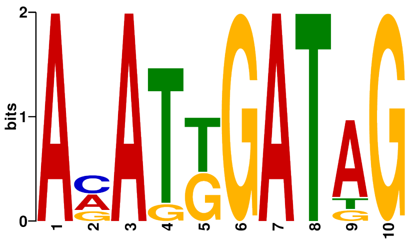 | DME_AVAKKGATDG | 2.45E-05 | L3D6_L3D9 |
| 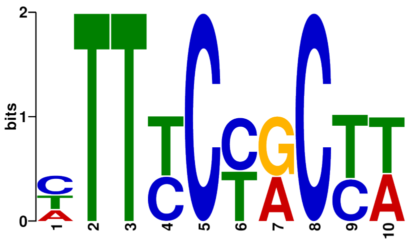 | DME_HTTYCYRCYW | 2.50E-05 | L3D9_L3D6 |
| 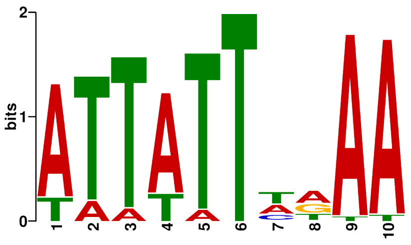 | gimme_165_Weeder_3 | 2.54E-05 | L3_L4 |

| Motif logo | Motif Name | Motif p_value | Discovery stage |
|---|---|---|---|
| 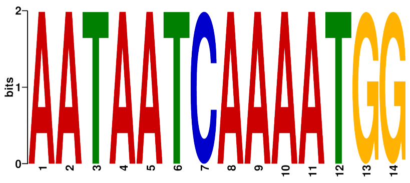 | DME_AATAATCAAAATGG | 2.55E-05 | L3_L3D9 |
| 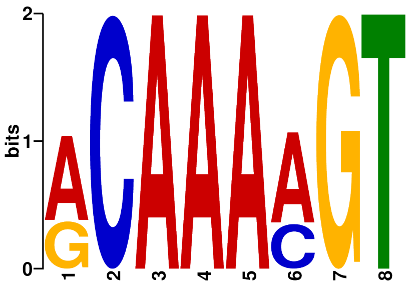 | DME_RCAAAMGT | 2.61E-05 | L3D9_L3D6 |
| 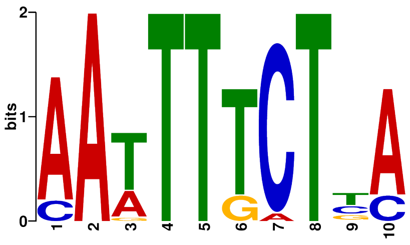 | DECOD_Motif3_10 | 2.70E-05 | L3_L4 |
| 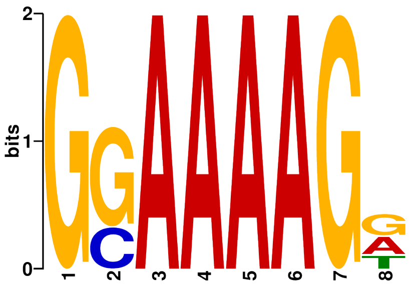 | DME_GSAAAAGD | 2.89E-05 | L4_L3 |
| 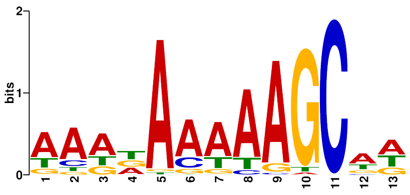 | gimme_60_Improbizer_AAATAAAAAGCAA | 2.89E-05 | L3_L4 |
| 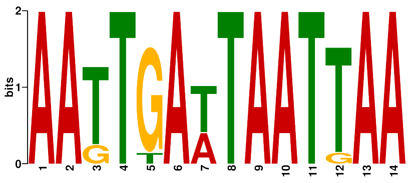 | DME_AAKTKAWTAATKAA | 2.91E-05 | L3D6_L4 |
| 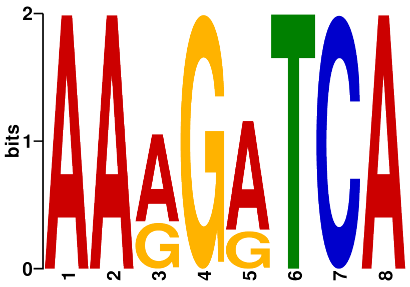 | DME_AARGRTCA | 3.04E-05 | L3_L3D6 |
| 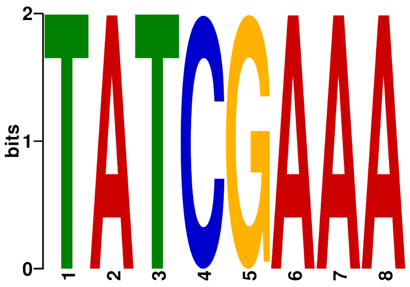 | DME_TATCGAAA | 3.10E-05 | L3D9_L3 |
| 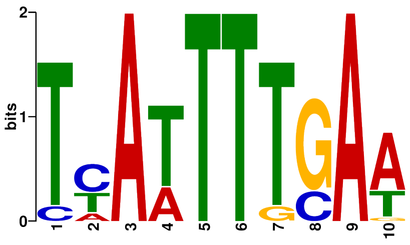 | DECOD_Motif1_10 | 3.51E-05 | L3_L3D9 |

| Motif logo | Motif Name | Motif p_value | Discovery stage |
|---|---|---|---|
| 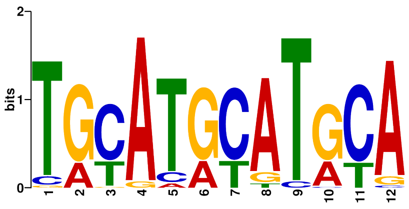 | gimme_103_MDmodule_Motif.12.1 | 3.51E-05 | L3_L4 |
| 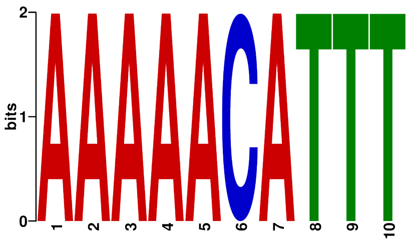 | DME_AAAAACATTT | 3.54E-05 | L3_L4 |
| 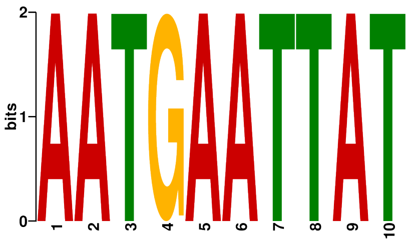 | DME_AATGAATTAT | 3.87E-05 | L3_L3D9 |
| 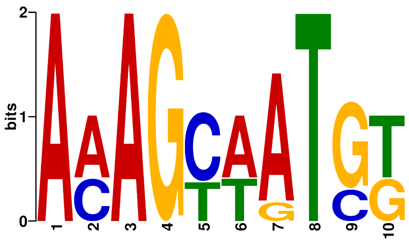 | DME_AMAGYWATSK | 3.89E-05 | L3_L4 |
| 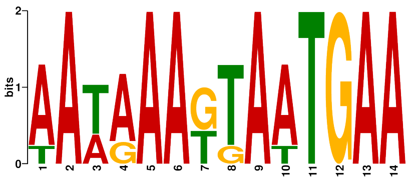 | DME_WAWRAAKKAWTGAA | 3.95E-05 | L3D9_L3D6 |
| 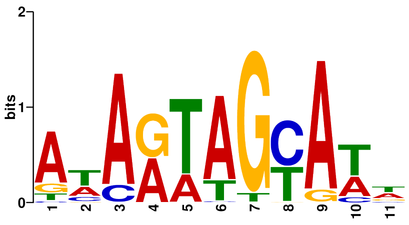 | gimme_60_Improbizer_ATAGTAGCATT | 4.03E-05 | L3_L3D9 |
| 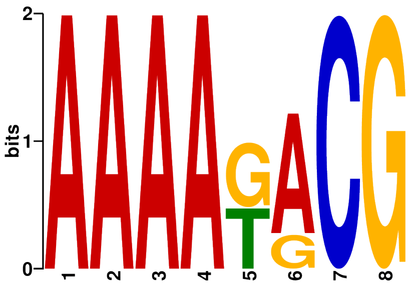 | DME_AAAAKRCG | 4.08E-05 | L3D6_L3 |
| 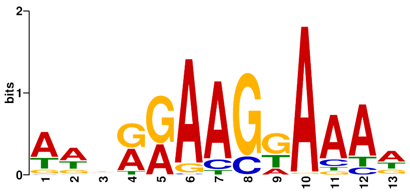 | gimme_56_Improbizer_AATGGAAGGAAAA | 4.32E-05 | L3D9_L3D6 |
| 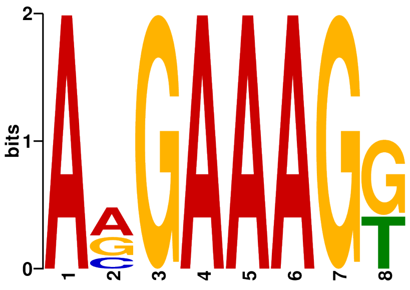 | DME_AVGAAAGK | 4.55E-05 | L3D9_L3D6 |

| Motif logo | Motif Name | Motif p_value | Discovery stage |
|---|---|---|---|
| 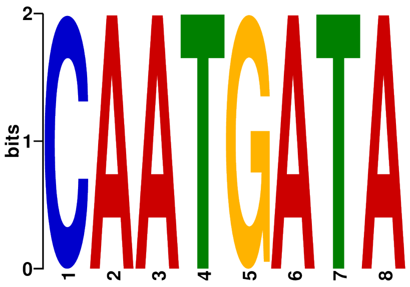 | DME_CAATGATA | 4.55E-05 | L3D9_L3D6 |
| 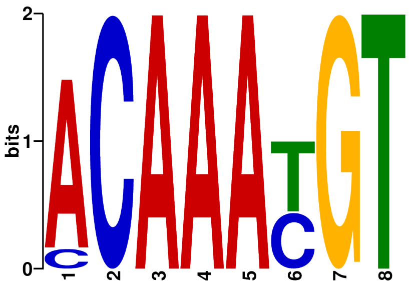 | DME_MCAAAYGT | 4.63E-05 | L3D9_L3 |
| 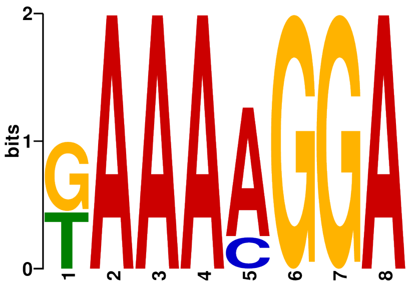 | DME_KAAAMGGA | 4.74E-05 | L3D6_L3D9 |
| 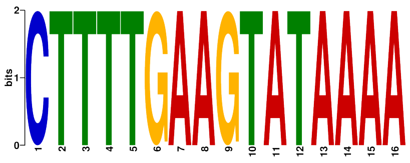 | DME_CTTTTGAAGTATAAAA | 4.83E-05 | L3D6_L4 |
| 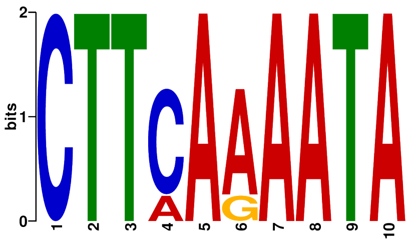 | DME_CTTMARAATA | 5.14E-05 | L3_L3D9 |
| 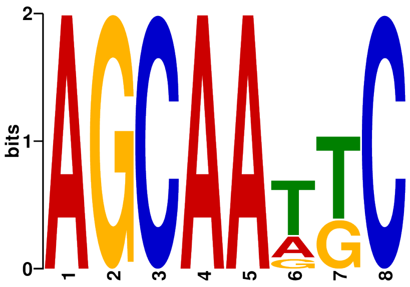 | DME_AGCAADKC | 5.17E-05 | L4_L3 |
| 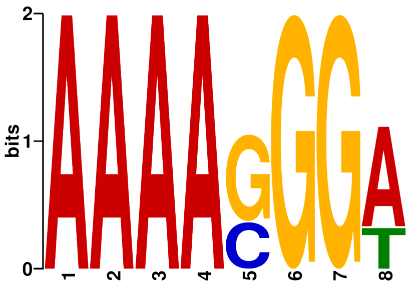 | DME_AAAASGGW | 5.39E-05 | L3D6_L3D9 |
| 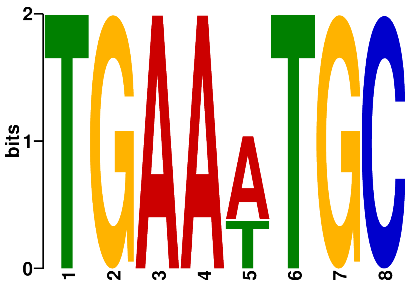 | DME_TGAAWTGC | 5.43E-05 | L4_L3 |

| Motif logo | Motif Name | Motif p_value | Discovery stage |
|---|---|---|---|
| 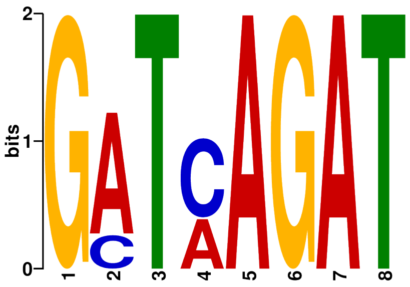 | DME_GMTMAGAT | 5.52E-05 | L3D6_L3D9 |
| 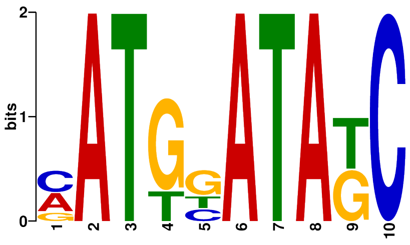 | DME_VATKBATAKC | 5.65E-05 | L3D6_L3D9 |
| 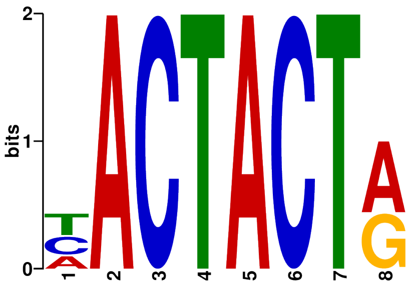 | DME_HACTACTR | 5.68E-05 | L3D9_L3 |
| 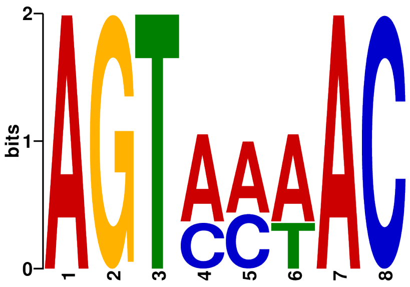 | DME_AGTMMWAC | 5.78E-05 | L3_L4 |
| 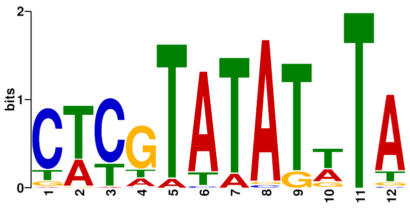 | gimme_79_MDmodule_Motif.12.7 | 6.03E-05 | L3_L4 |
| 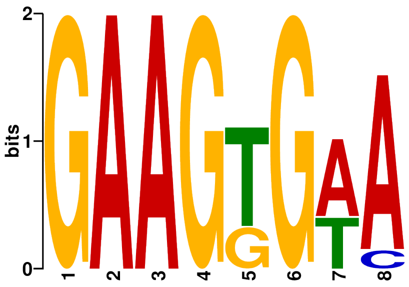 | DME_GAAGKGWM | 6.19E-05 | L4_L3D6 |
| 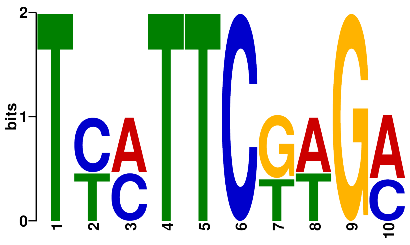 | DME_TYMTTCKWGM | 6.20E-05 | L4_L3 |
| 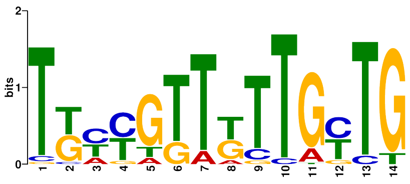 | gimme_154_MEME_5_w14 | 6.46E-05 | L3D6_L3 |

| Motif logo | Motif Name | Motif p_value | Discovery stage |
|---|---|---|---|
| 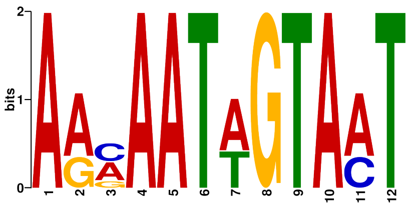 | DME_ARVAATWGTAMT | 6.73E-05 | L3D6_L3 |
| 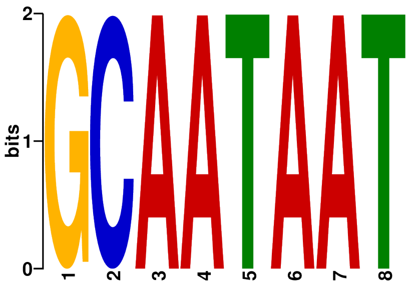 | DME_GCAATAAT | 6.91E-05 | L3D9_L3D6 |
| 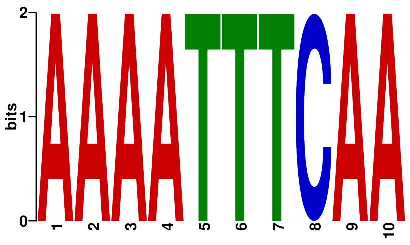 | DME_AAAATTTCAA | 7.07E-05 | L3D6_L4 |
| 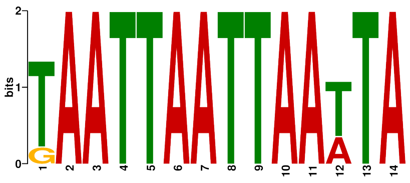 | DME_KAATTAATTAAWTA | 7.12E-05 | L3D9_L3 |
| 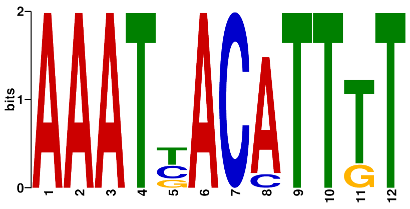 | DME_AAATBACMTTKT | 7.15E-05 | L3D9_L3 |
| 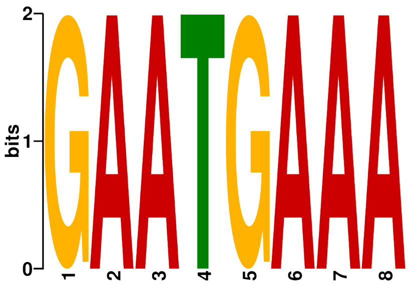 | DME_GAATGAAA | 7.58E-05 | L3D9_L3D6 |
| 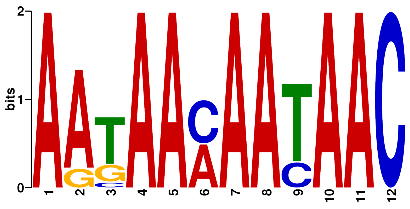 | DME_ARBAAMAAYAAC | 7.60E-05 | L3D6_L3D9 |
| 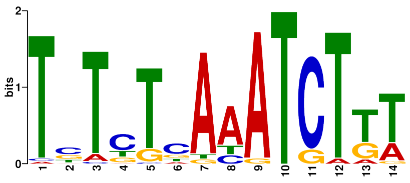 | gimme_82_MDmodule_Motif.14.9 | 7.61E-05 | L3D6_L3D9 |
| 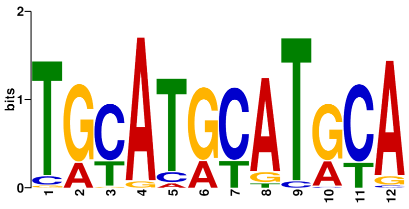 | gimme_73_MDmodule_Motif.12.1 | 7.61E-05 | L3_L4 |

| Motif logo | Motif Name | Motif p_value | Discovery stage |
|---|---|---|---|
| 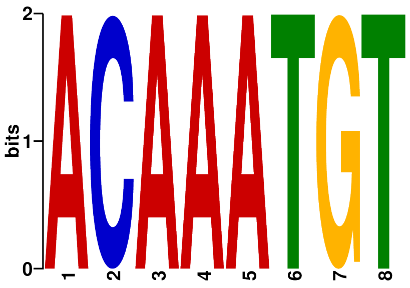 | DME_ACAAATGT | 7.65E-05 | L3D9_L3 |
| 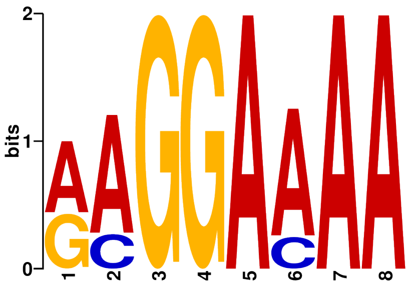 | DME_RMGGAMAA | 7.72E-05 | L3D9_L3D6 |
| 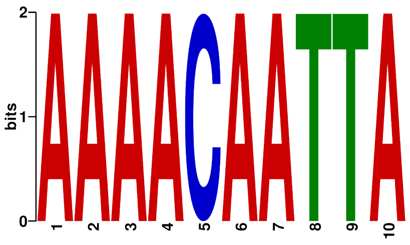 | DME_AAAACAATTA | 7.84E-05 | L3_L3D6 |
| 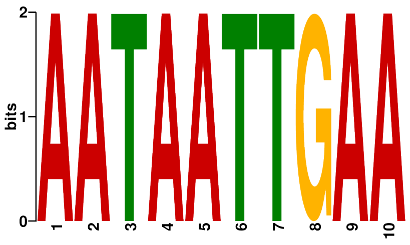 | DME_AATAATTGAA | 7.96E-05 | L3D9_L3D6 |
| 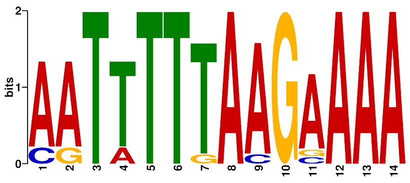 | DME_MRTWTTKAMGVAAA | 8.03E-05 | L3D6_L3 |
| 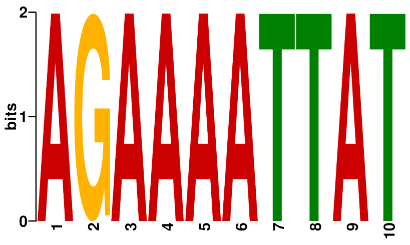 | DME_AGAAAATTAT | 8.27E-05 | L3_L3D9 |
| 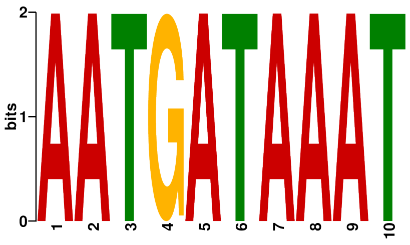 | DME_AATGATAAAT | 8.28E-05 | L3D9_L3 |
| 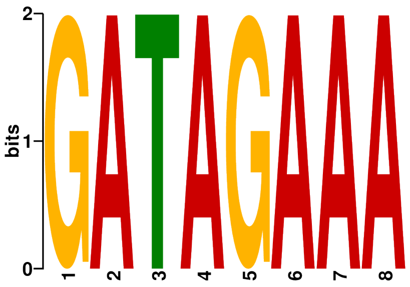 | DME_GATAGAAA | 8.32E-05 | L3D6_L3 |
| 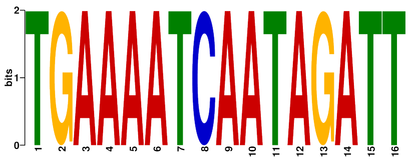 | DME_TGAAAATCAATAGATT | 8.80E-05 | L3D6_L3 |

| Motif logo | Motif Name | Motif p_value | Discovery stage |
|---|---|---|---|
| 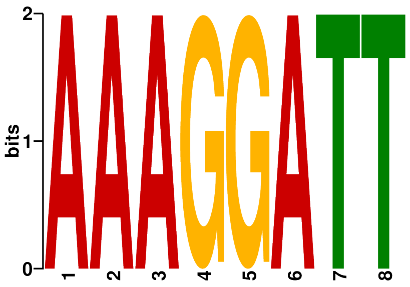 | DME_AAAGGATT | 8.81E-05 | L3D6_L3 |
| 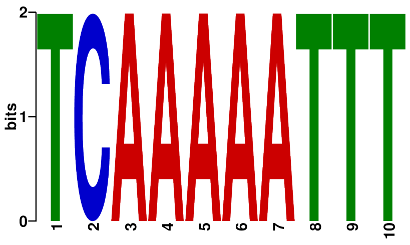 | DME_TCAAAAATTT | 8.91E-05 | L4_L3 |
| 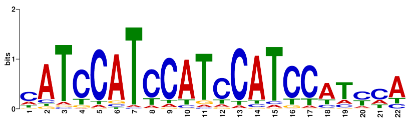 | gimme_53_m1_c1_mATCCATCCATCCATCCATymA | 9.35E-05 | L3D6_L4 |
| 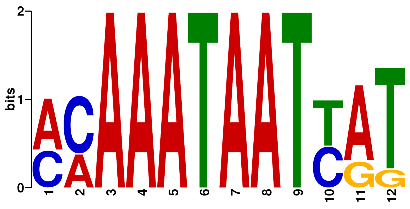 | DME_MMAAATAATYRK | 9.42E-05 | L3_L3D9 |
| 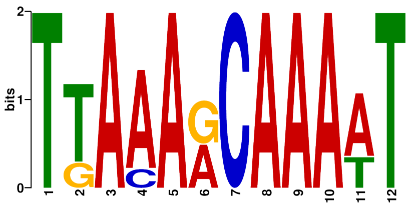 | DME_TKAMARCAAAWT | 9.96E-05 | L3D9_L3 |
| 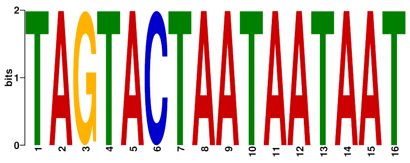 | DME_TAGTACTAATAATAAT | 1.01E-04 | L3D6_L3D9 |
| 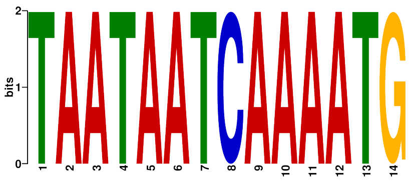 | DME_TAATAATCAAAATG | 1.02E-04 | L3_L3D9 |
| 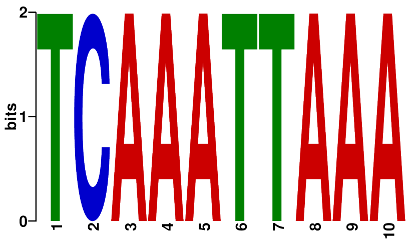 | DME_TCAAATTAAA | 1.04E-04 | L3D6_L4 |
| 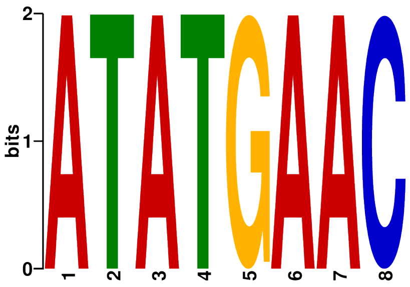 | DME_ATATGAAC | 1.05E-04 | L3_L4 |
| 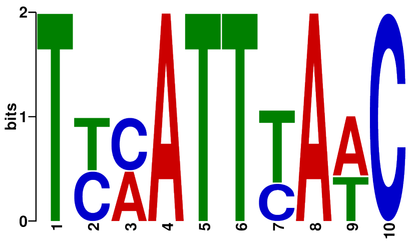 | DME_TYMATTYAWC | 1.05E-04 | L3D6_L3 |

| Motif logo | Motif Name | Motif p_value | Discovery stage |
|---|---|---|---|
| 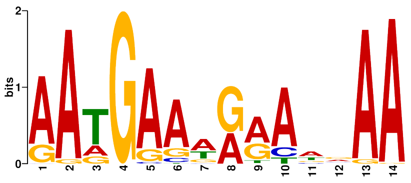 | gimme_151_MEME_6_w14 | 1.06E-04 | L3D6_L4 |
| 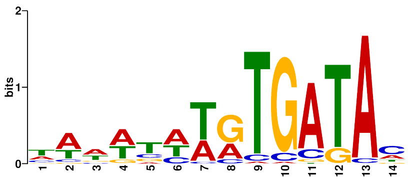 | gimme_59_Improbizer_TAAATATGTGATAC | 1.07E-04 | L4_L3 |
| 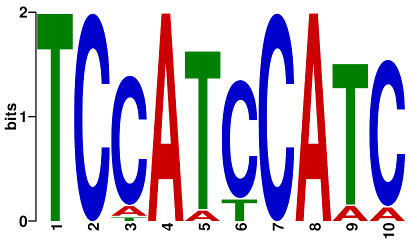 | DME_TCHAWYCAWM | 1.10E-04 | L3D6_L3 |
| 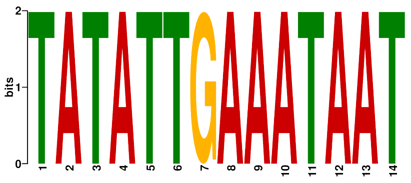 | DME_TATATTGAAATAAT | 1.11E-04 | L3D6_L3D9 |
| 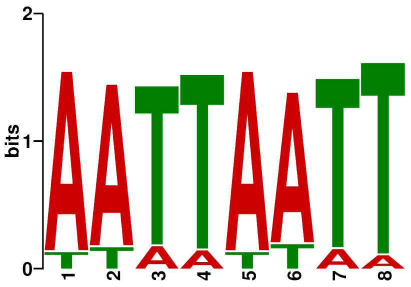 | gimme_175_Weeder_10 | 1.16E-04 | L3_L3D9 |
| 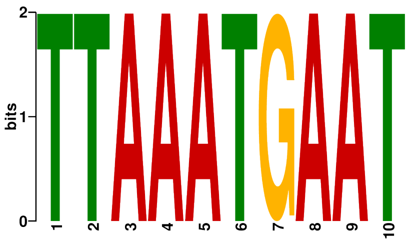 | DME_TTAAATGAAT | 1.18E-04 | L3D6_L3 |
| 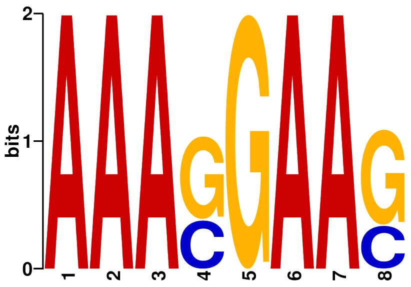 | DME_AAASGAAS | 1.24E-04 | L3D6_L3 |
| 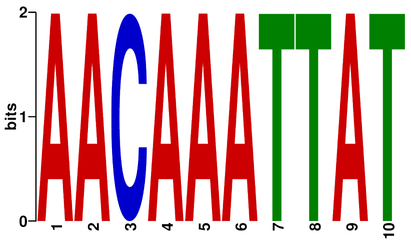 | DME_AACAAATTAT | 1.27E-04 | L3_L3D6 |
| 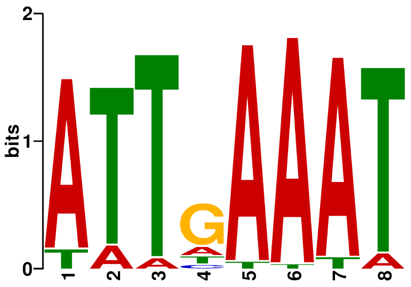 | gimme_172_Weeder_9 | 1.32E-04 | L3D9_L3 |

| Motif logo | Motif Name | Motif p_value | Discovery stage |
| --- | --- | --- | --- |
| 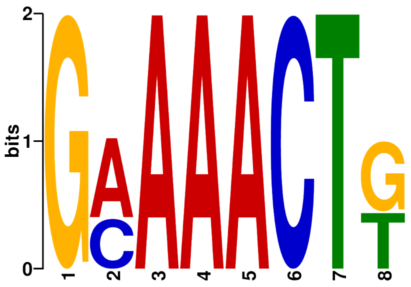 | DME_GMAAACTK | 1.32E-04 | L4_L3D6 |
| 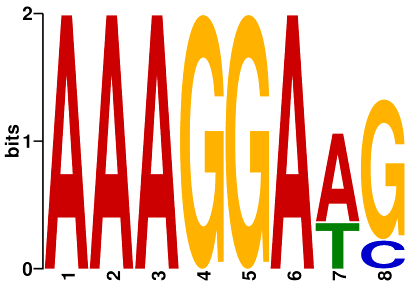 | DME_AAAGGAWS | 1.36E-04 | L3D6_L4 |
| 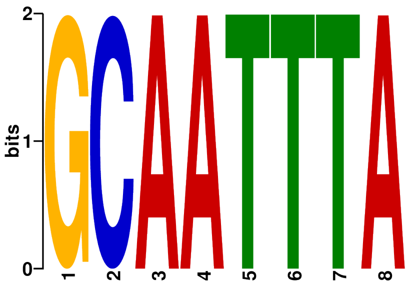 | DME_GCAATTTA | 1.41E-04 | L3D9_L3D6 |
| 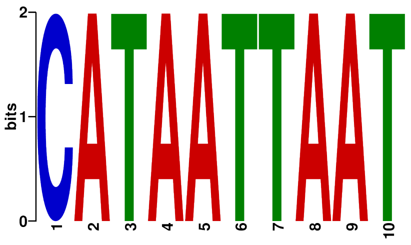 | DME_CATAATTAAT | 1.43E-04 | L4_L3 |
| 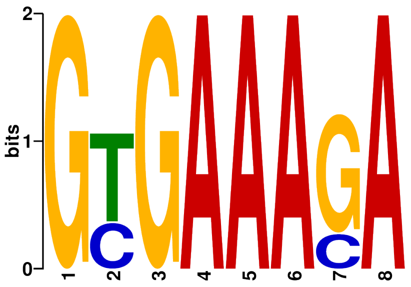 | DME_GYGAAASA | 1.44E-04 | L3D9_L3 |
| 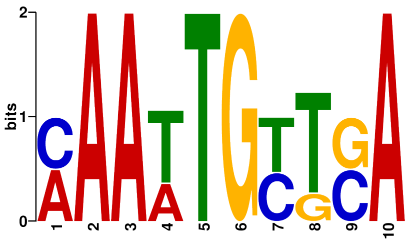 | DME_MAAWTGYKSA | 1.46E-04 | L3D6_L3 |
| 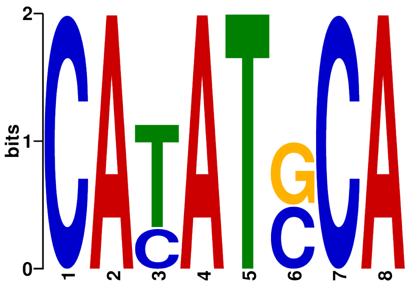 | DME_CAYATSCA | 1.51E-04 | L4_L3 |

| Motif logo | Motif Name | Motif p_value | Discovery stage |
|---|---|---|---|
| 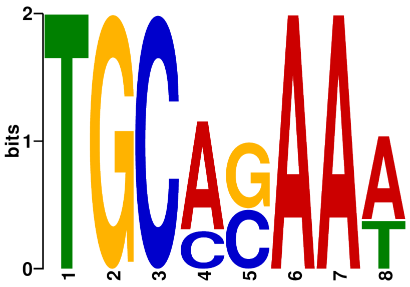 | DME_TGCMCAAW | 1.55E-04 | L3_L3D6 |
| 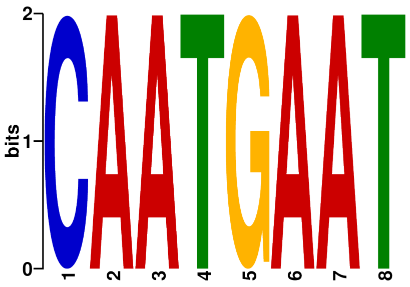 | DME_CAATGAAT | 1.57E-04 | L3D9_L3D6 |
| 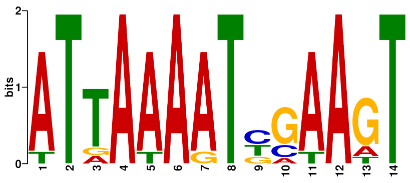 | gimme_49_Homer_14_1 | 1.57E-04 | L3_L3D9 |
| 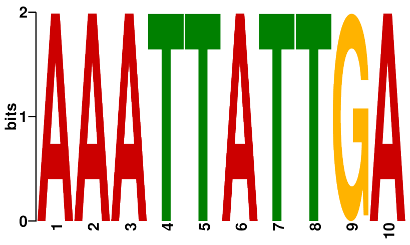 | DME_AAATTATTGA | 1.62E-04 | L4_L3 |
| 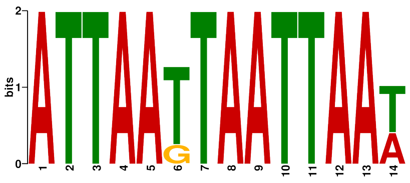 | DME_ATTAAKTAATTAAW | 1.63E-04 | L3D6_L3 |
| 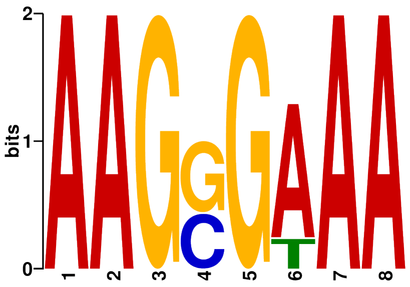 | DME_AAGSGWAA | 1.64E-04 | L4_L3 |
| 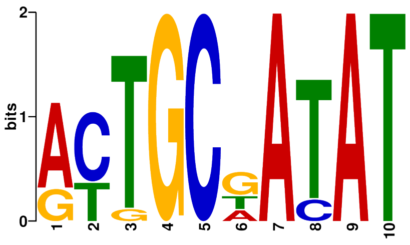 | DME_RYKGCDAYAT | 1.65E-04 | L3_L3D6 |
| 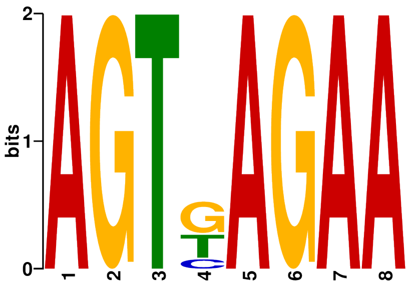 | DME_AGTBAGAA | 1.67E-04 | L3D9_L3 |

| Motif logo | Motif Name | Motif p_value | Discovery stage |
|---|---|---|---|
| 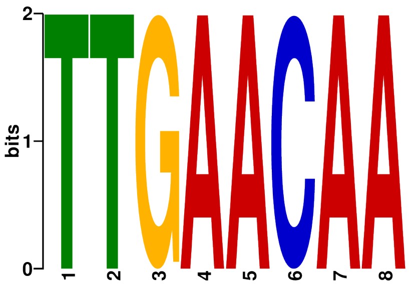 | DME_TTGAACAA.2 | 1.68E-04 | L3D6_L4 |
| 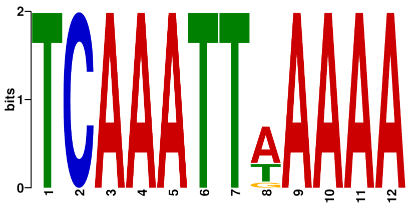 | DME_TCAAATTDAAAA | 1.69E-04 | L3D6_L4 |
| 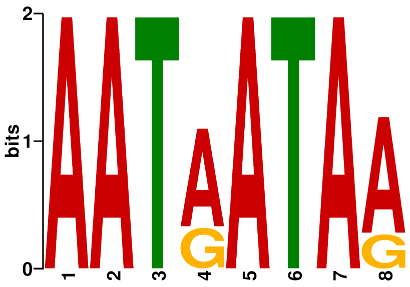 | gimme_15_BioProspector_w8_4 | 1.70E-04 | L3D6_L3D9 |
| 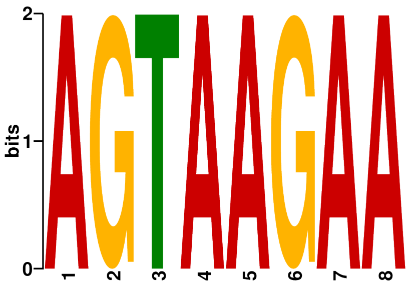 | DME_AGTAAGAA | 1.71E-04 | L3D9_L3 |
| 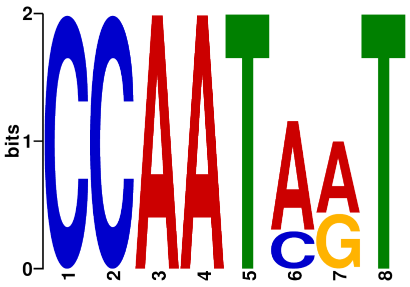 | DME_CCAATMRT | 1.72E-04 | L3D9_L3 |
| 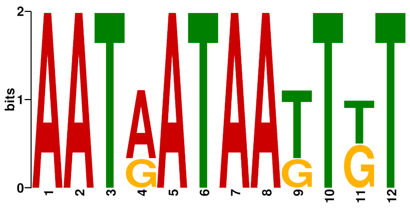 | DME_AATRATAAKTKT | 1.73E-04 | L3_L4 |
| 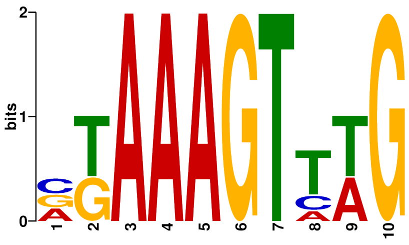 | DME_VKAAAGTHWG | 1.74E-04 | L3_L4 |
| 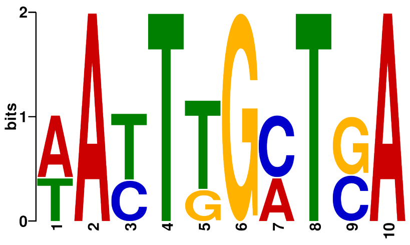 | DME_WAYTKGMTSA | 1.81E-04 | L3D9_L3D6 |

| Motif logo | Motif Name | Motif p_value | Discovery stage |
| --- | --- | --- | --- |
| 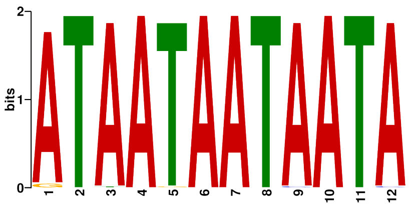 | gimme_41_Homer_12_3 | 1.86E-04 | L3_L3D9 |
| 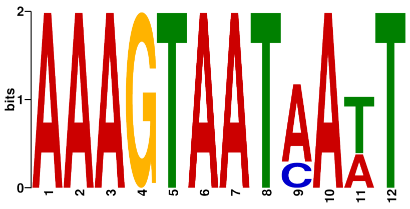 | DME_AAAGTAATMAWT | 1.88E-04 | L3_L3D9 |
| 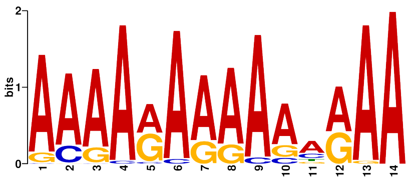 | gimme_124_MEME_2_w14 | 1.90E-04 | L3D9_L3D6 |
| 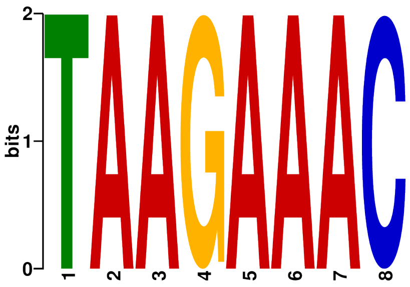 | DME_TAAGAAAC | 1.99E-04 | L4_L3D6 |
| 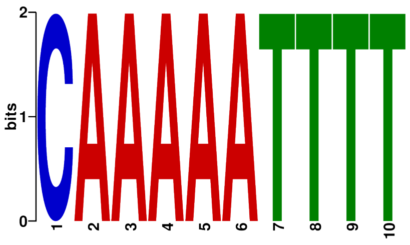 | DME_CAAAAATTTT | 2.01E-04 | L4_L3 |
| 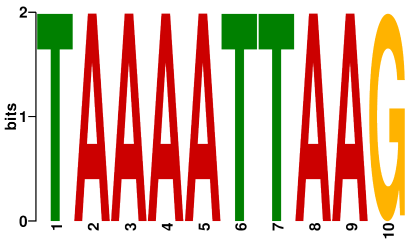 | DME_TAAAATTAAG | 2.05E-04 | L3_L4 |
| 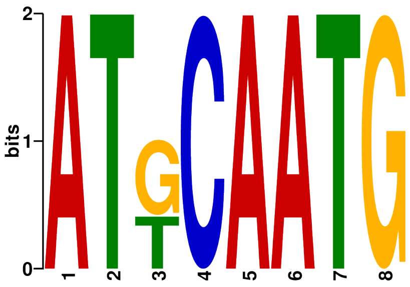 | DME_ATKCAATG | 2.13E-04 | L3D9_L3D6 |
| 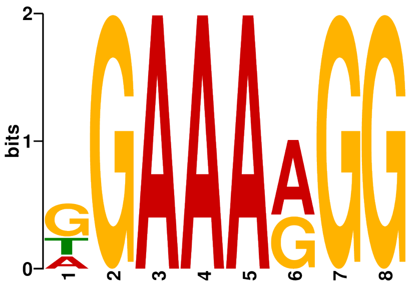 | DME_DGAAARGG | 2.18E-04 | L3D9_L3D6 |
| 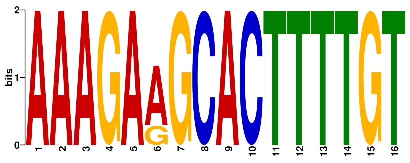 | DME_AAAGARGCACTTTTGT | 2.19E-04 | L3D6_L3 |

| Motif logo | Motif Name | Motif p_value | Discovery stage |
|---|---|---|---|
| 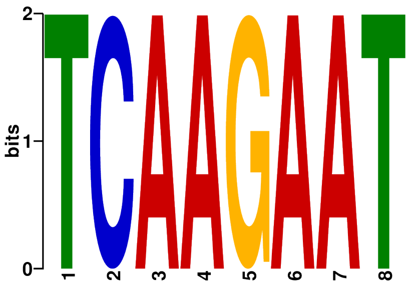 | DME_TCAAGAAT | 2.24E-04 | L4_L3 |
| 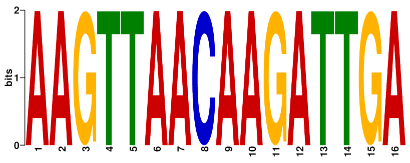 | DME_AAGTTAACAAGATTGA | 2.25E-04 | L3D9_L3D6 |
| 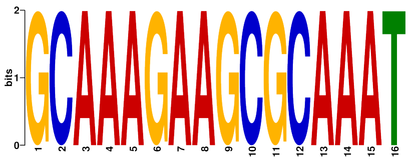 | DME_GCAAAGAAGCGCAAAT | 2.26E-04 | L4_L3D6 |
| 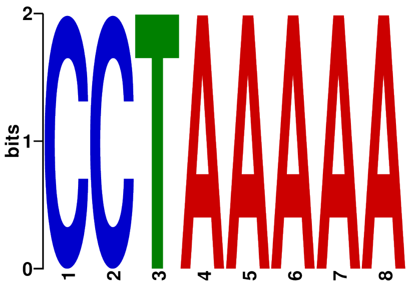 | DME_CCTAAAAA | 2.33E-04 | L3D6_L3D9 |
| 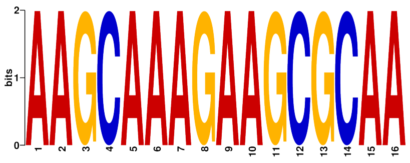 | DME_AAGCAAAGAAGCGCAA | 2.34E-04 | L3D9_L3D6 |
| 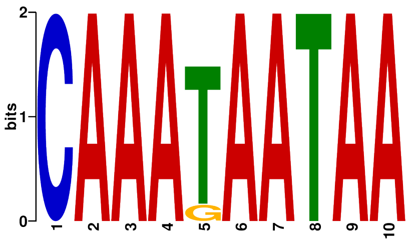 | DME_CAAAKAATAA | 2.40E-04 | L3D9_L3 |
| 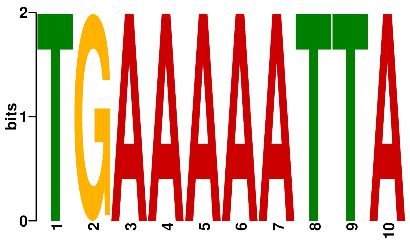 | DME_TGAAAAATTA | 2.69E-04 | L3_L4 |
| 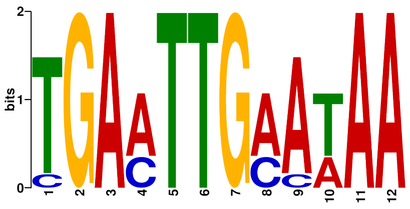 | DME_YGAMTTGMMWAA | 2.72E-04 | L3D9_L3 |
| 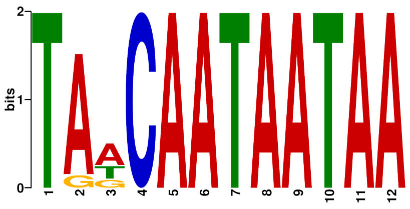 | DME_TRKCAATAATAA | 2.72E-04 | L3D6_L3D9 |

| Motif logo | Motif Name | Motif p_value | Discovery stage |
|---|---|---|---|
| 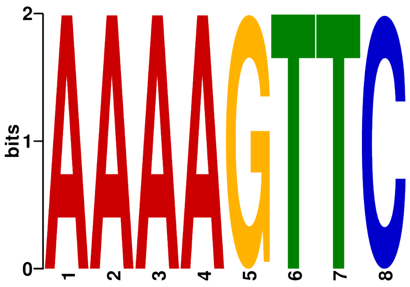 | DME_AAAAGTTC | 2.82E-04 | L3D9_L3D6 |
| 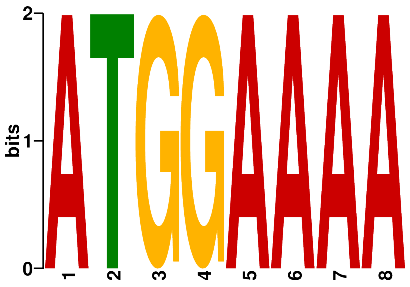 | DME_ATGGAAAA | 2.86E-04 | L4_L3D6 |
| 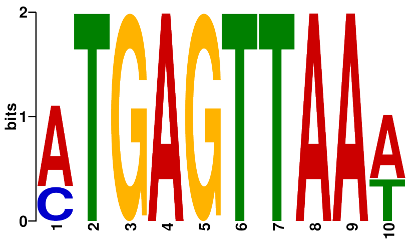 | DME_MTGAGTTAAW | 2.93E-04 | L4_L3 |
| 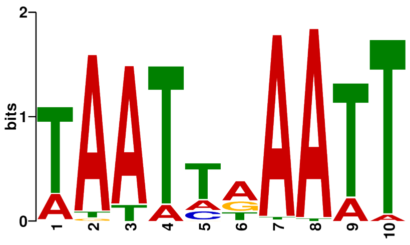 | gimme_176_Weeder_11 | 2.94E-04 | L3_L3D6 |
| 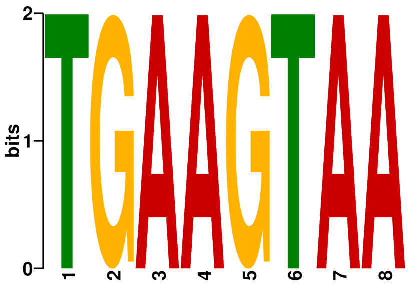 | DME_TGAAGTAA | 2.96E-04 | L3_L3D9 |
| 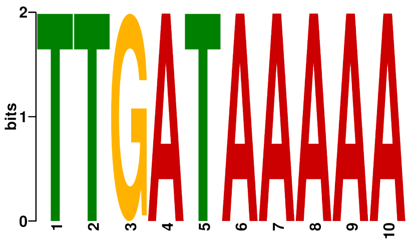 | DME_TTGATAAAAA | 3.00E-04 | L3D9_L3D6 |
| 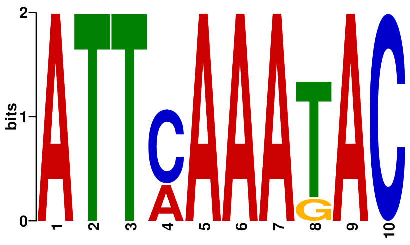 | DME_ATTMAAAKAC | 3.03E-04 | L3D9_L3 |
| 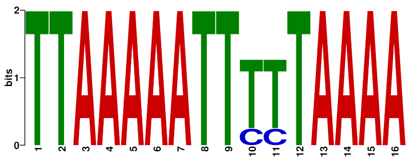 | DME_TTAAAAATTYYTAAAA | 3.05E-04 | L3D9_L3 |

| Motif logo | Motif Name | Motif p_value | Discovery stage |
|---|---|---|---|
| 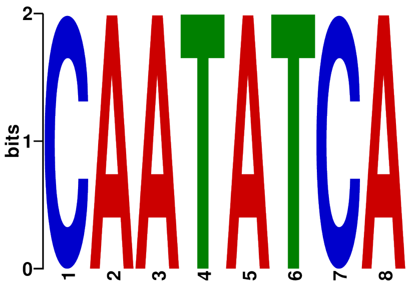 | DME_CAATATCA | 3.17E-04 | L3_L4 |
| 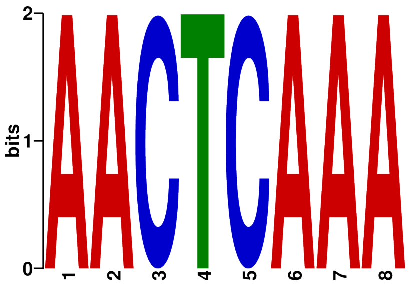 | DME_AACTCAAA | 3.18E-04 | L3_L3D9 |
| 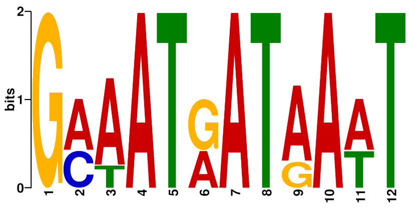 | DME_GMWATRATRAWT | 3.20E-04 | L3D9_L3 |
| 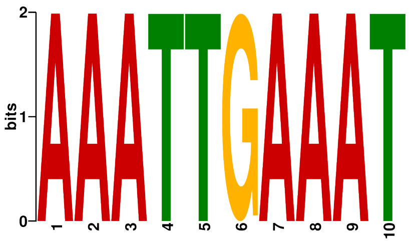 | DME_AAATTGAAAT | 3.20E-04 | L3D6_L3D9 |
| 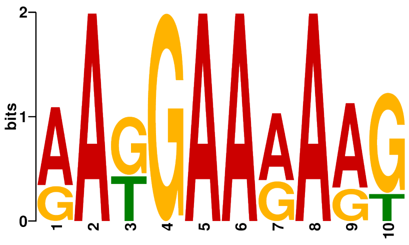 | DME_RAKGAARARK | 3.20E-04 | L4_L3D6 |
| 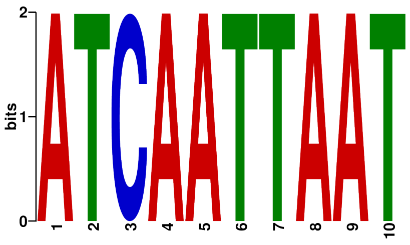 | DME_ATCAATTAAT | 3.20E-04 | L3D9_L3 |
| 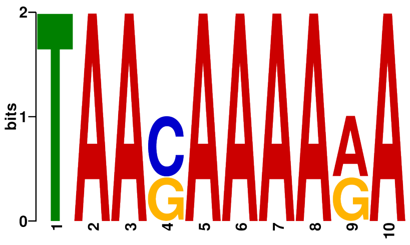 | DME_TAASAAAARA | 3.21E-04 | L3D6_L3D9 |
| 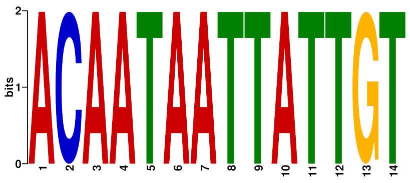 | DME_ACAATAATTATTGT | 3.30E-04 | L4_L3 |
| 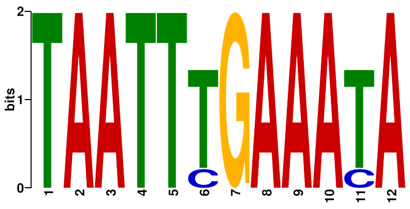 | DME_TAATTYGAAAYA | 3.38E-04 | L3_L4 |

| Motif logo | Motif Name | Motif p_value | Discovery stage |
|---|---|---|---|
| 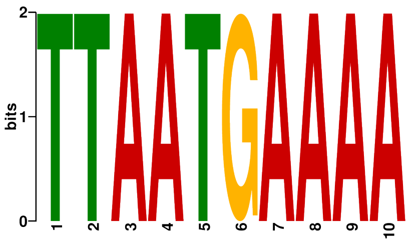 | DME_TTAATGAAAA | 3.40E-04 | L3_L4 |
| 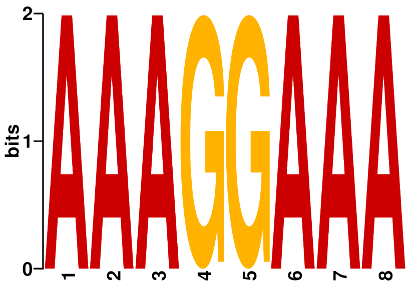 | DME_AAAGGAAA | 3.47E-04 | L3D9_L3D6 |
| 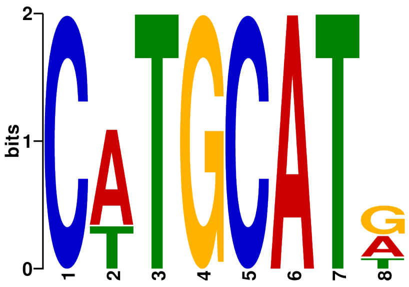 | DME_CWTGCATD | 3.48E-04 | L3D6_L4 |
| 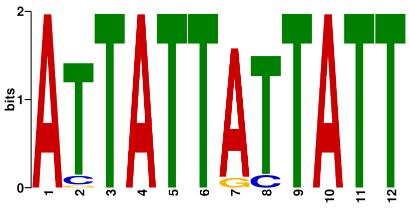 | gimme_19_BioProspector_w12_1 | 3.48E-04 | L3_L3D9 |
| 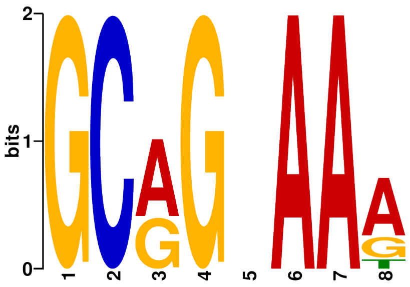 | DME_GCRGNAAR | 3.51E-04 | L3D9_L3D6 |
| 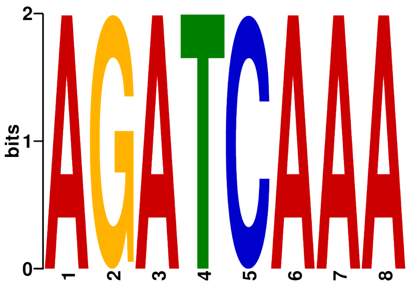 | DME_AGATCAAA | 3.51E-04 | L3D6_L4 |
| 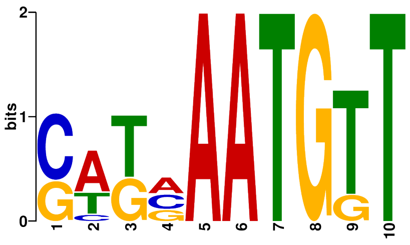 | DME_SHKVAATGKT | 3.54E-04 | L3_L3D9 |
| 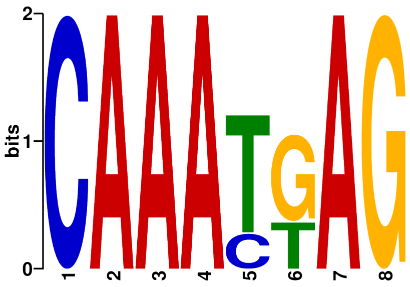 | DME_CAAAYKAG | 3.75E-04 | L3_L4 |

| Motif logo | Motif Name | Motif p_value | Discovery stage |
|---|---|---|---|
| 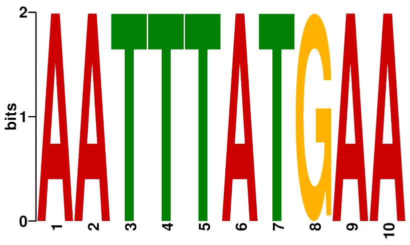 | DME_AATTTATGAA | 3.87E-04 | L3_L4 |
| 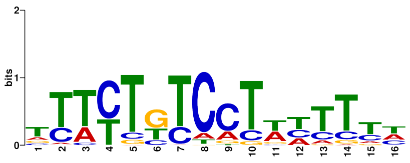 | gimme_55_Improbizer_TTTCTGTCCTTTTTTT | 3.91E-04 | L3D9_L3D6 |
| 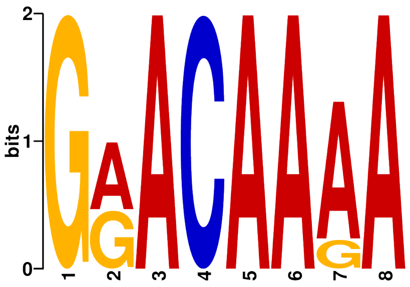 | DME_GRACAARA | 3.96E-04 | L3D9_L3 |
| 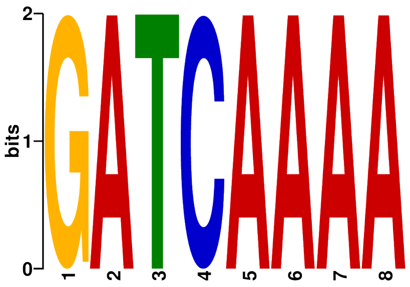 | DME_GATCAAAA | 3.97E-04 | L3D9_L3 |
| 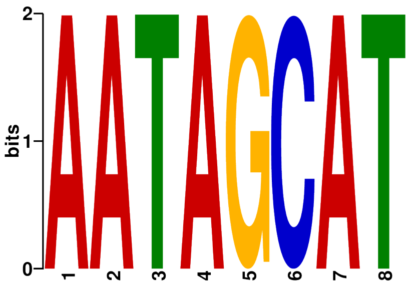 | DME_AATAGCAT | 4.03E-04 | L3_L3D6 |
| 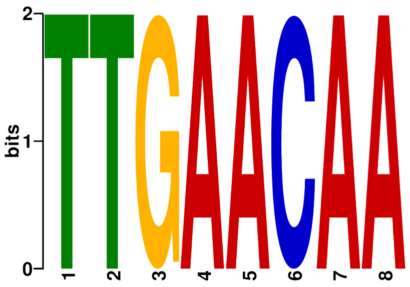 | DME_TTGAACAA.1 | 4.04E-04 | L3D6_L3D9 |
| 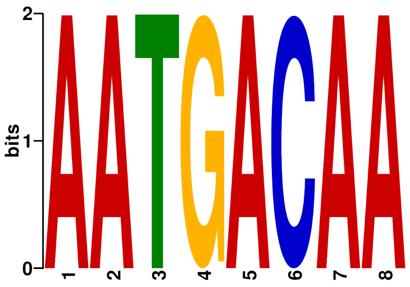 | DME_AATGACAA | 4.06E-04 | L3D6_L3 |
| 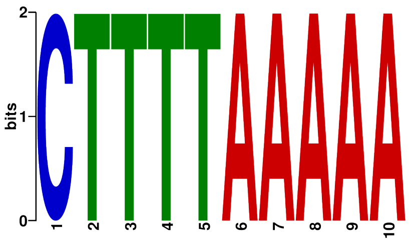 | DME_CTTTTAAAAA | 4.18E-04 | L4_L3 |

| Motif logo | Motif Name | Motif p_value | Discovery stage |
|---|---|---|---|
| 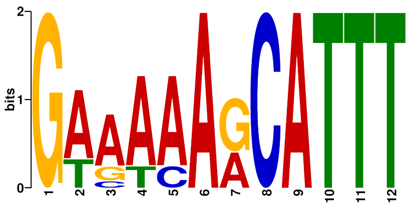 | DME_GWVWMARCATTT | 4.21E-04 | L3_L4 |
| 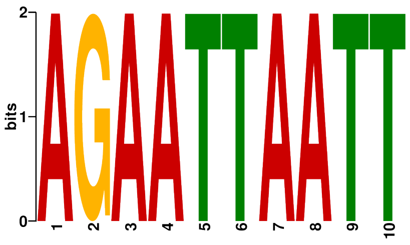 | DME_AGAATTAATT | 4.21E-04 | L3D6_L3D9 |
| 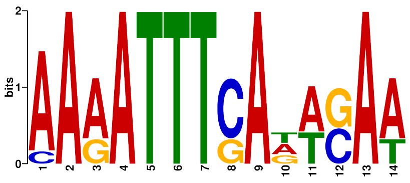 | DME_MARATTTSADWSAW | 4.26E-04 | L3D6_L4 |
| 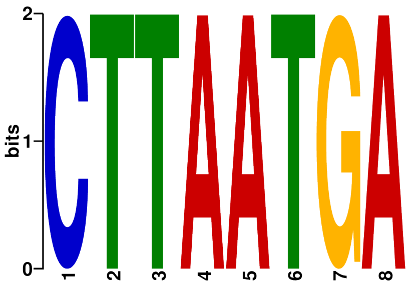 | DME_CTTAATGA | 4.34E-04 | L3D6_L4 |
| 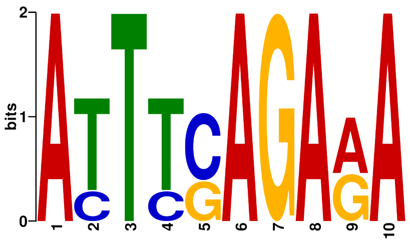 | DME_AYTYSAGARA | 4.40E-04 | L3D6_L4 |
| 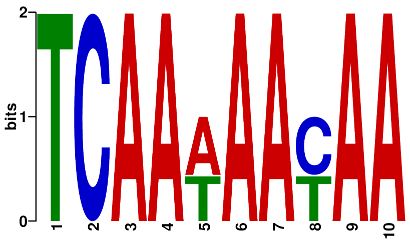 | DME_TCAAWAAYAA | 4.50E-04 | L3_L4 |
| 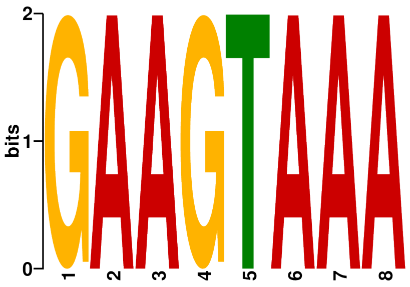 | DME_GAAGTAAA | 4.69E-04 | L3_L4 |
| 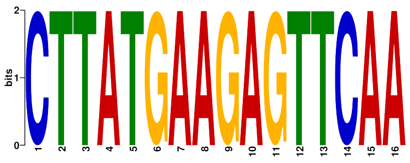 | DME_CTTATGAAGAGTTCAA | 4.74E-04 | L3D6_L3 |
| 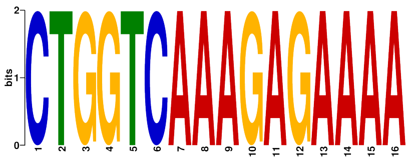 | DME_CTGGTCAAAGAGAAAA.2 | 4.88E-04 | L4_L3D6 |
| 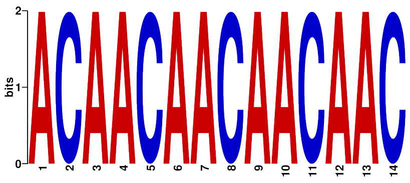 | DME_ACAACAACAACAAC | 4.96E-04 | L3D6_L3D9 |

| Motif logo | Motif Name | Motif p_value | Discovery stage |
|---|---|---|---|
| 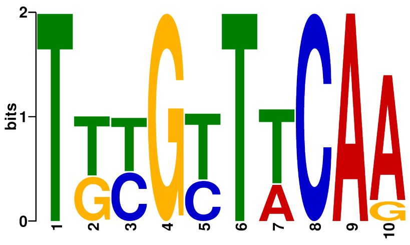 | DME_TKYGYTWCAR | 4.96E-04 | L3_L4 |
| 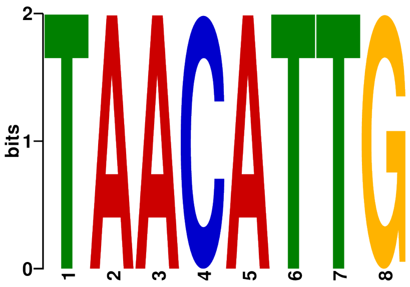 | DME_TAACATTG | 5.10E-04 | L3D6_L4 |
| 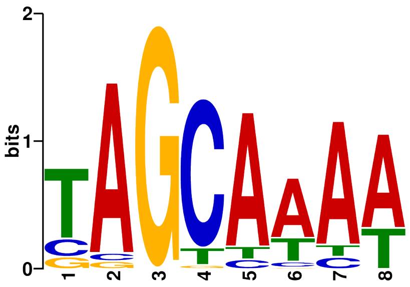 | gimme_59_Improbizer_TAGCAAAA | 5.15E-04 | L3_L4 |
| 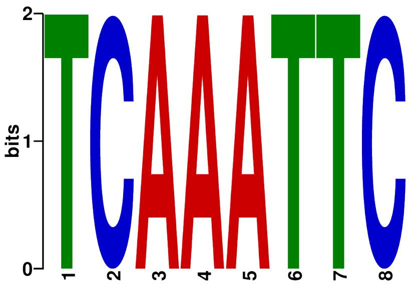 | DME_TCAAATTC | 5.29E-04 | L3D9_L3 |
| 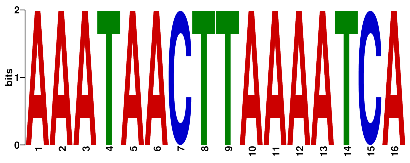 | DME_AAATAACTTAAAATCA | 5.33E-04 | L3D6_L3D9 |
| 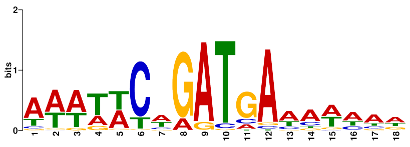 | gimme_57_Improbizer_AAATTCAGATGAAAAAAA | 5.61E-04 | L3D6_L3D9 |
| 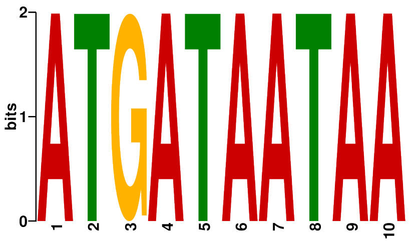 | DME_ATGATAATAA | 5.73E-04 | L3D9_L3 |
| 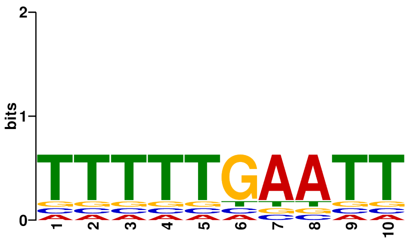 | gimme_36_Homer_10_3 | 5.81E-04 | L4_L3 |
| 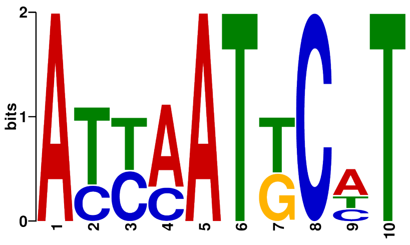 | DME_AYYMATKCHT | 5.88E-04 | L3D9_L3D6 |

| Motif logo | Motif Name | Motif p_value | Discovery stage |
|---|---|---|---|
| 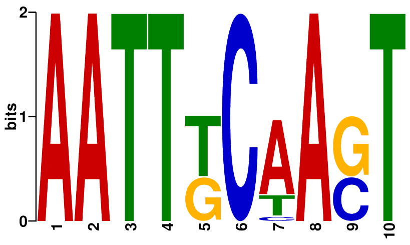 | DME_AATTKCHAST | 5.89E-04 | L3D9_L3 |
| 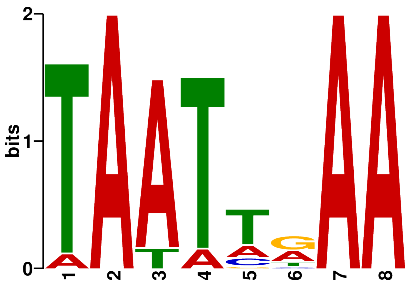 | gimme_177_Weeder_12 | 5.93E-04 | L3_L3D9 |
| 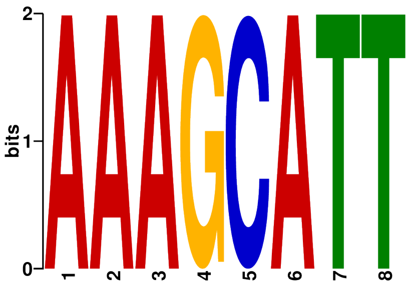 | DME_AAAGCATT | 5.96E-04 | L3_L4 |
| 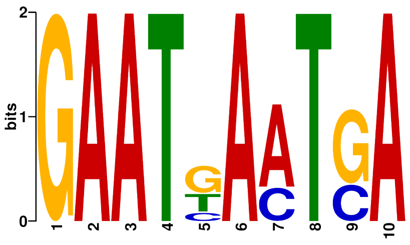 | DME_GAATBAMTSA | 6.01E-04 | L3_L3D6 |
| 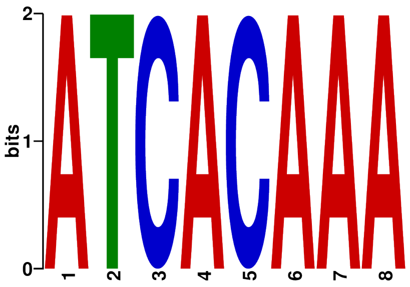 | DME_ATCACAAA | 6.16E-04 | L3D9_L3D6 |
| 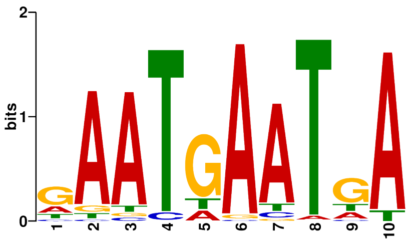 | gimme_67_MDmodule_Motif.10.5 | 6.18E-04 | L3_L4 |
| 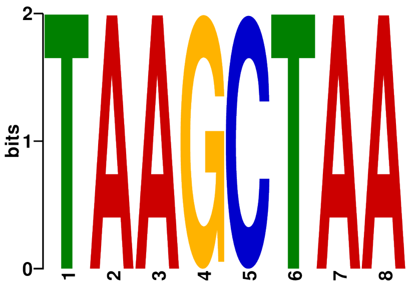 | DME_TAAGCTAA | 6.24E-04 | L3D9_L3 |
| 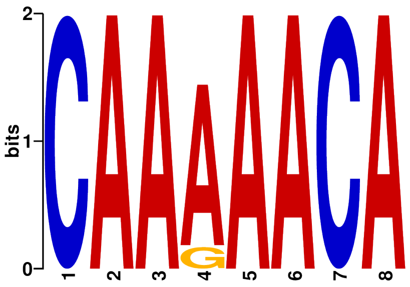 | DME_CAARAACA | 6.28E-04 | L3_L4 |

| Motif logo | Motif Name | Motif p_value | Discovery stage |
|---|---|---|---|
| 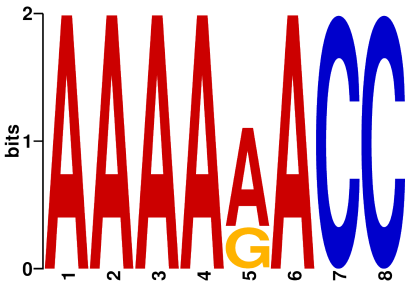 | DME_AAAARACC | 6.29E-04 | L3_L4 |
| 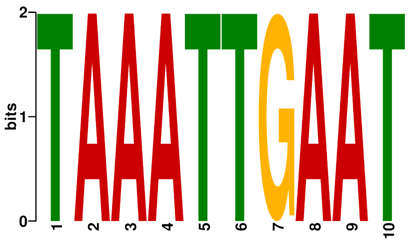 | DME_TAAATTGAAT | 6.31E-04 | L4_L3D6 |
| 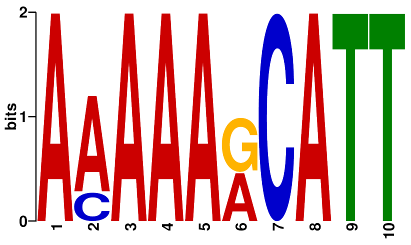 | DME_AMAAARCATT | 6.53E-04 | L3_L3D9 |
| 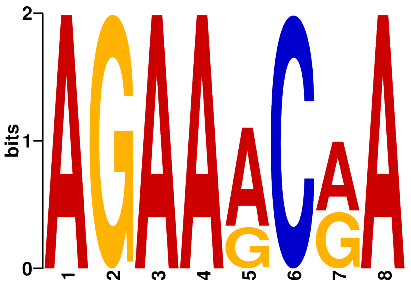 | DME_AGAARCRA | 6.53E-04 | L4_L3D6 |
| 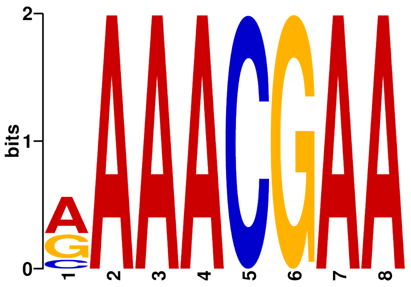 | DME_VAAACGAA | 6.85E-04 | L3D6_L3 |
| 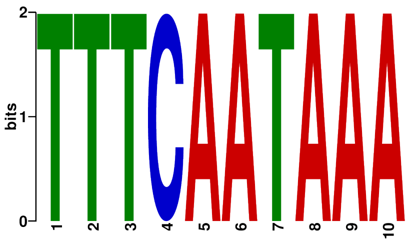 | DME_TTTCAATAAA | 7.00E-04 | L3D9_L3 |
| 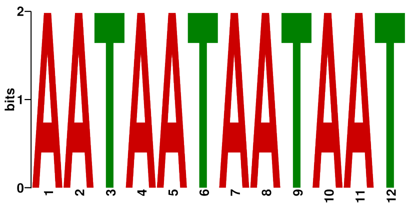 | DME_AATAATAATAAT | 7.04E-04 | L3D6_L3D9 |
| 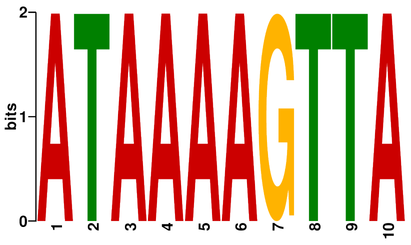 | DME_ATAAAAGTTA | 7.46E-04 | L4_L3D6 |

| Motif logo | Motif Name | Motif p_value | Discovery stage |
|---|---|---|---|
| 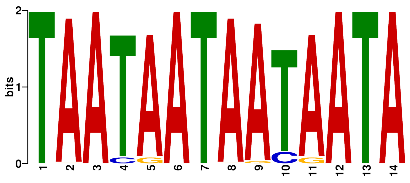 | gimme_10_BioProspector_w14_3 | 7.51E-04 | L3D6_L4 |
| 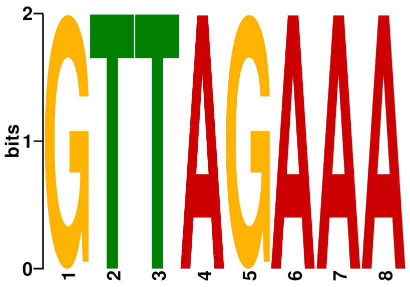 | DME_GTTAGAAA | 7.52E-04 | L3_L3D9 |
| 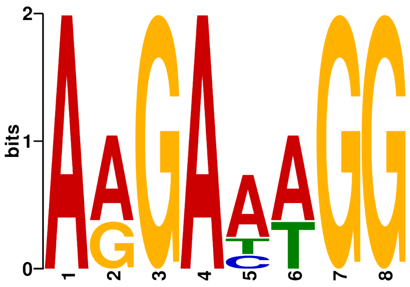 | DME_ARGAHWGG | 7.53E-04 | L3D9_L3 |
| 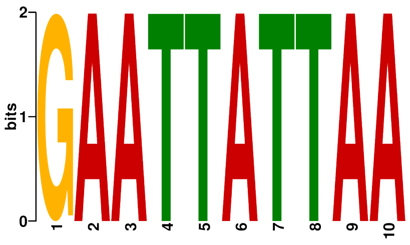 | DME_GAATTATTAA | 7.76E-04 | L3_L3D6 |
| 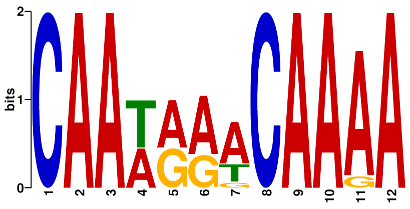 | DME_CAAWRRDCAARA | 7.98E-04 | L3D6_L3 |
| 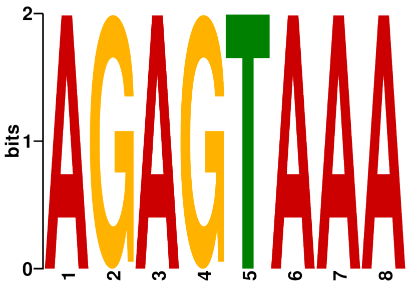 | DME_AGAGTAAA | 7.98E-04 | L3D6_L3 |
| 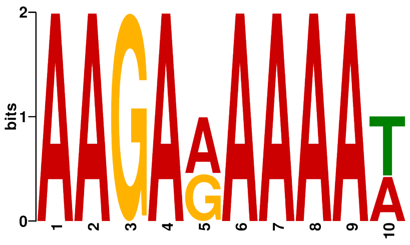 | DME_AAGARAAAAW | 8.14E-04 | L3D9_L3D6 |
| 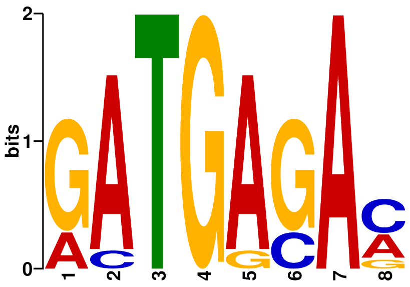 | DECOD_Motif9_8 | 8.19E-04 | L4_L3D6 |

| Motif logo | Motif Name | Motif p_value | Discovery stage |
|---|---|---|---|
| 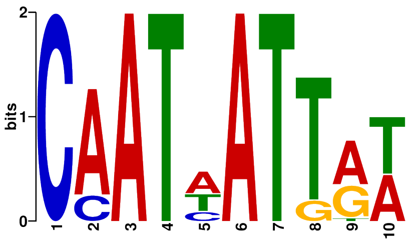 | DECOD_Motif10_10.3 | 8.27E-04 | L4_L3 |
| 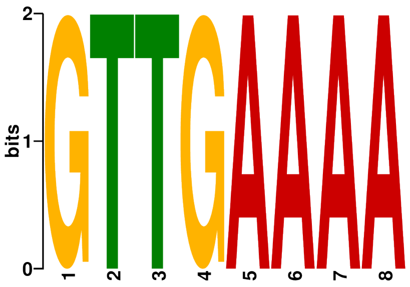 | DME_GTTGAAAA | 8.38E-04 | L4_L3D6 |
| 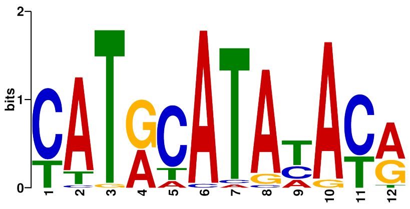 | gimme_108_MDmodule_Motif.12.6 | 8.59E-04 | L3_L4 |
| 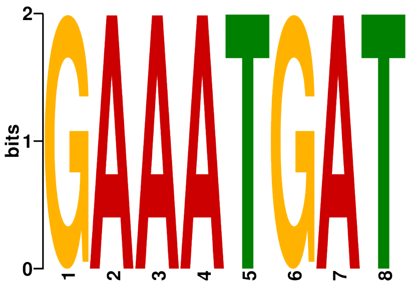 | DME_GAAATGAT | 8.66E-04 | L4_L3D6 |
| 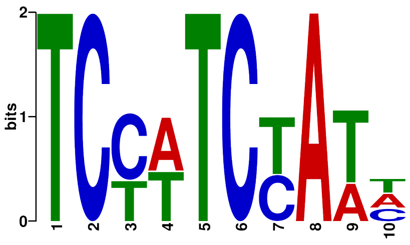 | DME_TCYWTCYAWH | 8.82E-04 | L3_L3D6 |
| 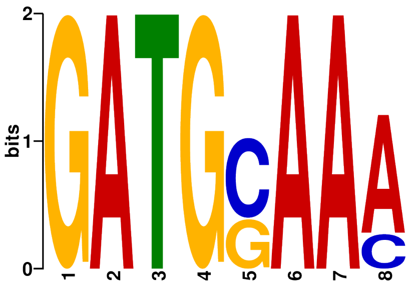 | DME_GATGSAAM | 9.43E-04 | L4_L3D6 |
| 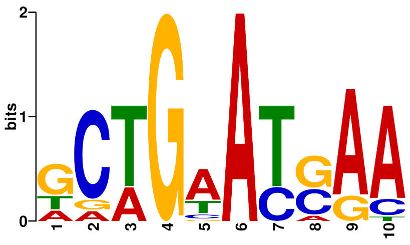 | DECOD_Motif6_10 | 9.44E-04 | L3_L3D6 |
| 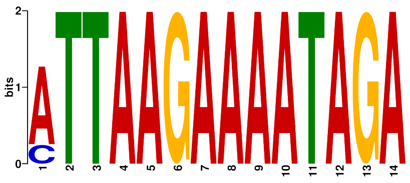 | DME_MTTAAGAAAATAGA | 9.45E-04 | L3D6_L3 |
| 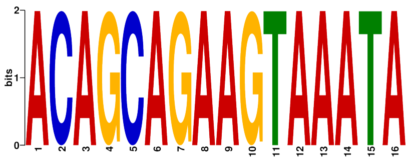 | DME_ACAGCAGAAGTAAATA | 9.85E-04 | L4_L3D6 |

| Motif logo | Motif Name | Motif p_value | Discovery stage |
|---|---|---|---|
| 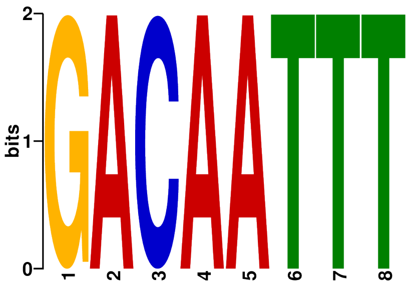 | DME_GACAATTT | 9.89E-04 | L3_L3D9 |
| 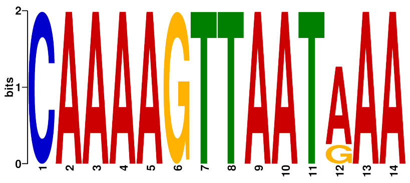 | DME_CAAAAGTTAATRAA | 9.91E-04 | L3D6_L3 |
